# Supplementary material for: Genetic diversity and phylogeographic patterns of the peacock jewel-damselfly, Rhinocypha fenestrella (Rambur, 1842)
Source: PLoS One. 2024 Apr 5;19(4):e0301392. doi: 10.1371/journal.pone.0301392 (PMC10997100; doi:10.1371/journal.pone.0301392)
Supplement: S1 Table — (PDF) [file pone.0301392.s001.pdf]

S1 Table. Percentage (%) of uncorrected "*p*" distance matrix among the 26 representative *cox1* haplotypes of *Rhinocypha fenestrella* in Malaysia.

Haplotypes	A1	A2	A3	A4	A5	A6	A7	A8	A9	A10	A11	A12	A13	A14	A15	A16	A17	A18	A19	A20	A21	A22	A23	A24	A25	A26
**A1**	-																									
**A2**	0.16	-																								
**A3**	0.16	0.33	-																							
**A4**	1.14	0.98	1.30	-																						
**A5**	1.30	1.14	1.47	0.16	-																					
**A6**	0.98	0.81	1.14	0.16	0.33	-																				
**A7**	1.30	1.14	1.47	0.16	0.33	0.33	-																			
**A8**	0.33	0.16	0.49	1.14	1.30	0.98	1.30	-																		
**A9**	0.33	0.16	0.49	1.14	1.30	0.98	1.30	0.33	-																	
**A10**	0.49	0.33	0.65	1.30	1.47	1.14	1.47	0.49	0.49	-																
**A11**	0.49	0.33	0.65	1.30	1.47	1.14	1.47	0.49	0.49	0.65	-															
**A12**	0.33	0.16	0.49	1.14	1.30	0.98	1.30	0.33	0.33	0.16	0.49	-														
**A13**	0.33	0.16	0.49	1.14	1.30	0.98	1.30	0.33	0.33	0.49	0.49	0.33	-													
**A14**	0.49	0.33	0.65	1.30	1.47	1.14	1.47	0.49	0.49	0.65	0.65	0.49	0.49	-												
**A15**	0.65	0.49	0.81	1.47	1.63	1.30	1.63	0.65	0.65	0.81	0.81	0.65	0.65	0.16	-											
**A16**	0.33	0.16	0.49	1.14	1.30	0.98	1.30	0.33	0.33	0.49	0.49	0.33	0.33	0.49	0.65	-										
**A17**	0.65	0.49	0.81	1.47	1.63	1.30	1.63	0.65	0.65	0.81	0.81	0.65	0.65	0.16	0.33	0.65	-									
**A18**	0.33	0.16	0.49	1.14	1.30	0.98	1.30	0.33	0.33	0.49	0.49	0.33	0.33	0.49	0.65	0.33	0.65	-								
**A19**	0.65	0.49	0.81	1.47	1.63	1.30	1.63	0.65	0.65	0.81	0.81	0.65	0.65	0.16	0.33	0.65	0.33	0.65	-							
**A20**	0.16	0.33	0.33	1.30	1.47	1.14	1.47	0.49	0.49	0.65	0.65	0.49	0.49	0.65	0.81	0.49	0.81	0.49	0.81	-						
**A21**	0.33	0.16	0.49	1.14	1.30	0.98	1.30	0.33	0.33	0.49	0.16	0.33	0.33	0.49	0.65	0.33	0.65	0.33	0.65	0.49	-					
**A22**	0.33	0.16	0.49	1.14	1.30	0.98	1.30	0.33	0.33	0.49	0.49	0.33	0.33	0.49	0.65	0.33	0.65	0.33	0.65	0.49	0.33	-				
**A23**	0.49	0.33	0.65	1.30	1.47	1.14	1.47	0.16	0.49	0.65	0.33	0.49	0.49	0.65	0.81	0.49	0.81	0.49	0.81	0.65	0.16	0.49	-			
**A24**	0.49	0.33	0.65	1.30	1.47	1.14	1.47	0.49	0.49	0.33	0.65	0.16	0.49	0.65	0.81	0.49	0.81	0.49	0.81	0.65	0.49	0.49	0.65	-		
**A25**	0.49	0.33	0.65	1.30	1.47	1.14	1.47	0.49	0.49	0.65	0.33	0.49	0.49	0.65	0.81	0.49	0.81	0.49	0.81	0.65	0.16	0.49	0.33	0.33	-	
**A26**	0.49	0.33	0.65	1.30	1.47	1.14	1.47	0.49	0.49	0.65	0.33	0.49	0.49	0.65	0.81	0.49	0.81	0.49	0.81	0.65	0.16	0.49	0.33	0.65	0.33	-
