# Supplementary material for: Genetic diversity and phylogeographic patterns of the peacock jewel-damselfly, Rhinocypha fenestrella (Rambur, 1842)
Source: PLoS One. 2024 Apr 5;19(4):e0301392. doi: 10.1371/journal.pone.0301392 (PMC10997100; doi:10.1371/journal.pone.0301392)
Supplement: S2 Table — (PDF) [file pone.0301392.s002.pdf]

S2 Table. Percentage (%) of uncorrected "*p*" distance matrix among the 10 representative *16S rRNA* haplotypes of *Rhinocypha fenestrella* in Malaysia.

Haplotypes	B1	B2	B3	B4	B5	B6	B7	B8	B9	B10
**B1**	-									
**B2**	0.19	-								
**B3**	0.19	0.38	-							
**B4**	0.19	0.38	0.38	-						
**B5**	0.38	0.56	0.56	0.38	-					
**B6**	0.19	0.38	0.38	0.38	0.56	-				
**B7**	0.38	0.56	0.56	0.56	0.75	0.56	-			
**B8**	0.19	0.38	0.38	0.38	0.56	0.38	0.56	-		
**B9**	0.01	0.19	0.19	0.19	0.38	0.19	0.39	0.19	-	
**B10**	0.19	0.38	0.38	0.38	0.56	0.38	0.56	0.38	0.19	-
