# Supplementary material for: Genetic diversity and phylogeographic patterns of the peacock jewel-damselfly, Rhinocypha fenestrella (Rambur, 1842)
Source: PLoS One. 2024 Apr 5;19(4):e0301392. doi: 10.1371/journal.pone.0301392 (PMC10997100; doi:10.1371/journal.pone.0301392)
Supplement: S3 Table — (PDF) [file pone.0301392.s003.pdf]

S3 Table. Percentage (%) of uncorrected "*p*" distance matrix among the 32 representatives for the concatenated *cox1+16S* haplotypes of *Rhinocypha fenestrella* in Malaysia.

	AB 1	AB 2	AB 3	AB 4	AB 5	AB 6	AB 7	AB 8	AB 9	AB 10	AB 11	AB 12	AB 13	AB 14	AB 15	AB 16	AB 17	AB 18	AB 19	AB 20	AB 21	AB 22	AB 23	AB 24	AB 25	AB 26	AB 27	AB 28	AB 29	AB 30	AB 31
**AB1**																															
**AB2**	0.09																														
**AB3**	0.09	0.17																													
**AB4**	0.09	0.17	0.17																												
**AB5**	0.61	0.53	0.70	0.70																											
**AB6**	0.70	0.61	0.79	0.79	0.09																										
**AB7**	0.53	0.44	0.61	0.61	0.09	0.17																									
**AB8**	0.70	0.61	0.79	0.79	0.09	0.17	0.17																								
**AB9**	0.17	0.09	0.26	0.26	0.61	0.70	0.53	0.70																							
**AB10**	0.17	0.09	0.26	0.26	0.61	0.70	0.53	0.70	0.17																						
**AB11**	0.17	0.09	0.26	0.26	0.61	0.70	0.53	0.70	0.17	0.17																					
**AB12**	0.26	0.17	0.35	0.35	0.70	0.79	0.61	0.79	0.26	0.26	0.26																				
**AB13**	0.26	0.17	0.35	0.35	0.70	0.79	0.61	0.79	0.26	0.26	0.26	0.35																			
**AB14**	0.17	0.09	0.26	0.26	0.61	0.70	0.53	0.70	0.17	0.17	0.17	0.09	0.26																		
**AB15**	0.17	0.09	0.26	0.26	0.61	0.70	0.53	0.70	0.17	0.18	0.17	0.26	0.26	0.18																	
**AB16**	0.26	0.17	0.35	0.35	0.70	0.79	0.61	0.79	0.26	0.26	0.26	0.35	0.35	0.26	0.26																
**AB17**	0.35	0.26	0.44	0.44	0.79	0.88	0.70	0.88	0.35	0.35	0.35	0.44	0.44	0.35	0.35	0.09															
**AB18**	0.35	0.26	0.44	0.44	0.79	0.88	0.70	0.88	0.35	0.35	0.35	0.44	0.44	0.35	0.35	0.09	0.17														
**AB19**	0.17	0.09	0.26	0.26	0.61	0.70	0.53	0.70	0.17	0.17	0.17	0.26	0.26	0.17	0.18	0.26	0.35	0.35													
**AB20**	0.35	0.26	0.44	0.44	0.79	0.88	0.70	0.88	0.35	0.35	0.35	0.44	0.44	0.35	0.35	0.44	0.53	0.44	0.18												
**AB21**	0.26	0.17	0.35	0.35	0.70	0.79	0.61	0.79	0.26	0.26	0.26	0.35	0.35	0.26	0.26	0.35	0.44	0.44	0.09	0.26											
**AB22**	0.35	0.26	0.44	0.44	0.79	0.88	0.70	0.88	0.35	0.35	0.35	0.44	0.44	0.35	0.35	0.09	0.17	0.17	0.35	0.53	0.44										
**AB23**	0.44	0.35	0.53	0.53	0.88	**0.97**	0.79	**0.97**	0.44	0.44	0.44	0.53	0.53	0.44	0.44	0.18	0.26	0.26	0.44	0.62	0.53	0.26									
**AB24**	0.26	0.17	0.35	0.35	0.70	0.79	0.61	0.79	0.26	0.26	0.26	0.35	0.35	0.26	0.26	0.35	0.44	0.44	0.26	0.44	0.35	0.44	0.53								
**AB25**	0.44	0.35	0.53	0.53	0.88	0.97	0.79	**0.97**	0.44	0.44	0.44	0.53	0.53	0.44	0.44	0.17	0.26	0.26	0.44	0.62	0.53	0.26	0.35	0.53							
**AB26**	0.09	0.17	0.17	0.17	0.70	0.79	0.61	0.79	0.26	0.26	0.26	0.35	0.35	0.26	0.26	0.35	0.44	0.44	0.26	0.44	0.35	0.44	0.53	0.35	0.53						
**AB27**	0.17	0.09	0.26	0.26	0.61	0.70	0.53	0.70	0.17	0.17	0.17	0.26	0.09	0.17	0.17	0.26	0.35	0.35	0.17	0.35	0.26	0.35	0.44	0.26	0.44	0.26					
**AB28**	0.17	0.09	0.26	0.26	0.61	0.70	0.53	0.70	0.17	0.17	0.17	0.26	0.26	0.17	0.17	0.26	0.35	0.35	0.17	0.35	0.26	0.35	0.44	0.26	0.44	0.26	0.17				
**AB29**	0.26	0.17	0.35	0.35	0.70	0.79	0.61	0.79	0.26	0.09	0.26	0.35	0.17	0.26	0.26	0.35	0.44	0.44	0.26	0.44	0.35	0.44	0.53	0.35	0.53	0.35	0.09	0.26			
**AB30**	0.26	0.18	0.35	0.35	0.70	0.79	0.62	0.79	0.26	0.26	0.26	0.17	0.35	0.09	0.26	0.35	0.44	0.44	0.26	0.44	0.35	0.44	0.53	0.35	0.53	0.35	0.26	0.26	0.35		
**AB31**	0.26	0.17	0.35	0.35	0.70	0.79	0.61	0.79	0.26	0.26	0.26	0.35	0.17	0.26	0.26	0.35	0.44	0.44	0.26	0.44	0.35	0.44	0.53	0.35	0.53	0.35	0.09	0.26	0.18	0.17	
**AB32**	0.26	0.17	0.35	0.35	0.70	0.79	0.61	0.79	0.26	0.26	0.26	0.35	0.17	0.26	0.26	0.35	0.44	0.44	0.26	0.44	0.35	0.44	0.53	0.35	0.53	0.35	0.09	0.26	0.17	0.35	0.17
