# Supplementary material for: Genetic diversity and phylogeographic patterns of the peacock jewel-damselfly, Rhinocypha fenestrella (Rambur, 1842)
Source: PLoS One. 2024 Apr 5;19(4):e0301392. doi: 10.1371/journal.pone.0301392 (PMC10997100; doi:10.1371/journal.pone.0301392)
Supplement: Attachment — Submitted filename: PONE-D-23-28970_reviewer_af.pdf [file pone.0301392.s004.pdf]

PLOS ONE

Genetic diversity and phylogeographic patterns of the peacock jewel-damselfly, Rhinocypha fenestrella (Odonata: Chlorocyphidae)

--Manuscript Draft--

**Manuscript Number:**	PONE-D-23-28970
**Article Type:**	Research Article
**Full Title:**	Genetic diversity and phylogeographic patterns of the peacock jewel-damselfly, Rhinocypha fenestrella (Odonata: Chlorocyphidae)
**Short Title:**	Genetic diversity and phylogeography of Rhinocypha fenestrella
**Corresponding Author:**	Noor Azrizal Abdul Wahid, Ph.D<br>Universiti Putra Malaysia Fakulti Sains<br>Serdang, Selangor MALAYSIA
**Keywords:**	cox1, haplotype, Malaysia, population structure, Rhinocypha fenestrella, 16S,
**Abstract:**	The present study examined the genetic diversity and phylogeographic pattern of the peacock jewel-damselfly Rhinocypha fenestrella by sequencing the cytochrome C oxidase I (cox1) and 16S rRNA gene regions from 147 individuals representing eight populations in Malaysia. A total of 26 and 10 unique haplotypes were revealed by the cox1 and 16S rRNA genes, respectively, and 32 haplotypes were recovered by the concatenated sequences of cox1+16S. Analyses indicated that haplotype AB2 was the most frequent and the most widespread haplotype in Malaysia, and the most recent common ancestor of the R. fenestrella may exist from the Negeri Sembilan (S1) as discovered from cox1+16S haplotype network analysis. Overall haplotype and nucleotide diversities of the concatenated sequences were Hd = 0.89367 and Pi = 0.00282, respectively, with great genetic differentiation (FST = 0.63869) and low gene flow (Nm = 0.14). Population from Pahang (S7) presented the highest genetic diversity (Hd = 0.88889, Pi = 0.00215, Nh = 9), whereas Kedah population (S3) demonstrated the lowest diversity (Hd = 0.28421, Pi = 0.00026, Nh = 4). The concatenated sequences of cox1+16S showed genetic divergence ranging from 0.09% to 0.97%, whereas the genetic divergence for cox1 and 16S rRNA genes were 0.16% to 1.63% and 0.01% to 0.75% respectively. This study provides for the first-time insights on the intraspecific genetic diversity, phylogeographic pattern and ancestral haplotype of Rhinocypha fenestrella.
**Order of Authors:**	Noor Azrizal Abdul Wahid, Ph.D
	Noorhidayah Mamat, Ph.D
	Van Lun Low, Ph.D
	Norma-Rashid Yusoff, Ph.D
**Additional Information:**	
**Question**	**Response**
**Financial Disclosure**<br><br>Enter a financial disclosure statement that describes the sources of funding for the work included in this submission. Review the submission guidelines for detailed requirements. View published research articles from *PLOS ONE* for specific examples.<br><br>This statement is required for submission and **will appear in the published article** if	Author who received the funding award= Norma-Rashid Yusoff;<br>Grant numbers awarded to the author= 1;<br>The full name of funder= The Universiti Malaya Research Grant (UMRG) PG065 – 2015 and ST061-2022;<br>URL of each funder website= https://umhvl.um.edu.my/grants-and-projects-awarded;<br>Role of the funder: Conceptualization, Investigation, Methodology, Project administration, Supervision

Powered by Editorial Manager® and ProduXion Manager® from Aries Systems Corporation

the submission is accepted. Please make sure it is accurate.<br><br>**Unfunded studies**<br>Enter: *The author(s) received no specific funding for this work.*<br><br>**Funded studies**<br>Enter a statement with the following details:<br>• Initials of the authors who received each award<br>• Grant numbers awarded to each author<br>• The full name of each funder<br>• URL of each funder website<br>• Did the sponsors or funders play any role in the study design, data collection and analysis, decision to publish, or preparation of the manuscript?<br>• **NO** - Include this sentence at the end of your statement: *The funders had no role in study design, data collection and analysis, decision to publish, or preparation of the manuscript.*<br>• **YES** - Specify the role(s) played.<br><br>* typeset	
**Competing Interests**<br><br>Use the instructions below to enter a competing interest statement for this submission. On behalf of all authors, disclose any competing interests that could be perceived to bias this work—acknowledging all financial support and any other relevant financial or non-financial competing interests.<br><br>This statement is required for submission and **will appear in the published article** if the submission is accepted. Please make sure it is accurate and that any funding sources listed in your Funding Information later in the submission form are also declared in your Financial Disclosure statement.<br><br>View published research articles from *PLOS ONE* for specific examples.	The authors have declared that no competing interests exist

**NO authors have competing interests**<br><br>Enter: *The authors have declared that no competing interests exist.*<br><br>**Authors with competing interests**<br><br>Enter competing interest details beginning with this statement:<br><br>*I have read the journal's policy and the authors of this manuscript have the following competing interests: [insert competing interests here]*<br><br>* typeset	
**Ethics Statement**<br><br>Enter an ethics statement for this submission. This statement is required if the study involved:<br><br>• Human participants<br>• Human specimens or tissue<br>• Vertebrate animals or cephalopods<br>• Vertebrate embryos or tissues<br>• Field research<br><br>Write "N/A" if the submission does not require an ethics statement.<br><br>General guidance is provided below. Consult the submission guidelines for detailed instructions. **Make sure that all information entered here is included in the Methods section of the manuscript.**	N/A

Powered by Editorial Manager® and ProduXion Manager® from Aries Systems Corporation

**Format for specific study types**<br><br>**Human Subject Research (involving human participants and/or tissue)**<br>• Give the name of the institutional review board or ethics committee that approved the study<br>• Include the approval number and/or a statement indicating approval of this research<br>• Indicate the form of consent obtained (written/oral) or the reason that consent was not obtained (e.g. the data were analyzed anonymously)<br><br>**Animal Research (involving vertebrate animals, embryos or tissues)**<br>• Provide the name of the Institutional Animal Care and Use Committee (IACUC) or other relevant ethics board that reviewed the study protocol, and indicate whether they approved this research or granted a formal waiver of ethical approval<br>• Include an approval number if one was obtained<br>• If the study involved *non-human primates*, add *additional details* about animal welfare and steps taken to ameliorate suffering<br>• If anesthesia, euthanasia, or any kind of animal sacrifice is part of the study, include briefly which substances and/or methods were applied<br><br>**Field Research**<br><br>Include the following details if this study involves the collection of plant, animal, or other materials from a natural setting:<br>• Field permit number<br>• Name of the institution or relevant body that granted permission	
**Data Availability**<br><br>Authors are required to make all data underlying the findings described fully available, without restriction, and from the time of publication. PLOS allows rare exceptions to address legal and ethical concerns. See the PLOS Data Policy and FAQ for detailed information.	Yes - all data are fully available without restriction

Powered by Editorial Manager® and ProduXion Manager® from Aries Systems Corporation

A Data Availability Statement describing where the data can be found is required at submission. Your answers to this question constitute the Data Availability Statement and **will be published in the article**, if accepted.<br><br>**Important:** Stating 'data available on request from the author' is not sufficient. If your data are only available upon request, select 'No' for the first question and explain your exceptional situation in the text box.<br><br>Do the authors confirm that all data underlying the findings described in their manuscript are fully available without restriction?	
**Describe where the data may be found in full sentences. If you are copying our sample text, replace any instances of XXX with the appropriate details.**<br><br>• If the data are **held or will be held in a public repository**, include URLs, accession numbers or DOIs. If this information will only be available after acceptance, indicate this by ticking the box below. For example: *All XXX files are available from the XXX database (accession number(s) XXX, XXX.).*<br>• If the data are all contained **within the manuscript and/or Supporting Information files**, enter the following: *All relevant data are within the manuscript and its Supporting Information files.*<br>• If neither of these applies but you are able to provide **details of access elsewhere**, with or without limitations, please do so. For example:<br><br>*Data cannot be shared publicly because of [XXX]. Data are available from the XXX Institutional Data Access / Ethics Committee (contact via XXX) for researchers who meet the criteria for access to confidential data.*<br><br>*The data underlying the results presented in the study are available from (include the name of the third party*	All relevant data are within the manuscript and its Supporting Information files.

and contact information or URL).<br>• This text is appropriate if the data are owned by a third party and authors do not have permission to share the data.<br><br>* typeset	
Additional data availability information:	Tick here if the URLs/accession numbers/DOIs will be available only after acceptance of the manuscript for publication so that we can ensure their inclusion before publication.

Powered by Editorial Manager® and ProduXion Manager® from Aries Systems Corporation

Manuscript

Click here to access/download;Manuscript;2- Full Manuscript.docx

Research Paper

Genetic diversity and phylogeographic patterns of the peacock jewel-damselfly, *Rhinocypha fenestrella* (Odonata: Chlorocyphidae)

Mamat-Noorhidayah[1], Noor Azrizal-Wahid[2*], Van Lun Low[3], Norma-Rashid Yusoff[1]

[1] Institute of Biological Sciences, Faculty of Science, Universiti Malaya, Kuala Lumpur, Malaysia

[2] Department of Biology, Faculty of Science, Universiti Putra Malaysia, 43400 Serdang, Selangor, Malaysia

[3] Higher Institution Centre of Excellence (HICoE), Tropical Infectious Diseases Research & Education Centre (TIDREC), Universiti Malaya, 50603 Kuala Lumpur, Malaysia

*Corresponding author: noorazrizal@upm.edu.my

Abstract

The present study examined the genetic diversity and phylogeographic pattern of the peacock jewel-damselfly *Rhinocypha fenestrella* by sequencing the cytochrome C oxidase I (*cox1*) and *16S* rRNA gene regions from 147 individuals representing eight populations in Malaysia. A total of 26 and 10 unique haplotypes were revealed by the *cox1* and *16S* rRNA genes, respectively, and 32 haplotypes were recovered by the concatenated sequences of *cox1+16S*. Analyses indicated that haplotype AB2 was the most frequent and the most widespread haplotype in Malaysia, and the most recent common ancestor of the *R. fenestrella* may exist from the Negeri Sembilan (S1) as discovered from *cox1+16S* haplotype network analysis. Overall haplotype and nucleotide diversities of the concatenated sequences were H_d = 0.89367 and P_i = 0.00282, respectively, with great genetic differentiation (F_{ST} = 0.63869) and low gene flow (N_m = 0.14). Population from Pahang (S7) presented the highest genetic diversity (H_d =

0.88889, P_i = 0.00215, N_h = 9), whereas Kedah population (S3) demonstrated the lowest diversity (H_d = 0.28421, P_i = 0.00026, N_h = 4). The concatenated sequences of *cox1+16S* showed genetic divergence ranging from 0.09% to 0.97%, whereas the genetic divergence for *cox1* and *16S* rRNA genes were 0.16% to 1.63% and 0.01% to 0.75% respectively. This study provides for the first-time insights on the intraspecific genetic diversity, phylogeographic pattern and ancestral haplotype of *Rhinocypha fenestrella*.

Keywords: *cox1*, haplotype, Malaysia, population structure, *Rhinocypha fenestrella*, *16S*,

1. Introduction

Rhinocypha (Cowley 1937), from the family of Chlorocyphidae, is the most characteristic genus of dragonflies in tropical Asia. Rhinocyphae is of a very particular nature in their habitat [1] and certain species are more sensitive to habitat disturbance and are primarily found in undisturbed areas [2]. This characteristic makes them good "thermometers" of environment quality, thus good study subjects for phylogeography [3]. *Rhinocypha fenestrella* (Rambur 1842), syn, *Aristocypha fenestrella*, also known as a peacock jewel, is the most widespread species in the genus, and one of the most active species of the family, which occurs in Peninsular Malaysia, Thailand, Burma, Laos, Vietnam, and southern China [4]. It is one of several common mountain stream damselflies and is usually found in the primary forests [1].

Though Malaysia is known to be one of the three mega-biodiversity countries in Southeast Asia, the phylogeographic pattern of dragonflies has been relatively scarce, especially for this particular species, but little has been published. Several phylogeographic studies have been published concerning odonates [5,6,7], however, until now, no comparable research related to odonate species in Malaysia has been conducted. Instead, the species with a widespread distribution that includes dragonflies normally will have complexes of multiple

lineages or variations in the genetic diversity with the geographic region [8,9,10,11,12]. From a view of zoogeography, the Rhinocyphae are of substantial importance, for instance, in the Malay Archipelago, each large island, mostly has its own group of endemic species [1].

A phylogeographic analysis is a powerful method of obtaining insights into the historical processes that have shaped the species' temporal distribution and genetic variation [13]. Recent phylogeographic research on widely distributed odonatan species has indicated different responses to historical climatic changes in space and time [14,15]. With the advances in molecular techniques, mitochondrial DNA has been identified as an excellent genetic marker of gene flow in matrilineal inheritance [16] and it is the most widely used marker to study the molecular ecology in animal taxa [17,18].

Particularly, cytochrome C oxidase subunit I (*cox1*) and 16S ribosomal RNA (*16S*) are known to be reliable genetic markers and the commonly applied markers in Odonata [7,19,20,21]. In addition, these markers were also provided well-resolved and supported trees from species to family level [22,23]. Given the high resolution of mitochondria-encoded *cox1* and *16S* genes reported in odonates [24], this study attempts to characterize the intraspecific genetic diversity and population genetic structure of *R. fenestrella*, for the first time across its range in Malaysia.

2. Materials and methods

2.1 Animal material

A total of 147 *Rhinocypha fenestrella* individuals were collected from eight populations representing eight states in Peninsular Malaysia from the period of 2014 to 2015 with the permission of the Forestry Department Peninsular Malaysia (Permit Number: JH/100 Jld.7 (12)) (Table 1 and Fig 1). The identification of *R. fenestrella* was performed according to morphological descriptions and taxonomic keys as described by Orr and Hamalainen (2003)

[23], and through personal experience. Methods of sampling and preservation of Odonata were based on previously described standard methods [24].

2.2 DNA extraction and amplification

Genomic DNA was extracted from four to six legs of each fresh specimen using the i-genomic CTB DNA Extraction Mini Kit (iNtRON Biotechnology Inc., Seongnam, South Korea). The DNA amplifications of both *cox1* and *16S* genes were conducted using an Applied Biosystems Veriti 96-Well Thermal Cycler (Applied Biosystems Inc., Foster City, CA, USA) with the amplification protocol consisting of 300 sec at 94°C followed by 35 cycles of 50 sec at 94°C, 50 sec at 50°C and 50 sec at 72°C, and a final 7 min at 72°C. Primers for amplification of the *cox1* gene were 5'- GGT CAA CAA ATC ATA AAG ATA TTG G – 3' for forward primer [27] and 5'- GGA TGG CCA AAA AAT CAA AAT AAA TG –3' for reverse primer [28]. For the *16S* gene, ODO 12852 and ODO 13393 primer sets (forward primer, 5'- AGA AAC CGA CCT GGC TTA AA -3'; reverse primer, 5'- CGC CTG TTT ATC AAA AAC AT -3') were utilized [7]. Each PCR amplification was performed in a reaction mixture containing 50–100 ng of genomic DNA, 25 µl of NEXpro e-PCR 2X Master Mix (Genes Labs Inc., Gyeonggi-do, South Korea), and 10 pmol of each forward and reverse primer.

2.3 DNA purification, sequencing, and alignment

The amplified samples were then electrophoresed on 2% agarose gel pre-stained with SYBR Safe™ (Invitrogen Corp., Carlsbad, CA, U.S.A.), and the PCR products were sent outsource to a commercial company (Apical Scientific Sdn. Bhd. Selangor, Malaysia) for DNA sequencing in both forward and reverse directions. The samples were sequenced using the BigDyeH Terminator 3.1 Sequencing Kit.

All sequences were assembled and edited using Molecular Evolutionary Genetics Analysis (MEGA) software Version 11.0 [29] and BioEdit 7.2 [30] and preliminarily aligned using CLUSTALX [31]. The aligned sequences were then subjected to a BLAST search (www.ncbi.nlm.nih.gov/blast/) for species validation. The generated sequences that produce unique haplotypes in this study were deposited in the National Center for Biotechnology Information (NCBI) GenBank database under accession numbers KY678719–KY678744 for *cox1*, and KY678745–KY678754 for *16S* genes.

2.4 Genetic diversity and haplotype analyses

The aligned *cox1* and *16S* sequences comprised 614 bp and 534 bp, respectively, while the sequences of both *cox1* and *16S* were concatenated to yield a total length of 1148 bp. Molecular characteristics such as the number of haplotypes (N_h), haplotype diversity (H_d), nucleotide diversity (P_i), the number of segregating sites (S), and average number of sequence differences (K) were determined with the program DNASP® (DNA Sequence Polymorphism) [32]. DNASP® was also used to perform neutrality tests including Tajima's D [33] and Fu's F_s [34].

The F_{ST} and N_m pairwise values were determined to access genetic differentiation and gene flow among the *R. fenestrella* population and were calculated using DNASP®. The levels of genetic differentiation are defined as $F_{ST} > 0.25$ (great differentiation), $0.15 < F_{ST} < 0.25$ (moderate differentiation), and $F_{ST} < 0.15$ (negligible differentiation) according to the classification criteria by Wright (1978) [35]. The levels of gene flow are categorized as $N_m > 1$ (high gene flow), $0.25 < N_m < 0.99$ (intermediate gene flow), and $N_m < 0.25$ (low gene flow) [36]. Uncorrected (p) pairwise genetic distances were assessed using PAUP 4.0B10 to measure the genetic divergence of *R. fenestrella*.

Furthermore, to visualize the phylogeographic pattern between the populations, and to calculate the minimum number of mutational steps between the sequences, haplotype networks

were constructed using TCS 1.13© [[illegible]7] with a 95% parsimony criterion for both *cox1* and *16S* respectively, and concatenated *cox1* + *16S* genes sequences.

3. Results

3.1 Genetic diversity of *Rhinocypha fenestrella*

The final aligned sequence fragment lengths were 614 bp, 534 bp, and 1148 bp for *cox1*, *16S*, and concatenated *cox1+16S*, respectively. The haplotype diversity (H_d) in a population for the *cox1* gene ranged from 0.1947[illegible] the Kedah population to 0.88889 in the Pahang population. Nonetheless, for the *16S* gene, it ranged from 0.000[illegible]und in three populations (Johor, Perak, and Selangor) to 0.28421 in the Kelantan population. In concatenated *cox1+16S* sequence analyses, the overall value of haplotype diversity was 0.89367, and nucleotide diversity (P_i) was 0.00282.

Kedah population showed the lowest diversity for both nucleotide and haplotype diversities (H_d = 0.28421, P_i = 0.00026), while the highest haplotype diversity was shown by Pahang (H_d = 0.88889), and the highest nucleotide diversity was presented by the Kelantan population (P_i = 0.00228) (Table 2). In total average data estimates, the *cox1* gene revealed higher for both haplotype and nucleotide diversities (H_d = 0.88463, $P_{i\,=}$ 0.00505) than did the *16S* gene (H_d = 0.1292, $P_{i\,=}$ 0.00134).

3.2 Haplotype variation and distribution

A set of aligned sequences of 147 taxa of the *cox1* revealed 26 haplotypes (A1 – A26). For *cox1*, the most prevalent haplotype was A2 (*n* = 40) and was found ~~to exist~~ in five populations. The second most-frequent haplotypes were A1, A4, and A14 (*n* = 15) while the least prevalent haplotypes presented as singleton were A3, A5, A7 – A11, A18 – A20, A23, and A25. Notably,

based on the star-like pattern of the haplotype network (Fig 2), the haplotypes A2 – A26 from the *cox1* gene were considered to be originated from haplotype A1, ~~as their common ancestor~~.

For the *16S* gene, the sequences revealed 10 haplotypes (B1 – B10). The most prevalent haplotype was B1 (*n* = 140) while all other haplotypes except B8 (*n* = 2), appeared as singleton haplotypes. Haplotype B1 was ~~also the most widespread and can be~~ found in all populations. The haplotype network of *16S* gene showed a star-like pattern suggesting haplotype B1 as a common ancestor ~~while other haplotypes (B2 – B10) originated from haplotype~~ B1 (Fig 3). ~~Nevertheless~~, a median-joining network showed no obvious geographical pattern in haplotype distribution.

The concatenated *cox1+16S* sequences revealed 32 haplotypes (AB1 – AB32). Haplotype AB2 was the most frequent and the most widespread haplotype based on its prevalence in Malaysia, while there were 18 singleton haplotypes (AB3, AB4, AB6, AB8 – AB12, AB15, AB18, AB20, AB21, AB 23 – AB26, AB29, and AB31 (Table 4). Furthermore, the Kelantan population presented as the most diverse population that had the highest number of haplotypes (N_h = 30) while the least number of haplotypes was presented in the Selangor population (N_h = 11). Haplotype AB1 appeared to be as a common ancestor ~~to~~ the other haplotypes as indicated by the star-like pattern of concatenated *cox1+16S* haplotype network (Fig 4). Consequently, in terms of location-based this study revealed that a recent common ancestor of the *R. fenestrella* species in Malaysia existed from the Negeri Sembilan population that constituted the most numbers of haplotype AB1.

3.3 Haplotype genetic divergence

Uncorrected genetic divergence between *R. fenestrella* haplotypes for *cox1* ranged from 0.16% – 1.63% with the highest value showed pairwise between haplotypes A5 with A15, A17, A19, and between haplotypes A7 with A15, A17, A19 (Appendix A – see supplementary file). On

the other hand, the genetic divergence of *16S* haplotypes ranged from 0.01% – 0.75% with the highest value presented pairwise between haplotypes B5 and B7 (Appendix B – see supplementary file). For the concatenated *cox1+16S*, the genetic divergence ranged from 0.09% to 0.97% (Table 5).

In concatenated *cox1+16S* sequences, the neutrality test of Fu's F_s showed negative values in all populations with an exception for Selangor. Additionally, Tajima's D tests for all populations were indicated by negative values and were not statistically significant. The overall F_{ST} was 0.63869 and N_m was 0.14, suggesting that the genetic differentiation was great while there was a low gene flow among the populations of *R. fenestrella* in Malaysia. Based on Table 3, the highest F_{ST} value (0.010) appeared between Perak and Selangor populations while the lowest value (0.001) was between the populations of Negeri Sembilan and Kedah, Negeri Sembilan and Perak, Kedah, and Perak, Kedah and Pahang, and Kedah and Selangor.

4. Discussion

4.1 Genetic diversity of Malaysian *Rhinocypha fenestrella*

Throughout the last five decades, the understanding of the ecology and evolution of odonates has increased dramatically while recent advances in molecular techniques have inspired several phylogeographical studies of odonat[illegible]y using genetic data [24,38,39]. As is known, most of the odonates are extremely mobile and have varying levels of dispersal abilities [40] that could influence the genetic diversity and phylogeographic structures of the populations. A less-mobile species may be expected to show some evidence of haplotype clustering according to geographic region [41]. Therefore, in this study, we defined the intraspecific genetic diversity, phylogeographical patterns, and mitochondrial variations of the *Rhinocypha fenestrella* using *cox1* and *16S* genes for the first time across eight populations within Peninsular Malaysia.

The obtained sequences for *cox1* and *16S* genes from eight populations ranged from 1.5 – 4.6% of variability among all studied individuals reflected in 28 variable nucleotide sites in 614 sequenced base pairs from *cox1* gene, and eight variable nucleotides in 534 base pairs from *16S* gene. The base composition of both *cox1* and *16S* gene sequences showed a significant A+T nucleotide bias which is consistent with insect mitochondrial genes [33,42,43].

Based on the present study, overall genetic diversity (H_d = 0.52909; P_i = 0.00424) of Malaysian *R. fenestrella* based on the concatenated *cox1+16S* sequences was slightly lower if compared to the study that did on the damselfly *Matrona basilaris* populations from mainland China [44]. The study reported higher values of haplotype diversity (H_d = 0.975) and nucleotide diversity (P_i = 0.00487) by using *cox1* sequences. Nevertheless, the genetic diversity of *R. fenestrella* in Malaysia can be considered as high when compared to other damselflies species from the same sub-order *Psolodesmus mandarinus* from Taiwan that reported haplotype diversity as 0.43 and nucleotide diversity as 0.0017 based on *cox1+16S* sequences [45].

4.2 Haplotype variation and distribution of *Rhinocypha fenestrella*

In this study, the number of haplotypes detected was considered low because only ten haplotypes were revealed by *cox1* and eight haplotypes by *16S* with the presence of several singleton haplotypes that were restricted to particular areas. The low numbers of haplotypes may indicate a low degree of *cox1* and *16S* gene polymorphisms in *R. fenestrella* samples in Peninsular Malaysia. However, this study demonstrated high numbers of haplotype-based on concatenated data that produced 32 haplotype variations. As compared to other studies, Lin et al. (2012) [45] reported only 14 haplotypes variation in the damselfly population of *Psolodesmus mandarinus* from Taiwan while only four haplotypes were discovered in the dragonfly population from Scandinavia and Finland [46].

The haplotype AB2 appeared as the most dominant haplotype, while the limited geographical distribution of some of the haplotypes including those found as singleton suggests the existence of genetic differentiation within the populations. In this study, haplotype AB1 was suggested as the common ancestor due to star-like radiation in the haplotype network pattern. This haplotype probably had eventually evolved over time into the numerous haplotypes (AB2–AB15) in order to adapt to the habitat and demographic changes and consequently distributed across states in Peninsular Malaysia. Likewise, according to location-based, the most recent common ancestor for *R. fenestrella* in Malaysia may be derived from Negeri Sembilan as this state is constituted by high frequencies of the common ancestor AB1 haplotypes.

The median-joining network also revealed a close relationship among haplotypes, suggesting that *R. fenestrella* populations in Malaysia shared a recent history without long-term genetic isolation. Nevertheless, the ancestral haplotype AB1 of concatenated *cox1+16S* dataset was having a limited distribution with restricted geographic division. This indicates that geographic barriers and climatic factors could also have little influence on the dispersion of this damselflies into different habitats in Malaysia besides their less ability to disperse due to weak flyers.

4.3 Genetic distance and differentiation

In our dataset, the highest genetic distance value based on *cox1* gene was 0.16%, while *16S* gene was 0.75%. The data shows a higher genetic distance among Malaysian *R. fenestrella* as compared to the populations of *Rhinocypha taiwan*[illegible]*hinocypha ueno*[illegible]nd *Rhinocypha drusill*[illegible]om China where the highest genetic distances based on *cox1* gene were 0.0%, 0.0% and 0.5%, respectively [47]. However, the genetic distance of Malaysian *R. fenestrall*[illegible] was relatively lower if compared to other dragonfly populations of *Trithemis stictic*[illegible]n

Namibia [9] and *Nannophya pygma*[illegible] Malaysia [48] that presented the genetic distance of *cox1* gene ranging from 0.00% - 9.00% and 0.00% - 12.0%, respectively[illegible]

Zygopterans are known as weaker fliers by [illegible]any species not diffusin[illegible]utside several kilometres [49,50]. *Rhinocypha fenestrella* is known as a weak flyer and is unlikely to migrate over large-scale region[illegible]hich likely contributes to its overall high genetic differentiation to happen in and between the population[illegible]herefore, in the present study, it is somehow not surprising that genetic differentiation happene[illegible] the species *R. fenestrella* in which their populations were separated up to more than 500 km. In contrast, the migratory behaviour of odonate could homogenize genetic differentiation among populations by the exchange of individuals and genes among populations. For example, low genetic differentiation has been observed in the population of migratory dragonflies *Libellula quadrimaculat*[illegible]3] and *Pantala flavesen*[illegible]1] respectively where the[illegible]e known as strong fliers that the damselflies.

Natura[illegible]spersal ability and long-distance migration are the most important factors contributing to a high level of gene flow and consequent slowing or limitatio[illegible] geographic differentiation [52,53]. A less-mobile species may be expected to show some evidence of haplotype clustering according to geographic region due to less ability to disperse and therefore resulted in genetic isolation by distance. In this study, calculated F_{st} value shows high genetic differentiation occurred in the populatio[illegible]f *R. fenestrella* in Malaysia. This high value is consequently supported by the low rate of gene flow found amongst the *R. fenestrella* populations in Malaysia as well as between the different regions[illegible]

When all eight populations were regarded as a whole, Tajima's *D* statistic was statistically negative and not significan[illegible]he results showed that these populations were in a stable state with no demographic expansion and no recent bottleneck. Whereas, in the Selangor population, Fu's F_S statistic values were negative with *p*-values being significant ($p < 0.02$) which shows this population had experienced a recent population expansion. Our results

suggested that *R. fenestrella* population in Selangor could successfully colonize and adapt to new habitats and were able to disperse randomly and exchange genes with local populations. Maintaining genetic diversity is noteworthy as it related to population viability [54,55,56] and also to the transformative potential of a species to react to the environmental changes [57,58].

Overall, this study found strong evidence for intraspecific patterns of molecular variation among populations of *R. fenestrella* in Malaysia though the weak dispersal abilities of the species. The study also revealed high genetic differentiation ~~while~~ a low rate of gene flow among the geographically difference populations of Malaysian *R. fenestrella*. Moreover, a high haplotype number was observed in *R. fenestrella* population but with no genetic isolation by populations. Although many phylogeographical mechanisms have been proposed using damselflies as model organisms, but more detailed sampling and a larger variety of ecological investigations are required to promote understanding of damselfly and advance their evolution and phylogeography. Additional sequence data from this particular species gene from another continent (Thailand, Burma, Laos, Vietnam, and southern China), which is considered as a gap of knowledge, may prove useful in detecting phylogenetic relationships, and phylogeography patterns, and revealing common ancestors of these populations within species between the continents.

Acknowledgements

This study was supported by a research grant from the University of Malaya, PG065 – 2015 and ST061-2022. The authors would like to thank Mr. Mohaiyidin Mohamed and Mr. Mohd Fauzi Abd Hamid for their assistance in the fieldwork.

References

1. Lieftinck MA. Two interesting new insular *Rhinocypha* from Malaysia (Odon.). Tijdschrift Voor Entomologie. 1947; 88: 215–224.
2. Jumawan K, Medina M, Villanueva RJ. Annotated list of Odonata from Mainit Hot Spring Protected Landscape, Compostella Valley, Mindanao Island, Philippines. Philippine Journal of Systematic Biology. 2012; 5(1): 14–18.
3. Córdoba–Aguilar A. Introduction. In: Córdoba–Aguilar A, editor. Dragonflies and damselflies: study models in ecological and evolutionary research. Oxford (UK): Oxford University Press; 2008. p. 1–3.
4. Sharma G. *Rhinocypha fenestrella*. The IUCN Red List of Threatened Species. 2010.
5. Kahilainen A, Puurtinen M, Kotiaho JS. Conservation implications of species–genetic diversity correlations. Global Ecology and Conservation. 2014; 2: 315–323.
6. Monroe E, Britten B. Conservation in Hine's sight: the conservation genetics of the federally endangered Hine's emerald dragonfly, Somatochlora hineana [illegible]ournal of Insect Conservation. 2014; 18: 353–363.
7. Dijkstra KDB, Kalkman VJ, Dow RA, Stokvis FR, Tol JV. Redefining the damselfly families: the first comprehensive molecular phylogeny of Zygoptera (Odonata). Systematic Entomology. 2014; 39(1): 68–96.
8. Angulo A, Icochea J. Cryptic species complexes, widespread species and conservation: lessons from Amazonian frogs of the *Leptodactylus marmoratus* group (Anura: Leptodactylidae). Systematics and Biodiversity. 2010; 8: 357–370.
9. Damm S, Schierwater B, Hadrys H. An integrative approach to species discovery in odonates: from character-based DNA barcoding to ecology. Molecular Ecology. 2010; 19: 3881–3893.
10. Low VL, Takaoka H, Adler PH, Ya'cob Z, Norma-Rashid Y, Chen CD, Sofian-Azirun M. A multi-locus approach resolves the phylogenetic relationships of the *Simulium asakoae*

and *Simulium ceylonicum* species groups (Diptera: Simuliidae) in Malaysia: evidence for distinct evolutionary lineages. Medical and Veterinary Entomology. 2015; 29: 330–337.

11. Low VL, Tay ST, Kho KL, Koh FX, Tan TK, Lim YAL, Panchadcharam C, Ong BL, Norma-Rashid Y, Sofian-Azirun M. Molecular characterisation of the tick *Rhipicephalus microplus* in Malaysia: new insights into the cryptic diversity and distinct genetic assemblages throughout the world. Parasites and Vectors. 2015; 8: 341.
12. Low VL, Takaoka H, Pramual P, Adler PH, Ya'cob Z, Huang YT, Pham XD, Ramli R, Chen CD, Wannaket A, Sofian-Azirun M. Delineating taxonomic boundaries in the largest species complex of black flies (Simuliidae) in the Oriental Region. Scientific Reports. 2016; 6: 20346.
13. Avise JC. *Phylogeography*: The History and Formation of Species. Harvard University Press, Cambridge. 2000.
14. Kohli M, Letsch H, Greve C, Béthoux O, Deregnaucourt I, Liu S, Zhou X, Donath A, Mayer C, Podsiadlowski L, Gunkel S, Machida R, Niehuis O, Rust J, Wappler T, Yu X, Misof B, Ware J. Evolutionary history and divergence times of Odonata (dragonflies and damselflies) revealed through transcriptomics. iScience. 2021; 24(11): 103324.
15. Cancellario T, Miranda R, Baquero E, Fontaneto D, Martínez A, Mammola S. Climate change will redefine taxonomic, functional, and phylogenetic diversity of Odonata in space and time. npj Biodiversity. 2022; 1(1): 1–14.
16. Jisha KEK, Sebastian CD. Genetic variation and phylogeny assessment of *Aciagrion Occidentale* (Odonata: Coenagrionidae) using mitochondrial cytochrome oxidase subunit I gene. International Journal of Science and Research. 2013; 4(4): 1121–1123.
17. Norris DE. Genetic markers for study of the anopheline vectors of human malaria. Int Journal of Parasitology. 2002; 32: 1607–1615.

18. Pramual P, Kuvangkadilok C, Baimai V, Walton C. Phylogeography of the black fly *Simulium tani* (Diptera: Simuliidae) from Thailand as inferred from mtDNA sequences. Molecular Ecology. 2005; 14: 3989–4001.
19. Artiss T, Schultz TR, Polhemus DA, Simon C. Molecular phylogenetic analysis of dragonfly genera *Libellula*, *Ladona* and *Plathemis* (Odonata: Libelluinidae) based on mitochondrial cytochrome oxidase I and 16S rRNA sequence data. Molecular Phylogenetics and Evolution. 2001; 18: 348–361.
20. Yong HS, Lim P-E, Tan J, Ng YF, Eamsobhana P, Suana IW. Molecular phylogeny of *Orthetrum* dragonflies reveals cryptic species of *Orthetrum pruinosum*. Scientific Reports. 2014; 4: 5553.
21. Kim MJ, Jung KS, Park, NS, Wan X, Kim KG, Jun J, Jun J, Yoon TJ, Bae YJ, Lee SM, Kim I. Molecular phylogeny of the higher taxa of Odonata (Insecta) inferred from COI, 16S rRNA, 28S rRNA, and EF1-α sequences. Entomological Research. 2014; 44(2): 65–79.
22. Hasegawa E, Kasuya E. Phylogenetic analysis of the insect order Odonata using 28S and 16S rDNA sequences: a comparison between data sets with different evolutionary rates. Entomological Science. 2006; 9(1): 55–66.
23. Ballare EF, Ware JL. Dragons fly, biologists classify: an overview of molecular odonate studies, and our evolutionary understanding of dragonfly and damselfly (Insecta: Odonata) behavior. International Journal of Odonatology. 2011; 14: 137–147.
24. Ožana S, Dolný A, Páne T. Nuclear copies of mitochondrial DNA as a potential problem for phylogenetic and population genetic studies of Odonata. Systematic Entomology. 2022; 47(4): 591–602.
25. Orr AG, Hamalainen M. *A Guide to the Dragonflies of Borneo: their Identification and Biology*. Natural History Publications, Borneo. 2003.

26. Orr D. The constitution of nature. Conservation Biology. 2003; 17: 1478–1484.
27. Folmer O, Black M, Hoeh W, Lutz R, Vrijenhoek R. DNA primers for amplification of mitochondrial cytochrome C oxidase subunit I from diverse metazoan invertebrates. Molecular Marine Biology and Biotechnology. 1994; 3(5): 294–299.
28. Barrett RDH, Hebert PDN. Identifying spiders through DNA barcodes. Canadian Journal of Zoology. 2005; 83(3): 481–491.
29. Kumar S, Stecher G, Tamura K. MEGA7: Molecular Evolutionary Genetics Analysis Version 7.0 for Bigger Datasets. Molecular Biology and Evolution. 2016; 33(7): 1870–1874.
30. Hall TA. BioEdit: a user-friendly biological sequence alignment editor and analysis program for Windows 95/98/NT. Nucleic Acids Symposium Series. 1999; 41(2): 95–98.
31. Thompson JD, Gibson TJ, Plewniak F, Jeanmougin F, Higgins DG. The ClustalX Windows interface: flexible strategies for multiple sequence alignment aided by quality analysis tools. Nucleic Acids Research. 1997; 24: 4876–4882.
32. Librado P, Rozas J. DNASP v5: A software for comprehensive analysis of DNA polymorphism data. Bioinformatics. 2009; 25: 1451–1452.
33. Tajima F. Statistical method for testing the neutral mutation hypothesis by DNA polymorphism. Genetics. 1989; 123: 585–595.
34. Fu YX. Statistical tests of neutrality of mutations against population growth, hitchhiking and background selection. Genetics. 1997; 147: 915–925.
35. Wright S. *Evolution and Genetics of Populations*. 1978. University of Chicago, Chicago.
36. Slatkin M. Estimating levels of gene flow in natural populations. Genetics. 1981; 99: 323–335.
37. Clement M, Posada D, Crandall KA. TCS: a computer program to estimate gene genealogies. Molecular Ecology. 2000; 9(10): 1657–1659.

38. Artiss T. Phylogeography of a facultatively migratory dragonfly, *Libellula quadrimaculata* (Odonata: Anisoptera). Hydrobiologia. 2004; 515: 225–234.

39. Simonsen T, Olsen K, Djernaes M. The African-Iberian connection in Odonata: mtDNA and ncDNA based phylogeography of Aeshna cyane[illegible]Müller, 1764) (Odonata: Aeshnidae) in Western Palaearctic. Arthropod Systematics and Phylogeny. 2020; 78(2): 309–320.

40. Rundle SD, Bilton DT, Foggo A. Body Size: The Structure and Function of Aquatic Ecosystems. In: Hildrew AG, Raffaelli DG, Edmonds-Brown R. editors. By wind, wings or water: body size, dispersal and range size in aquatic invertebrates. Cambridge (UK): Cambridge University Press; 2007. p. 186–209.

41. Shannon J, McCauley SJ, Davis CJ, Werner EE, Robeson MS. Dispersal, niche breadth and population extinction: colonization ratios predict range size in North American dragonflies. Journal of Animal Ecology. 2014; 83: 858–865.

42. Frati F, Simon C, Sullivan J, Swofford DL. Evolution of the mitochondrial cytochrome oxidase II gene in Collembola. Journal of Molecular Evolution. 1997; 44(2): 145–158.

43. Chippindale PT, Whitmore DH, Davé VK, Valencia TG, Robinson JV. Effective procedures for the extraction, amplification and sequencing of Odonate DNA. Odonatologica. 1998; 27: 415–424.

44. Xue J, Zhang H, Ning X, Bu W, Yu X. Evolutionary history of a beautiful damselfly, Matrona basilari[illegible]evealed by phylogeographic analyses: the first study of an odonate species in mainland China. Heredity (Edinb). 2019; 122(5): 570–581.

45. Lin SC, Chen YF, Shieh SH, Yang PS. Patterns of mitochondrial and wing morphological differentiation in Taiwanese populations of *Psolodesmus mandarinus* McLachlan (Zygoptera: Calopterygidae). Odonatologica. 2012; 41(2): 109–121.

46. Kohli MK, Sahlén G, Kuhn WR, Ware JL. Extremely low genetic diversity in a circumpolar dragonfly species, *Somatochlora sahlbergi* (Insecta: Odonata: Anisoptera). Scientific Reports. 2018; 8(1): 15114.

47. Wang LJ, Cherng JJ, Chang YJ, Jiang JL. Description of *Rhinocypha taiwana* sp. nov. from Taiwan, with a preliminary molecular phylogenetic analysis of the *Rhinocypha drusilla*-group (Odonata: Chlorocyphidae). International Journal of Odonatology. 2013; 16: 93–107.

48. Low VL, Sofian-Azirun M, Norma-Rashid Y. Playing hide-and-seek with the tiny dragonfly: DNA barcoding discriminates multiple lineages of *Nannophya pygmaea* in Asia. Journal of Insect Conservation. 2016; 20: 339–343.

49. Purse BV, Hopkins GW, Day KJ, Thompson DJ. Dispersal characteristics and management of a rare damselfly. Journal of Applied Ecology. 2003; 40: 716–728.

50. Watts PC, Rouquette JR, Saccheri IJ, Kemp SJ, Thompson DJ. Molecular and ecological evidence for small-scale isolation by distance in an endangered damselfly, *Coenagrion mercuriale*. Molecular Ecology. 2004; 13: 2931–2945.

51. Low VL, Norma-Rashid Y, Yusoff A, Vinnie-Siow WY, Prakash BK, Tan TK, Noorhidayah M, Chen CD, Sofian-Azirun M. Pleistocene demographic expansion and high gene flow in the Globe Skimmer dragonfly *Pantala flavescens* Fabricius (Odonata: Libellulidae) in Peninsular Malaysia. Zoologischer Anzeiger. 2017; 266: 23–27.

52. Rowe RJ. Conservation of Odonata in the South Pacific and Australasia. International Journal of Odonatology. 2004; 7(2): 139–147.

53. Ware J, Kohli MK, Mendoza CM, Troast D, Jinguji H, Hobson KA, Sahlén G, Anderson RC, Suhling F. Evidence for widespread gene flow and migration in the Globe Skimmer dragonfly *Pantala flavescens*. International Journal of Odonatology. 2022; 25: 43–55.

54. Saccheri IJ, Kuussaari M, Kankare M, Vikman P, Fortelius W, Hanski I. Inbreeding and extinction in a butterfly metapopulation. Nature. 1998; 392: 491–494.
55. Madsen T, Shine R, Olsson M, Wittzell H. Conservation biology – restoration of an inbred adder population. Nature. 1999; 402: 34–35.
56. Spielman D, Brook BW, Briscoe BW, Frankham R. Does inbreeding and a loss of genetic diversity decrease disease resistance?. Conservation Genetics. 2004; 5: 439–448.
57. Frankham R, Ballou JD, Briscoe DA. *Introduction to Conservation Genetics*. Cambridge University Press, Cambridge. 2002.
58. Reed DH, Frankham R. Fitness is correlated with genetic diversity. Conservation Biology. 2003; 17: 1–9.

List of figures:

Fig 1. Map of sampling sites and haplotype distribution (AB1–AB15) of concatenated *cox1+16S* sequences for *Rhinocypha fenestrella* from eight sampling locations representing eight states of Peninsular Malaysia.

Fig 2. Median joining haplotype network of *Rhinocypha fenestrella* of *cox1* sequences isolated from eight different states in Peninsular Malaysia. Each circle represents a haplotype variation, and the size of a circle is proportional to the number of sequences assigned to that haplotype. Circles of the same colour represent haplotypes from the same population. A small black square represents median vectors.

Fig 3. Median joining haplotype network of *Rhinocypha fenestrella* of *16S* sequences isolated from eight different states in Peninsular Malaysia. Each circle represents a haplotype variation, and the size of a circle is proportional to the number of sequences assigned to that haplotype. Circles of the same colour represent haplotypes from the same population. A small black square represents median vectors.

Fig 4. Median joining haplotype network of *Rhinocypha fenestrella* of concatenated *cox1+16S* sequences isolated from eight different states in Peninsular Malaysia. Each circle represents a haplotype variation, and the size of a circle is proportional to the number of sequences assigned to that haplotype. Circles of the same colour represent haplotypes from the same population. A small black square represents median vectors.

Supporting information

S1 Table A. Percentage (%) of uncorrected “p” distance matrix among the 26 representative *cox1* haplotypes of *Rhinocypha fenestrella* in Malaysia.

S2 Table B. Percentage (%) of uncorrected “p” distance matrix among the 10 representative *16S rRNA* haplotypes of *Rhinocypha fenestrella* in Malaysia.

Author Contributions

Conceptualization: Norma-Rashid Yusoff, Mamat-Noorhidayah

Data curation: Mamat-Noorhidayah

Formal analysis: Mamat-Noorhidayah, Noor Azrizal-Wahid, Van Lun Low,

Funding acquisition: Norma-Rashid Yusoff

Investigation: Mamat-Noorhidayah, Norma-Rashid Yusoff

Methodology: Mamat-Noorhidayah, Norma-Rashid Yusoff

Project administration: Norma-Rashid Yusoff

Supervision: Norma-Rashid Yusoff

Visualization: Mamat-Noorhidayah, Noor Azrizal-Wahid, Van Lun Low

Writing – original draft: Mamat-Noorhidayah, Noor Azrizal-Wahid

Writing – review & editing: Noor Azrizal-Wahid, Van Lun Low

Figure 1

Click here to access/download;Figure;Figure 1 - Sampling Map & Haplotype Distribution.jpg

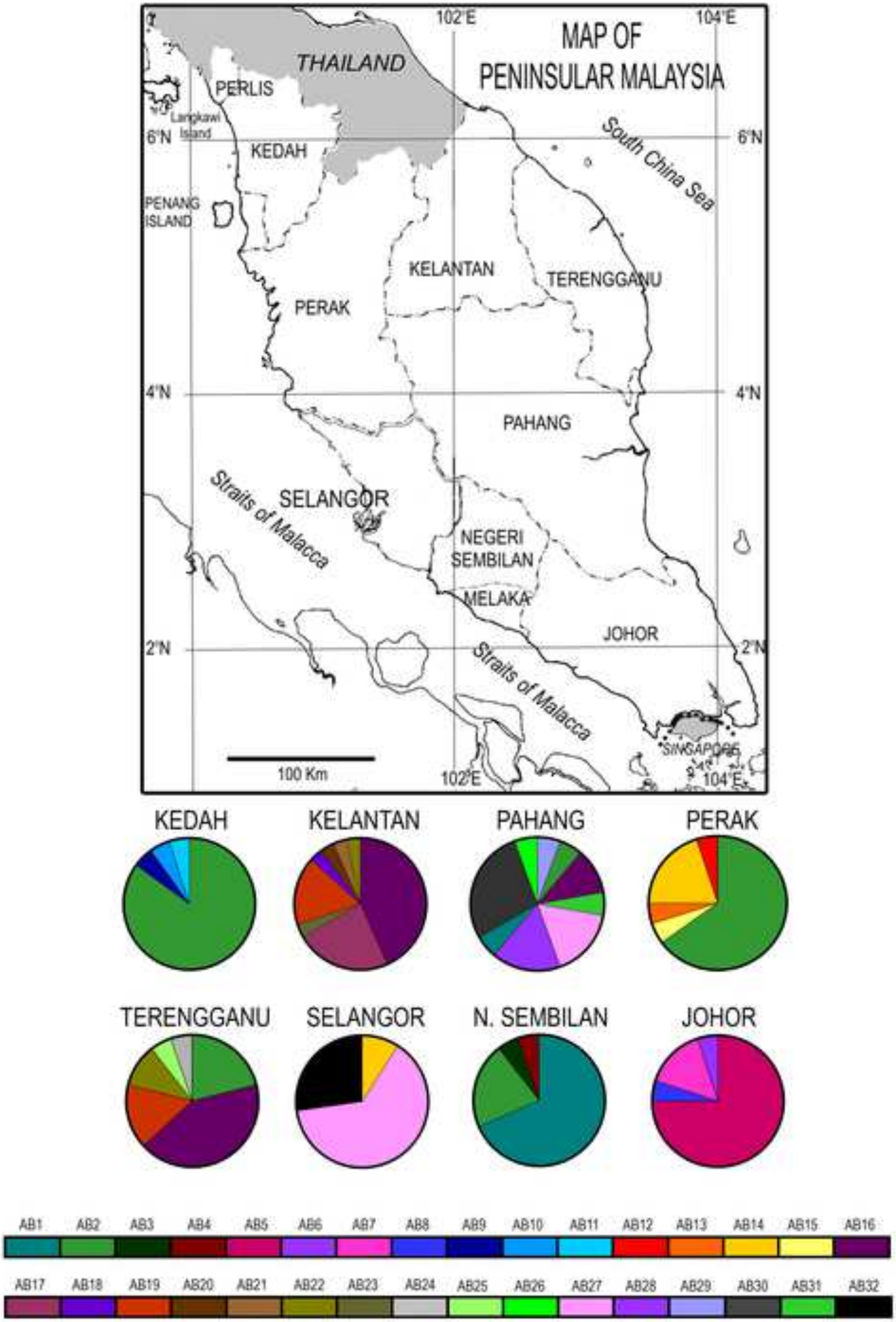

Figure 2

Click here to access/download;Figure;Figure 2 - cox1 Haplotype Network.jpg

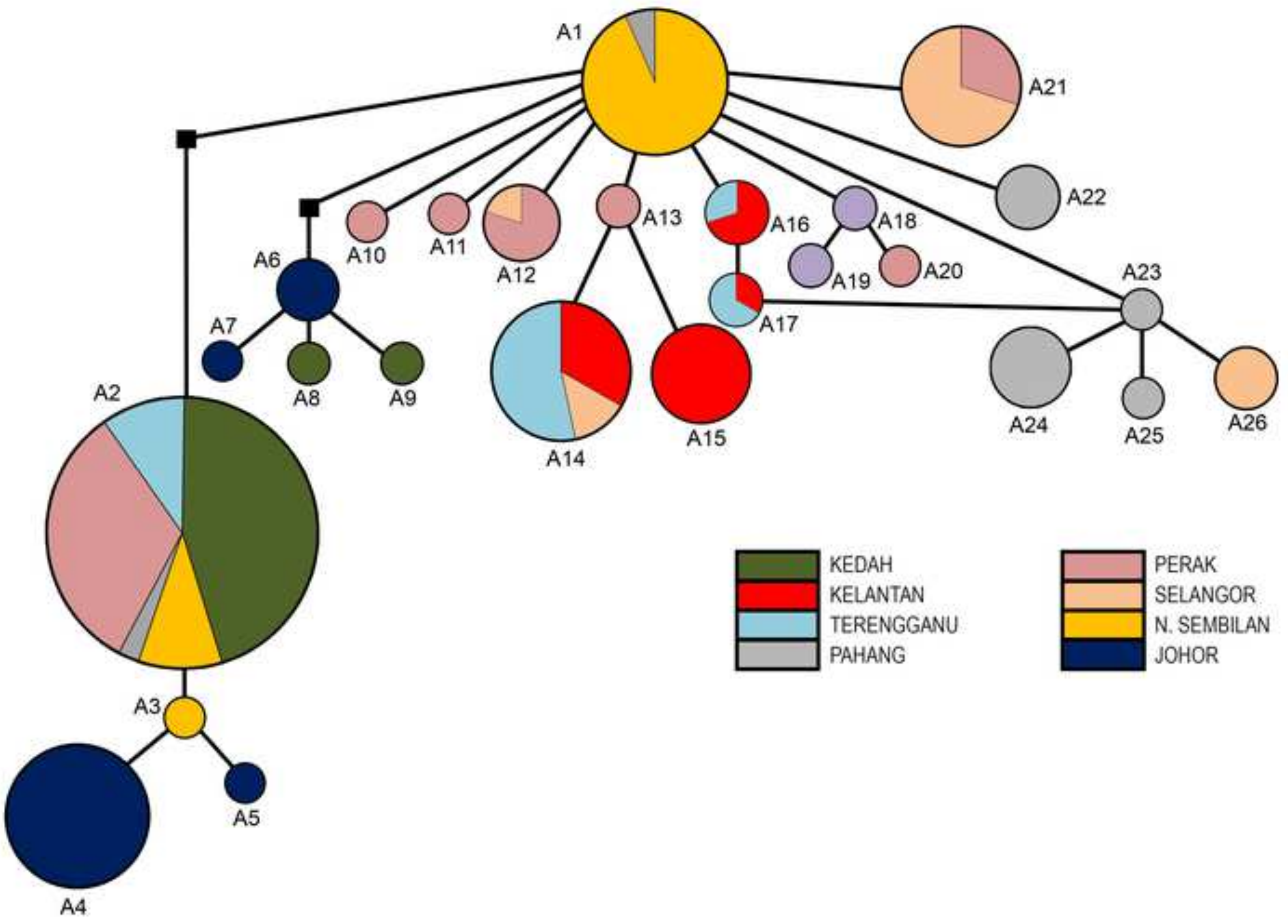

Figure 3

Click here to access/download;Figure;Figure 3-16S Haplotype Network.jpg

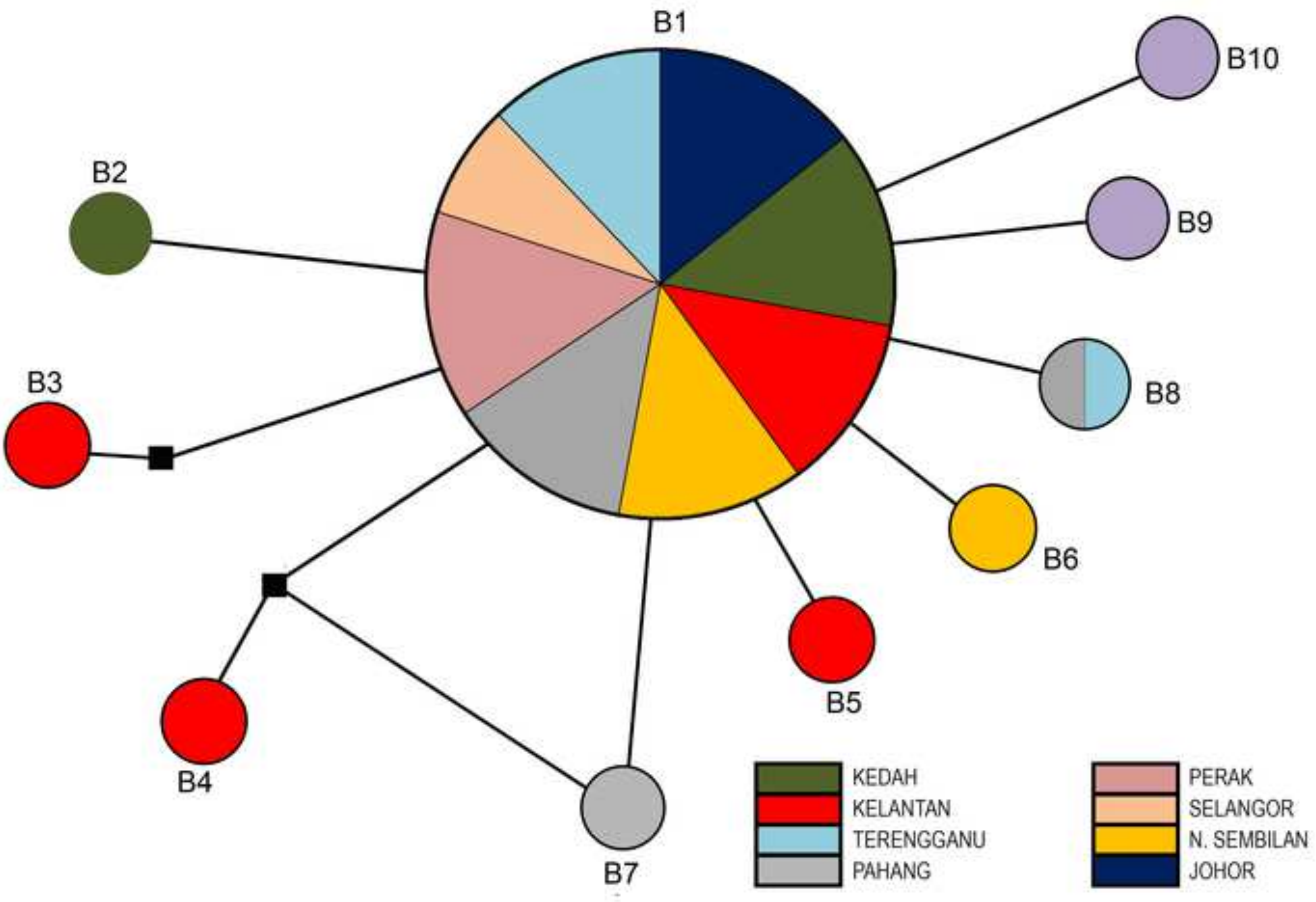

Figure 4

Click here to access/download;Figure;Figure 4 - Concatenated Haplotype Network.jpg

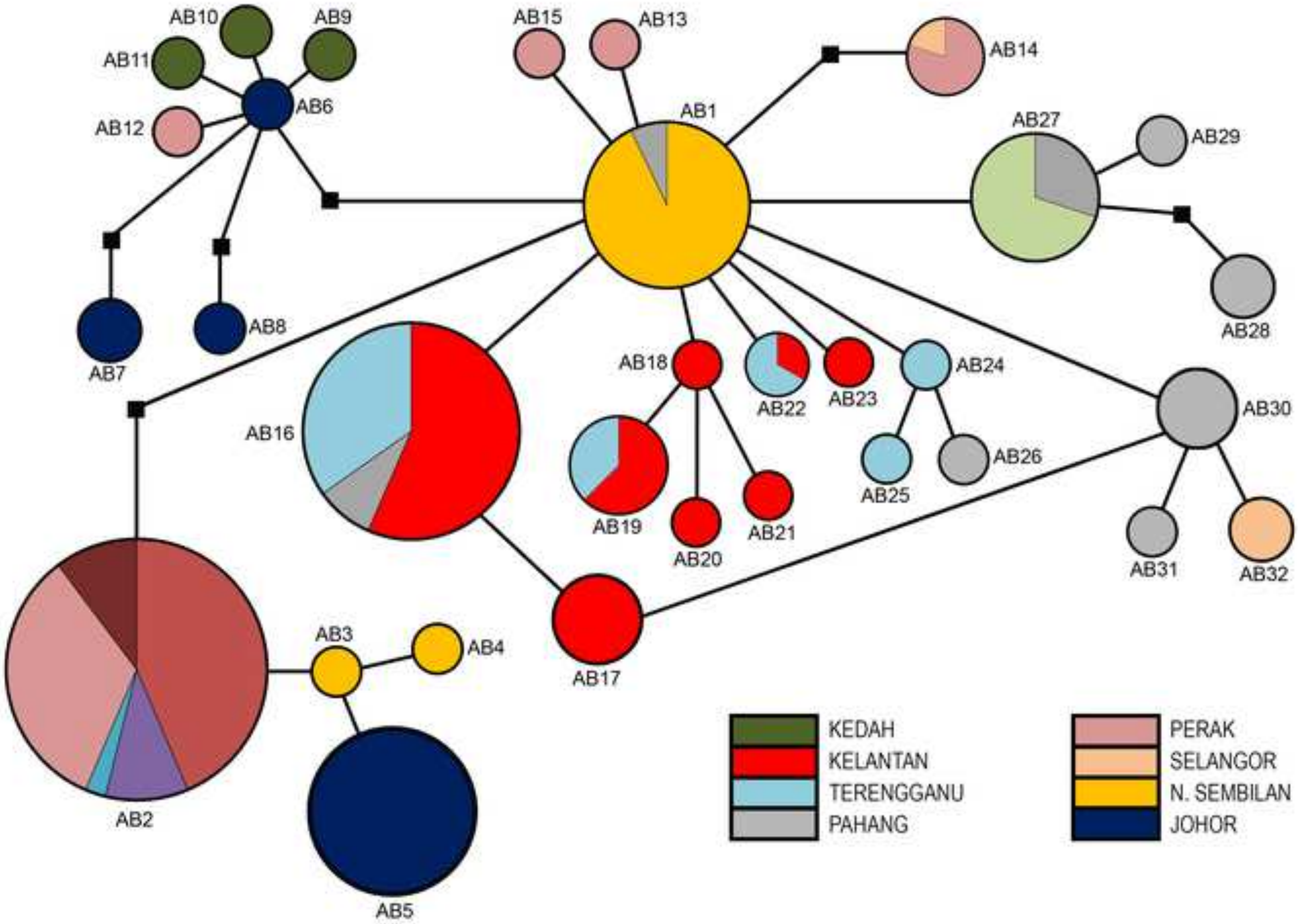

Click here to access/download
Supporting Information
7- Supplementary Data.docx

Click here to access/download
Supporting Information
5- List of Tables.docx

List of Figures

Click here to access/download
Supporting Information
4- List of Figures.docx

Graphical Abstract

Click here to access/download
Supporting Information
Graphical Abstract.jpg

Highlights

Click here to access/download
Supporting Information
3- Highlights.docx
