# Supplementary material for: Genetic diversity and phylogeographic patterns of the peacock jewel-damselfly, Rhinocypha fenestrella (Rambur, 1842)
Source: PLoS One. 2024 Apr 5;19(4):e0301392. doi: 10.1371/journal.pone.0301392 (PMC10997100; doi:10.1371/journal.pone.0301392)
Supplement: Attachment — Submitted filename: PONE-D-23-28970_R1-Review.pdf [file pone.0301392.s007.pdf]

PLOS ONE

Genetic diversity and phylogeographic patterns of the peacock jewel-damselfly, Rhinocypha fenestrella (Rambur, 1842)

--Manuscript Draft--

**Manuscript Number:**	PONE-D-23-28970R1
**Article Type:**	Research Article
**Full Title:**	Genetic diversity and phylogeographic patterns of the peacock jewel-damselfly, Rhinocypha fenestrella (Rambur, 1842)
**Short Title:**	Genetic diversity and phylogeography of Rhinocypha fenestrella
**Corresponding Author:**	Noor Azrizal Abdul Wahid, Ph.D<br>Universiti Putra Malaysia Fakulti Sains<br>Serdang, Selangor MALAYSIA
**Keywords:**	cox1, haplotype, Malaysia, population structure, 16S
**Abstract:**	The present study examined the genetic diversity and phylogeographic pattern of the peacock jewel-damselfly Rhinocypha fenestrella by sequencing the cytochrome C oxidase I (cox1) and 16S rRNA gene regions from 147 individuals representing eight populations in Malaysia. A total of 26 and 10 unique haplotypes were revealed by the cox1 and 16S rRNA genes, respectively, and 32 haplotypes were recovered by the concatenated sequences of cox1+16S. Analyses indicated that haplotype AB2 was the most frequent and the most widespread haplotype in Malaysia, and the most recent common ancestor of the R. fenestrella may exist from the Negeri Sembilan as discovered from cox1+16S haplotype network analysis. Overall haplotype and nucleotide diversities of the concatenated sequences were Hd = 0.8937 and Pi = 0.0023, respectively, with great genetic differentiation (FST = 0.6387) and low gene flow (Nm = 0.14). Population from Pahang presented the highest genetic diversity (Hd = 0.8889, Pi = 0.0022, Nh = 9), whereas Kedah population demonstrated the lowest diversity (Hd = 0.2842, Pi = 0.0003, Nh = 4). The concatenated sequences of cox1+16S showed genetic divergence ranging from 0.09% to 0.97%, whereas the genetic divergence for cox1 and 16S rRNA genes were 0.16% to 1.63% and 0.01% to 0.75% respectively. This study provides for the first-time insights on the intraspecific genetic diversity, phylogeographic pattern and ancestral haplotype of Rhinocypha fenestrella.
**Order of Authors:**	Noor Azrizal Abdul Wahid, Ph.D
	Noorhidayah Mamat, Ph.D
	Van Lun Low, Ph.D
	Norma-Rashid Yusoff, Ph.D
**Response to Reviewers:**	The detail responses to the editors and reviewers have been provided as attached file (see .doc file with title "Response to Reviewers". Thank you.
**Additional Information:**	
**Question**	**Response**
**Financial Disclosure**<br><br>Enter a financial disclosure statement that describes the sources of funding for the work included in this submission. Review the submission guidelines for detailed requirements. View published research articles from *PLOS ONE* for specific examples.	Author who received the funding award= Norma-Rashid Yusoff;<br>Grant numbers awarded to the author= 1;<br>The full name of funder= The Universiti Malaya Research Grant (UMRG) PG065 – 2015 and ST061-2022;<br>URL of each funder website= https://umhvl.um.edu.my/grants-and-projects-awarded;<br>Role of the funder: Conceptualization, Investigation, Methodology, Project administration, Supervision

Powered by Editorial Manager® and ProduXion Manager® from Aries Systems Corporation

This statement is required for submission and **will appear in the published article** if the submission is accepted. Please make sure it is accurate.<br><br>**Unfunded studies**<br>Enter: *The author(s) received no specific funding for this work.*<br><br>**Funded studies**<br>Enter a statement with the following details:<br>• Initials of the authors who received each award<br>• Grant numbers awarded to each author<br>• The full name of each funder<br>• URL of each funder website<br>• Did the sponsors or funders play any role in the study design, data collection and analysis, decision to publish, or preparation of the manuscript?<br>• **NO** - Include this sentence at the end of your statement: *The funders had no role in study design, data collection and analysis, decision to publish, or preparation of the manuscript.*<br>• **YES** - Specify the role(s) played.<br><br>* typeset	
**Competing Interests**<br><br>Use the instructions below to enter a competing interest statement for this submission. On behalf of all authors, disclose any competing interests that could be perceived to bias this work—acknowledging all financial support and any other relevant financial or non-financial competing interests.<br><br>This statement is required for submission and **will appear in the published article** if the submission is accepted. Please make sure it is accurate and that any funding sources listed in your Funding Information later in the submission form are also declared in your Financial Disclosure statement.<br><br>View published research articles from *PLOS ONE* for specific examples.	The authors have declared that no competing interests exist

Powered by Editorial Manager® and ProduXion Manager® from Aries Systems Corporation

**NO authors have competing interests**<br><br>Enter: *The authors have declared that no competing interests exist.*<br><br>**Authors with competing interests**<br><br>Enter competing interest details beginning with this statement:<br><br>*I have read the journal's policy and the authors of this manuscript have the following competing interests: [insert competing interests here]*<br><br>* typeset	
**Ethics Statement**<br><br>Enter an ethics statement for this submission. This statement is required if the study involved:<br><br>• Human participants<br>• Human specimens or tissue<br>• Vertebrate animals or cephalopods<br>• Vertebrate embryos or tissues<br>• Field research<br><br>Write "N/A" if the submission does not require an ethics statement.<br><br>General guidance is provided below. Consult the submission guidelines for detailed instructions. **Make sure that all information entered here is included in the Methods section of the manuscript.**	N/A

**Format for specific study types**<br><br>**Human Subject Research (involving human participants and/or tissue)**<br>• Give the name of the institutional review board or ethics committee that approved the study<br>• Include the approval number and/or a statement indicating approval of this research<br>• Indicate the form of consent obtained (written/oral) or the reason that consent was not obtained (e.g. the data were analyzed anonymously)<br><br>**Animal Research (involving vertebrate animals, embryos or tissues)**<br>• Provide the name of the Institutional Animal Care and Use Committee (IACUC) or other relevant ethics board that reviewed the study protocol, and indicate whether they approved this research or granted a formal waiver of ethical approval<br>• Include an approval number if one was obtained<br>• If the study involved *non-human primates*, add *additional details* about animal welfare and steps taken to ameliorate suffering<br>• If anesthesia, euthanasia, or any kind of animal sacrifice is part of the study, include briefly which substances and/or methods were applied<br><br>**Field Research**<br><br>Include the following details if this study involves the collection of plant, animal, or other materials from a natural setting:<br>• Field permit number<br>• Name of the institution or relevant body that granted permission	
**Data Availability**<br><br>Authors are required to make all data underlying the findings described fully available, without restriction, and from the time of publication. PLOS allows rare exceptions to address legal and ethical concerns. See the PLOS Data Policy and FAQ for detailed information.	Yes - all data are fully available without restriction

A Data Availability Statement describing where the data can be found is required at submission. Your answers to this question constitute the Data Availability Statement and **will be published in the article**, if accepted.<br><br>**Important:** Stating 'data available on request from the author' is not sufficient. If your data are only available upon request, select 'No' for the first question and explain your exceptional situation in the text box.<br><br>Do the authors confirm that all data underlying the findings described in their manuscript are fully available without restriction?	
**Describe where the data may be found in full sentences. If you are copying our sample text, replace any instances of XXX with the appropriate details.**<br><br>• If the data are **held or will be held in a public repository**, include URLs, accession numbers or DOIs. If this information will only be available after acceptance, indicate this by ticking the box below. For example: *All XXX files are available from the XXX database (accession number(s) XXX, XXX.*).<br>• If the data are all contained **within the manuscript and/or Supporting Information files**, enter the following: *All relevant data are within the manuscript and its Supporting Information files.*<br>• If neither of these applies but you are able to provide **details of access elsewhere**, with or without limitations, please do so. For example:<br><br>*Data cannot be shared publicly because of [XXX]. Data are available from the XXX Institutional Data Access / Ethics Committee (contact via XXX) for researchers who meet the criteria for access to confidential data.*<br><br>*The data underlying the results presented in the study are available from (include the name of the third party*	All relevant data are within the manuscript and its Supporting Information files.

and contact information or URL).<br>• This text is appropriate if the data are owned by a third party and authors do not have permission to share the data.<br><br>* typeset	
Additional data availability information:	

Powered by Editorial Manager® and ProduXion Manager® from Aries Systems Corporation

Manuscript
Click here to access/download;Manuscript;Manuscript.docx

Research Paper

Genetic diversity and phylogeographic patterns of the peacock jewel-damselfly, *Rhinocypha fenestrella* (Rambur, 1842)

Mamat-Noorhidayah[1], Noor Azrizal-Wahid[2*], Van Lun Low[3], Norma-Rashid Yusoff[1]

[1] Institute of Biological Sciences, Faculty of Science, Universiti Malaya, Kuala Lumpur, Malaysia
[2] Department of Biology, Faculty of Science, Universiti Putra Malaysia, 43400 Serdang, Selangor, Malaysia
[3] Higher Institution Centre of Excellence (HICoE), Tropical Infectious Diseases Research & Education Centre (TIDREC), Universiti Malaya, 50603 Kuala Lumpur, Malaysia

*Corresponding author
Email: noorazrizal@upm.edu.my

Abstract

The present study examined the genetic diversity and phylogeographic pattern of the peacock jewel-damselfly *Rhinocypha fenestrella* (Rambur, 1842) by sequencing the cytochrome C oxidase I (*cox1*) and *16S* rRNA gene regions from 147 individuals representing eight populations in Malaysia. A total of 26 and 10 unique haplotypes were revealed by the *cox1* and *16S* rRNA genes, respectively, and 32 haplotypes were recovered by the concatenated sequences of *cox1+16S*. Analyses indicated that haplotype AB2 was the most frequent and the most widespread haplotype in Malaysia, and the most recent common ancestor of the *R.*

fenestrella may exist from the Negeri Sembilan as discovered from *cox1+16S* haplotype network analysis. Overall haplotype and nucleotide diversities of the concatenated sequences were H_d = 0.8937 and P_i = 0.0028, respectively, with great genetic differentiation (F_{ST} = 0.6387) and low gene flow (N_m = 0.14). Population from Pahang presented the highest genetic diversity (H_d = 0.8889, P_i = 0.0022, N_h = 9), whereas Kedah population demonstrated the lowest diversity (H_d = 0.2842, P_i = 0.0003, N_h = 4). The concatenated sequences of *cox1+16S* showed genetic divergence ranging from 0.09% to 0.97%, whereas the genetic divergence for *cox1* and *16S* rRNA genes were 0.16% to 1.63% and 0.01% to 0.75% respectively. This study provides for the first-time insights on the intraspecific genetic diversity, phylogeographic pattern and ancestral haplotype of *Rhinocypha fenestrella*.

Keywords: *cox1*, haplotype, Malaysia, population structure, *16S*

Introduction

Rhinocypha (Cowley, 1937), from the family of Chlorocyphidae, is the most characteristic genus of dragonflies in tropical Asia. Rhinocyphae is of a very particular nature in their habitat [1] and certain species can adapt to and tolerate disturbed habitats [2]. This characteristic makes them good "thermometers" of environment quality, thus good study subjects for phylogeography [3]. *Rhinocypha fenestrella* (Rambur, 1842), syn, *Aristocypha fenestrella*, also known as a peacock jewel, is the most widespread species in the genus, and one of the most active species of the family, which occurs in Peninsular Malaysia, Thailand, Burma, Laos, Vietnam, and southern China [4]. It is one of several common mountain stream damselflies and is usually found in the primary forests [1].

Malaysia is known to be one of the three mega-biodiversity countries in Southeast Asia, however, the phylogeographic pattern of dragonflies has been relatively scarce, especially for

this particular species. Several phylogeographic studies have been published concerning odonates [5-7], however, until now, no comparable research related to odonate species in Malaysia has been conducted. Instead, the species with a widespread distribution that includes dragonflies normally will have complexes of multiple lineages or variations in the genetic diversity with the geographic region [8-12]. From a point of zoogeography, the Rhinocyphae are of substantial importance, for instance, in the Malay Archipelago, each large island, mostly has its own group of endemic species [1].

A phylogeographic analysis is a powerful method of obtaining insights into the historical processes that have shaped the species' temporal distribution and genetic variation [13]. Recent phylogeographic research on widely distributed odonatan species has indicated different responses to historical climatic changes in space and time [14,15]. With the advances in molecular techniques, mitochondrial DNA has been identified as an excellent genetic marker of gene flow in matrilineal inheritance [16] and it is the most widely used marker to study the molecular ecology in animal taxa [17,18].

Particularly, cytochrome C oxidase subunit I (*cox1*) and 16S ribosomal RNA (*16S*) are known to be reliable genetic markers and the commonly applied markers in Odonata [7,19-21]. In addition to nuclear markers, mitochondrial markers were also provided well-resolved and supported trees from species to family level [22,23]. Given the high resolution of mitochondria-encoded *cox1* and *16S* genes reported in odonates [24], this study for the first time attempts to characterize the genetic diversity and population structure of *R. fenestrella*, across its range in Malaysia.

Materials and methods

Sample Collection

A total of 147 *Rhinocypha fenestrella* individuals were collected from eight populations representing eight states in Peninsular Malaysia from the period of 2014 to 2015 with the permission of the Forestry Department Peninsular Malaysia (Permit Number: JH/100 Jld.7 (12)) (Fig 1 and Table 1). The identification of *R. fenestrella* was performed according to morphological descriptions and taxonomic keys as described by Orr and Hamalainen (2003) [23], and through personal experience. Methods of sampling and preservation of Odonata were based on previously described standard methods [24]. Generally, samples were caught by using a sweep net and were dried preserved for morphological identification, while the legs were removed from each individual and stored in a vial containing ethanol for molecular works.

Fig 1. Map of sampling sites and haplotype distribution (AB1–AB15) of concatenated *cox1+16S* sequences for *Rhinocypha fenestrella* from eight sampling locations representing eight states of Peninsular Malaysia.

Table 1. Sampling localities and geographic position of sampling sites of *Rhinocypha fenestrella*.

State	District	Locality	Geographic position
N. Sembilan	Jelebu	Jeram Toi Waterfall	N 02 51' 52.7" E 102 00' 52.0"
Johor	Bekok	Sungai Bantang Waterfall	N 02 20' 46.7" E 103 09' 23.9"
Kedah	Banting	Lata Bayu Waterfall	N 05 43' 02.9" E 100 48' 50.9"
Perak	Tapah	Lata Iskandar Waterfall	N 04 19' 27.18" E 101 19' 31.36"
Kelantan	Machang	Jeram Linang Waterfall	N 05 44' 33.4" E 102 22' 26.2"
Terengganu	Kuala Berang	Sekayu Waterfall	N 04 57' 45.8" E 102 57' 11.9"
Pahang	Bentong	Chamang Waterfall	N 03 30' 34.2" E 101 51' 36.3"
Selangor	Hulu Selangor	Sungai Sendat Waterfall	N 03 24' 14.4" E 101 41' 00.5"

DNA extraction and amplification

Genomic DNA was extracted from four to six legs of each ethanol-preserved specimen using the i-genomic CTB DNA Extraction Mini Kit (iNtRON Biotechnology Inc., Seongnam, South Korea). The DNA amplifications of both *cox1* and *16S* genes were conducted using an Applied Biosystems Veriti 96-Well Thermal Cycler (Applied Biosystems Inc., Foster City, CA, USA) with the amplification protocol consisting of 300 sec at 94°C followed by 35 cycles of 50 sec at 94°C, 50 sec at 50°C and 50 sec at 72°C, and a final 7 min at 72°C. Primers for amplification of the *cox1* gene were 5'- GGT CAA CAA ATC ATA AAG ATA TTG G – 3' for forward primer [27] and 5'- GGA TGG CCA AAA AAT CAA AAT AAA TG –3' for reverse primer [28]. For the *16S* gene, ODO 12852 and ODO 13393 primer sets (forward primer, 5'- AGA AAC CGA CCT GGC TTA AA -3'; reverse primer, 5'- CGC CTG TTT ATC AAA AAC AT -3') were utilized [7]. Each PCR amplification was performed in a reaction mixture containing 50–100 ng of genomic DNA, 25 μl of NEXpro e-PCR 2X Master Mix (Genes Labs Inc., Gyeonggi-do, South Korea), and 10 pmol of each forward and reverse primer.

DNA purification, sequencing, and alignment

The amplified samples were then electrophoresed on 2% agarose gel pre-stained with SYBR Safe^{TM} (Invitrogen Corp., Carlsbad, CA, U.S.A.), and the PCR products were sent outsource to a commercial company (Apical Scientific Sdn. Bhd. Selangor, Malaysia) for DNA sequencing in both forward and reverse directions. The samples were sequenced using the BigDyeH Terminator 3.1 Sequencing Kit.

All sequences were assembled and edited using Molecular Evolutionary Genetics Analysis (MEGA) software Version 11.0 [29] and BioEdit 7.2 [30] and preliminarily aligned using CLUSTALX [31]. The aligned sequences were then subjected to a BLAST search (www.ncbi.nlm.nih.gov/blast/) for species validation.

Genetic diversity and haplotype analyses

The aligned cox1 and 16S sequences at first were analysed respectively, then were concatenated to yield a total length for further analysis. Molecular characteristics such as the number of haplotypes (N_h), haplotype diversity (H_d), nucleotide diversity (P_i), the number of segregating sites (S), and average number of sequence differences (K) were determined with the program DNASP® (DNA Sequence Polymorphism) [32]. DNASP® was also used to perform neutrality tests including Tajima's D [33] and Fu's F_s [34].

The F_{ST} and N_m pairwise values were calculated to access genetic differentiation and gene flow among the *R. fenestrella* population and were calculated using DNASP®. The levels of genetic differentiation are defined as $F_{ST} > 0.25$ (great differentiation), $0.15 < F_{ST} < 0.25$ (moderate differentiation), and $F_{ST} < 0.15$ (negligible differentiation) according to the classification criteria by Wright (1978) [35]. The levels of gene flow are categorized as $N_m > 1$ (high gene flow), $0.25 < N_m < 0.99$ (intermediate gene flow), and $N_m < 0.25$ (low gene flow) [36]. Uncorrected pairwise distances (*p*-distance) were assessed using PAUP 4.0B10 to measure the genetic divergence of *R. fenestrella*.

Furthermore, to visualize the phylogeographic pattern between the populations, and to calculate the minimum number of mutational steps between the sequences, haplotype networks were constructed using TCS 1.13® [37] with a 95% parsimony criterion for both *cox1* and *16S* respectively, and concatenated *cox1 + 16S* genes sequences.

Results

Genetic diversity of *Rhinocypha fenestrella*

The final aligned sequence fragment lengths were 614 bp, 534 bp, and 1148 bp for *cox1*, *16S,* and concatenated *cox1+16S*, respectively. The generated sequences that produce unique

haplotypes in this study were deposited in the National Center for Biotechnology Information (NCBI) GenBank database under accession numbers KY678719–KY678744 for *cox1*, and KY678745–KY678754 for *16S* genes.

The haplotype diversity (H_d) in a population for the *cox1* gene ranged from 0.1947 in the Kedah population to 0.8889 in the Pahang population. Nonetheless, for the *16S* gene, it ranged from 0.0000 found in three populations (Johor, Perak, and Selangor) to 0.2842 in the Kelantan population. In concatenated *cox1+16S* sequence analyses, the overall value of haplotype diversity was 0.8937, and nucleotide diversity (P_i) was 0.0028. Kedah population showed the lowest diversity for both nucleotide and haplotype diversities (H_d = 0.2842, P_i = 0.0003), while the highest haplotype diversity was shown by Pahang (H_d = 0.8889), and the highest nucleotide diversity was presented by the Kelantan population (P_i = 0.0023) (Table 2). In total average data estimates, the *cox1* gene revealed higher for both haplotype and nucleotide diversities (H_d = 0.8846, P_i = 0.0051) than did the *16S* gene (H_d = 0.1292, P_i = 0.0013).

Table 2: Genetic diversity indices and neutrality test based on *cox1*, *16S*, and concatenated *cox1+16S* sequences of *Rhinocypha fenestrella* from eight different populations in Peninsular Malaysia.

Markers	***Nh***	***Hd***	***Pi***	***S***	***K***	***D***	***Fs***
16S rRNA							
Johor	1	0.0000	0.0000	0	0	np	np
Kedah	2	0.1000	0.0002	1	0.1000	-1.1644	-0.879
Kelantan	4	0.2842	0.0010	4	0.4947	-1.9857*	-1.589
Negeri Sembilan	2	0.1053	0.0002	1	0.1053	-1.1648	-0.838
Pahang	3	0.1947	0.0054	15	2.7526	-1.2996	5.371
Perak	1	0.0000	0.0000	0	0.0000	np	np
Selangor	1	0.0000	0.0000	0	0.0000	np	np
Terengganu	4	0.2842	0.0031	15	1.5947	-2.4439*	1.627
**OVERALL**	**10**	**0.1292**	**0.0013**	**22**	**0.6850**	**-2.3808****	**-5.488**
cox1							
Johor	4	0.4316	0.001	3	0.4684	-1.1914	-1.713
Kedah	3	0.1947	0.000	2	0.2000	-1.5128	-1.863
Kelantan	4	0.7263	0.003	5	2.0158	1.3167	1.812

Negeri Sembilan	3	0.4328	0.0007	2	0.4561	-0.4849	-0.421
Pahang	9	0.8889	0.0040	9	2.4641	-0.2066	-2.838
Perak	5	0.5579	0.0013	5	0.7947	-1.3344	-1.711
Selangor	3	0.5636	0.0013	3	0.8000	-0.7494	0.158
Terengganu	6	0.7778	0.0028	6	1.7193	0.0049	-0.780
**OVERALL**	**26**	**0.8846**	**0.0051**	**28**	**3.0981**	**-1.1123**	**-9.824**
cox1+16S							
Johor	4	0.4316	0.0004	3	0.4684	-1.1914	-1.713
Kedah	4	0.2842	0.0003	3	0.3000	-1.7233	-2.749
Kelantan	8	0.8211	0.0023	10	2.6105	-0.5605	-1.297
Negeri Sembilan	4	0.5088	0.0005	3	0.5614	-0.9407	-1.355
Pahang	9	0.8889	0.0021	9	2.4641	-0.2066	-2.838
Perak	5	0.5579	0.0007	5	0.7947	-1.3344	-1.711
Selangor	3	0.5636	0.0007	3	0.8000	-0.7494	0.158
Terengganu	6	0.7778	0.0017	8	1.9298	-0.5366	-0.460
**OVERALL**	**32**	**0.8937**	**0.0028**	**37**	**3.2341**	**-1.5732**	**-16.523**

Haplotype variation and distribution

A set of aligned sequences of 147 taxa of the *cox1* revealed 26 haplotypes (A1 – A26). For *cox1*, the most prevalent haplotype was A2 (n = 40) and was found in five populations. The second most-frequent haplotypes were A1, A4, and A14 (n = 15) while the least prevalent haplotypes presented as singleton were A3, A5, A7 – A11, A18 – A20, A23, and A25. Notably, based on the star-like pattern of the haplotype network (Fig 2), the haplotypes A2 – A26 from the *cox1* gene were considered to be originated from haplotype A1.

Fig 2. Median joining haplotype network of *Rhinocypha fenestrella* of *cox1* sequences isolated from eight different states in Peninsular Malaysia. Each circle represents a haplotype variation, and the size of a circle is proportional to the number of sequences assigned to that haplotype. Circles of the same colour represent haplotypes from the same population. A small black square represents median vectors.

For the *16S* gene, the sequences revealed 10 haplotypes (B1 – B10). The most prevalent haplotype was B1 (n = 140) while all other haplotypes except B8 (n = 2), appeared as singleton haplotypes. Haplotype B1 was found in all populations. The haplotype network of *16S* gene showed a star-like pattern suggesting haplotype B1 as a common ancestor of all the others (Fig 3). Nevertheless, a median-joining network showed no obvious geographical pattern in haplotype distribution.

Fig 3. Median joining haplotype network of *Rhinocypha fenestrella* of *16S* sequences isolated from eight different states in Peninsular Malaysia. Each circle represents a haplotype variation, and the size of a circle is proportional to the number of sequences assigned to that haplotype. Circles of the same colour represent haplotypes from the same population. A small black square represents median vectors.

The concatenated *cox1+16S* sequences revealed 32 haplotypes (AB1 – AB32). Haplotype AB2 was the most frequent and the most widespread haplotype based on its prevalence in Malaysia, while there were 18 singleton haplotypes (AB3, AB4, AB6, AB8 – AB12, AB15, AB18, AB20, AB21, AB 23 – AB26, AB29, and AB31 (Table 3). Furthermore, the Kelantan population presented as the most diverse population that had the highest number of haplotypes (N_h = 30) while the least number of haplotypes was presented in the Selangor population (N_h = 11). Haplotype AB1 appeared to be as a common ancestor of the other haplotypes as indicated by the star-like pattern of concatenated *cox1+16S* haplotype network (Fig 4). Consequently, based of sampling sites, this study revealed that a recent common ancestor of the *R. fenestrella* species in Malaysia existed from the Negeri Sembilan population that constituted the most numbers of haplotype AB1.

Fig 4. Median joining haplotype network of *Rhinocypha fenestrella* of concatenated *cox1+16S* sequences isolated from eight different states in Peninsular Malaysia. Each circle represents a haplotype variation, and the size of a circle is proportional to the number of sequences assigned to that haplotype. Circles of the same colour represent haplotypes from the same population. A small black square represents median vectors.

Table 3. **Haplotype distribution of *Rhinocypha fenestrella* (*n* = 147) from Peninsular Malaysia based on the concatenated *cox1+16S* sequences corresponding to the eight sampling sites.**

Sampling sites	*n*	Haplotype *cox1 + 16S rRNA*																															
		AB1	AB2	AB3	AB4	AB5	AB6	AB7	AB8	AB9	AB10	AB11	AB12	AB13	AB14	AB15	AB16	AB17	AB18	AB19	AB20	AB21	AB22	AB23	AB24	AB25	AB26	AB27	AB28	AB29	AB30	AB31	AB32
**N. Sembilan**	19	13	4	1	1	-	-	-	-	-	-	-	-	-	-	-	-	-	-	-	-	-	-	-	-	-	-	-	-	-	-	-	-
**Johor**	20	-	-	-	-	15	1	3	1	-	-	-	-	-	-	-	-	-	-	-	-	-	-	-	-	-	-	-	-	-	-	-	-
**Kedah**	20	-	17	-	-	-	-	-	-	1	1	1	-	-	-	-	-	-	-	-	-	-	-	-	-	-	-	-	-	-	-	-	-
**Perak**	20	-	13	-	-	-	-	-	-	-	-	-	1	1	4	1	-	-	-	-	-	-	-	-	-	-	-	-	-	-	-	-	-
**Kelantan**	20	-	-	-	-	-	-	-	-	-	-	-	-	-	-	-	13	7	1	5	1	1	1	1	-	-	-	-	-	-	-	-	-
**Terengganu**	19	-	4	-	-	-	-	-	-	-	-	-	-	-	-	-	8	-	-	3	-	-	2	-	1	1	-	-	-	-	-	-	-
**Pahang**	18	1	1	-	-	-	-	-	-	-	-	-	-	-	-	-	2	-	-	-	-	-	-	-	-	-	1	3	3	1	5	1	-
**Selangor**	11	-	-	-	-	-	-	-	-	-	-	-	-	1	-	-	-	-	-	-	-	-	-	-	-	-	-	7	-	-	-	-	3
**TOTAL**	**147**	**14**	**39**	**1**	**1**	**15**	**1**	**3**	**1**	**1**	**1**	**1**	**1**	**2**	**4**	**1**	**23**	**7**	**1**	**8**	**1**	**1**	**3**	**1**	**1**	**1**	**1**	**10**	**3**	**1**	**5**	**1**	**3**

n = number of odonates

Haplotype genetic divergence

The *p*-distance between *R. fenestrella* haplotypes for *cox1* ranged from 0.16% – 1.63% with the highest value showed pairwise between haplotypes A5 with A15, A17, A19, and between haplotypes A7 with A15, A17, A19 (S1 Table). On the other hand, the genetic divergence of *16S* haplotypes ranged from 0.01% – 0.75% with the highest value presented pairwise between haplotypes B5 and B7 (S2 Table). For the concatenated *cox1+16S*, the genetic divergence ranged from 0.09% to 0.97% (S3 Table).

In concatenated *cox1+16S* sequences, the neutrality test of Fu's F_s showed negative values in all populations with an exception for Selangor. Additionally, Tajima's *D* tests for all populations were indicated by negative values and were not statistically significant. The overall F_{ST} was 0.6387 and N_m was 0.14, suggesting that the genetic differentiation was great while there was a low gene flow among the populations of *R. fenestrella* in Malaysia. Based on Table 4, the highest F_{ST} value (0.0100) appeared between Perak and Selangor populations while the lowest value (0.0010) was between the populations of Negeri Sembilan and Kedah, Negeri Sembilan and Perak, Kedah, and Perak, Kedah and Pahang, and Kedah and Selangor.

Table 4. Pairwise genetic differentiation (F_{ST}) among *Rhinocypha fenestrella* populations in eight distinct populations in Peninsular Malaysia.

Population	S1	S2	S3	S4	S5	S6	S7	S8
**N. Sembilan**	-	-	-	-	-	-	-	-
**Johor**	0.006	0.005	-	-	-	-	-	-
**Kedah**	0.001	0.006	0.001	-	-	-	-	-
**Perak**	0.001	0.007	0.002	0.002	-	-	-	-
**Kelantan**	0.003	0.007	0.002	0.002	0.002	-	-	-
**Terengganu**	0.002	0.007	0.001	0.002	0.003	0.003	-	-
**Pahang**	0.002	0.006	0.001	0.010	0.003	0.003	0.002	-

Discussion

Genetic diversity of Malaysian *Rhinocypha fenestrella*

Throughout the last five decades, the understanding of the ecology and evolution of odonates has increased dramatically. Recent advances in molecular techniques have inspired several phylogeographical studies of Odonata using genetic data [24,38,39]. As is known, most of the odonates have varying levels of dispersal abilities [40] that could influence the genetic diversity and phylogeographic structures of the populations. A less-mobile species may be expected to show some evidence of haplotype clustering according to geographic region [41]. Therefore, in this study, we defined the intraspecific genetic diversity, phylogeographical patterns, and mitochondrial variations of the *Rhinocypha fenestrella* using *cox1* and *16S* genes.

The obtained sequences for *cox1* and *16S* genes from eight populations ranged from 1.5 – 4.6% of variability among all studied individuals reflected in 28 variable nucleotide sites in 614 sequenced base pairs from *cox1* gene, and eight variable nucleotides in 534 base pairs from *16S* gene. The base composition of both *cox1* and *16S* gene sequences showed a significant A+T nucleotide bias which is consistent with insect mitochondrial genes [38,42,43].

In the present study, overall genetic diversity (H_d = 0.8937; P_i = 0.0028) of Malaysian *R. fenestrella* based on the concatenated *cox1+16S* sequences was slightly lower if compared to the study that did on the damselfly *Matrona basilaris* populations from mainland China [44]. The study reported higher values of haplotype diversity (H_d = 0.9750) and nucleotide diversity (P_i = 0.0049) by using *cox1* sequences. However, when compared with the study on *Pantala flavescens* (mean H_d = 0.8100; mean P_i = 0.0060) by using *Cytb* marker [45], the genetic diversity of this study was found to be slightly higher. Nevertheless, the genetic diversity of *R. fenestrella* in Malaysia can be considered as high when compared to other damselflies species from the same sub-order *Psolodesmus mandarinus* (MacLahlan, 1870) from Taiwan

that reported haplotype diversity as 0.43 and nucleotide diversity as 0.0017 based on *cox1+16S* sequences [46].

Haplotype variation and distribution of *Rhinocypha fenestrella*

In this study, the number of haplotypes detected was considered high with 26 haplotypes were revealed by *cox1* and 10 haplotypes by *16S*. The high numbers of haplotypes may indicate a high degree of *cox1* and *16S* gene polymorphisms in *R. fenestrella* samples in Peninsular Malaysia. In fact, the polymorphism that occurred in Odonata were widely reported in other studies [47,48]. Moreover, this study demonstrated extremely high numbers of haplotypes when comparing to the study by Lin et al. (2012) [46] that reported only 14 haplotypes variation in the damselfly population of *Psolodesmus mandarinus* from Taiwan, while the dragonfly population from Scandinavia and Finland only revealed of four haplotype variations [49].

The haplotype AB2 appeared as the most dominant haplotype, while the limited geographical distribution of some of the haplotypes including those found as singleton suggests the existence of genetic differentiation within the populations. In this study, haplotype AB1 was suggested as the common ancestor due to star-like radiation in the haplotype network pattern. This haplotype probably had eventually evolved over time into the numerous haplotypes (AB2–AB15) in order to adapt to the habitat and demographic changes and consequently distributed across states in Peninsular Malaysia. Likewise, according to location-based, the most recent common ancestor for *R. fenestrella* in Malaysia may be derived from Negeri Sembilan as this state is constituted by high frequencies of the common ancestor AB1 haplotypes.

The median-joining network also revealed a close relationship among haplotypes, suggesting that *R. fenestrella* populations in Malaysia shared a recent history without long-term genetic isolation. Nevertheless, the ancestral haplotype AB1 of concatenated *cox1+16S*

dataset was having a limited distribution with restricted geographic division. This indicates that geographic barriers and climatic factors could also have little influence on the dispersion of this damselflies into different habitats in Malaysia besides their less ability to disperse due to weak flyers.

Genetic distance and differentiation

In our dataset, the highest genetic distance value based on *cox1* gene was 0.16%, while *16S* gene was 0.75%. The data shows a higher genetic distance among Malaysian *R. fenestrella* as compared to the populations of *Rhinocypha taiwana* (Wang & Chang, 2013), *Rhinocypha uenoi* (Asahina, 1964), and *Rhinocypha drusilla* (Needham, 1930) from China where the highest genetic distances based on *cox1* gene were 0.00%, 0.00% and 0.50%, respectively [50]. However, the genetic distance of Malaysian *R. fenestralla* was relatively lower if compared to other dragonfly populations of *Trithemis stictica* (Burmeister, 1839) in Namibia [9] and *Nannophya pygmaea* (Rambur, 1842) in Malaysia [51] that presented the genetic distance of *cox1* gene ranging from 0.00% - 9.00% and 0.00% - 12.00%, respectively.

Zygopterans are known as weaker fliers since many species do not diffusing outside several kilometres [52,53]. *Rhinocypha fenestrella* is known as a weak flyer and is unlikely to migrate over large-scale regions, which likely contributes to its overall high genetic differentiation to happen in and between the populations. However, in the present study, it is somewhat surprising that genetic differentiation happened in the species *R. fenestrella* in which their populations were separated up to more than 500 km. Similarly, the migratory behaviour of odonate could homogenize genetic differentiation among populations by the exchange of individuals and genes among populations with high genetic differentiation has been observed in the population of migratory dragonflies *Libellula quadrimaculata* (Linnaeus, 1758) [38] and

Pantala flavescens (Fabricius, 1798) [54] respectively which are known as strong fliers than the damselflies.

Dispersal ability and long-distance migration are the most important factors contributing to a high level of gene flow and consequent slowing or limitating of geographic differentiation [55,56]. A less-mobile species may be expected to show some evidence of haplotype clustering according to geographic region due to less ability to disperse and therefore resulted in genetic isolation by distance. In this study, calculated F_{st} value shows high genetic differentiation in the populations of *R. fenestrella* in Malaysia. This high value is consequently supported by the low rate of gene flow found amongst the *R. fenestrella* populations in Malaysia as well as between the different sites.

When all eight populations were regarded as a whole, Tajima's D statistic was statistically not significant. The results showed that these populations were in a stable state with no demographic expansion and no recent bottleneck. Whereas, in the Selangor population, Fu's F_S statistic values were negative with *p*-values being significant ($p < 0.02$) which shows this population had experienced a recent population expansion. Our results suggested that *R. fenestrella* population in Selangor could successfully colonize and adapt to new habitats and were able to disperse randomly and exchange genes with local populations.

Maintaining genetic diversity is noteworthy as it related to population viability [57-59] and also to the transformative potential of a species to react to the environmental changes [60,61].

Overall, this study found strong evidence for intraspecific patterns of haplotype variation among populations of *R. fenestrella* in Malaysia though the weak dispersal abilities of the species. The study also revealed high genetic differentiation within the populations and a low rate of gene flow among the geographically difference populations of Malaysian *R. fenestrella*. Moreover, a high haplotype number was observed in *R. fenestrella* population, but no genetic isolation was found within the populations of the sampling sites. Although many

phylogeographical mechanisms have been proposed using damselflies as model organisms, more detailed sampling and a larger variety of ecological investigations are required to promote better understanding. Therefore, this study contributed new insight for an advanced understanding of the evolution and phylogeography of damselfly. Additional sequence data from another regions (Thailand, Burma, Laos, Vietnam, and southern China), which is considered a gap of knowledge due to the lack of genetic data on this particular species, may prove useful in detecting phylogenetic relationships and phylogeography patterns, and revealing common ancestors of these populations between the other regions and continents.

Competing Interests

The authors have declared that no competing interests exist.

Acknowledgements

The authors would like to thank Mr. Mohaiyidin Mohamed and Mr. Mohd Fauzi Abd Hamid for their assistance in the fieldwork.

References

1. Lieftinck MA. Two interesting new insular *Rhinocypha* from Malaysia (Odon.). Tijdschrift Voor Entomologie. 1947; 88: 215–224.
2. Mamat N, Abu A, Yusoff NR. Classification and morphology of *Rhinocypha* spp. (Odonata): A comprehensive taxonomic study within the females. Zoological Studies. 2021; 26(60): e47.

3. Córdoba–Aguilar A. Introduction. In: Córdoba–Aguilar A, editor. Dragonflies and damselflies: study models in ecological and evolutionary research. Oxford (UK): Oxford University Press; 2008. p. 1–3.

4. Sharma G. *Rhinocypha fenestrella*. The IUCN Red List of Threatened Species. 2010.

5. Kahilainen A, Puurtinen M, Kotiaho JS. Conservation implications of species–genetic diversity correlations. Global Ecology and Conservation. 2014; 2: 315–323.

6. Monroe E, Britten B. Conservation in Hine's sight: the conservation genetics of the federally endangered Hine's emerald dragonfly, Somatochlora hineana. Journal of Insect Conservation. 2014; 18: 353-363.

7. Dijkstra KDB, Kalkman VJ, Dow RA, Stokvis FR, Tol JV. Redefining the damselfly families: the first comprehensive molecular phylogeny of Zygoptera (Odonata). Systematic Entomology. 2014; 39(1): 68–96.

8. Angulo A, Icochea J. Cryptic species complexes, widespread species and conservation: lessons from Amazonian frogs of the *Leptodactylus marmoratus* group (Anura: Leptodactylidae). Systematics and Biodiversity. 2010; 8: 357–370.

9. Damm S, Schierwater B, Hadrys H. An integrative approach to species discovery in odonates: from character-based DNA barcoding to ecology. Molecular Ecology. 2010; 19: 3881–3893.

10. Low VL, Takaoka H, Adler PH, Ya'cob Z, Norma-Rashid Y, Chen CD, Sofian-Azirun M. A multi-locus approach resolves the phylogenetic relationships of the *Simulium asakoae* and *Simulium ceylonicum* species groups (Diptera: Simuliidae) in Malaysia: evidence for distinct evolutionary lineages. Medical and Veterinary Entomology. 2015a; 29: 330–337.

11. Low VL, Tay ST, Kho KL, Koh FX, Tan TK, Lim YAL, Panchadcharam C, Ong BL, Norma-Rashid Y, Sofian-Azirun M. Molecular characterisation of the tick *Rhipicephalus*

microplus in Malaysia: new insights into the cryptic diversity and distinct genetic assemblages throughout the world. Parasites and Vectors. 2015b; 8: 341.

12. Low VL, Takaoka H, Pramual P, Adler PH, Ya'cob Z, Huang YT, Pham XD, Ramli R, Chen CD, Wannaket A, Sofian-Azirun M. Delineating taxonomic boundaries in the largest species complex of black flies (Simuliidae) in the Oriental Region. Scientific Reports. 2016a; 6: 20346.
13. Avise JC. *Phylogeography*: The History and Formation of Species. Harvard University Press, Cambridge. 2000.
14. Kohli M, Letsch H, Greve C, Béthoux O, Deregnaucourt I, Liu S, Zhou X, Donath A, Mayer C, Podsiadlowski L, Gunkel S, Machida R, Niehuis O, Rust J, Wappler T, Yu X, Misof B, Ware J. Evolutionary history and divergence times of Odonata (dragonflies and damselflies) revealed through transcriptomics. iScience. 2021; 24(11): 103324.
15. Cancellario T, Miranda R, Baquero E, Fontaneto D, Martínez A, Mammola S. Climate change will redefine taxonomic, functional, and phylogenetic diversity of Odonata in space and time. npj Biodiversity. 2022; 1(1): 1-14.
16. Jisha KEK, Sebastian CD. Genetic variation and phylogeny assessment of *Aciagrion Occidentale* (Odonata: Coenagrionidae) using mitochondrial cytochrome oxidase subunit I gene. International Journal of Science and Research. 2013; 4(4): 1121–1123.
17. Norris DE. Genetic markers for study of the anopheline vectors of human malaria. Int Journal of Parasitology. 2002; 32: 1607–1615.
18. Pramual P, Kuvangkadilok C, Baimai V, Walton C. Phylogeography of the black fly *Simulium tani* (Diptera: Simuliidae) from Thailand as inferred from mtDNA sequences. Molecular Ecology. 2005; 14: 3989–4001.
19. Artiss T, Schultz TR, Polhemus DA, Simon C. Molecular phylogenetic analysis of dragonfly genera *Libellula*, *Ladona* and *Plathemis* (Odonata: Libelluinidae) based on

mitochondrial cytochrome oxidase I and 16S rRNA sequence data. Molecular Phylogenetics and Evolution. 2001; 18: 348–361.

20. Yong HS, Lim P-E, Tan J, Ng YF, Eamsobhana P, Suana IW. Molecular phylogeny of *Orthetrum* dragonflies reveals cryptic species of *Orthetrum pruinosum*. Scientific Reports. 2014; 4: 5553.
21. Kim MJ, Jung KS, Park, NS, Wan X, Kim KG, Jun J, Jun J, Yoon TJ, Bae YJ, Lee SM, Kim I. Molecular phylogeny of the higher taxa of Odonata (Insecta) inferred from COI, 16S rRNA, 28S rRNA, and EF1-α sequences. Entomological Research. 2014; 44(2): 65–79.
22. Gaines CA, Hare MP, Beck SE, Rosenbaum HC. Nuclear markers confirm taxonomic status and relationships among highly endangered and closely related right whale species. Proceedings in Biological Sciences. 2005; 272(1562): 533–542.
23. Ballare EF, Ware JL. Dragons fly, biologists classify: an overview of molecular odonate studies, and our evolutionary understanding of dragonfly and damselfly (Insecta: Odonata) behavior. International Journal of Odonatology. 2011; 14: 137–147.
24. Ožana S, Dolný A, Páne T. Nuclear copies of mitochondrial DNA as a potential problem for phylogenetic and population genetic studies of Odonata. Systematic Entomology. 2022; 47(4): 591–602.
25. Orr AG, Hamalainen M. *A Guide to the Dragonflies of Borneo: their Identification and Biology*. Natural History Publications, Borneo. 2003.
26. Orr D. The constitution of nature. Conservation Biology. 2003; 17: 1478–1484.
27. Folmer O, Black M, Hoeh W, Lutz R, Vrijenhoek R. DNA primers for amplification of mitochondrial cytochrome C oxidase subunit I from diverse metazoan invertebrates. Molecular Marine Biology and Biotechnology. 1994; 3(5): 294–299.

28. Barrett RDH, Hebert PDN. Identifying spiders through DNA barcodes. Canadian Journal of Zoology. 2005; 83(3): 481–491.
29. Kumar S, Stecher G, Tamura K. MEGA7: Molecular Evolutionary Genetics Analysis Version 7.0 for Bigger Datasets. Molecular Biology and Evolution. 2016; 33(7): 1870–1874.
30. Hall TA. BioEdit: a user-friendly biological sequence alignment editor and analysis program for Windows 95/98/NT. Nucleic Acids Symposium Series. 1999; 41(2): 95–98.
31. Thompson JD, Gibson TJ, Plewniak F, Jeanmougin F, Higgins DG. The ClustalX Windows interface: flexible strategies for multiple sequence alignment aided by quality analysis tools. Nucleic Acids Research. 1997; 24: 4876–4882.
32. Librado P, Rozas J. DNASP v5: A software for comprehensive analysis of DNA polymorphism data. Bioinformatics. 2009; 25: 1451–1452.
33. Tajima F. Statistical method for testing the neutral mutation hypothesis by DNA polymorphism. Genetics. 1989; 123: 585–595.
34. Fu YX. Statistical tests of neutrality of mutations against population growth, hitchhiking and background selection. Genetics. 1997; 147: 915–925.
35. Wright S. *Evolution and Genetics of Populations*. 1978. University of Chicago, Chicago.
36. Slatkin M. Estimating levels of gene flow in natural populations. Genetics. 1981; 99: 323–335.
37. Clement M, Posada D, Crandall KA. TCS: a computer program to estimate gene genealogies. Molecular Ecology. 2000; 9(10): 1657–1659.
38. Artiss T. Phylogeography of a facultatively migratory dragonfly, *Libellula quadrimaculata* (Odonata: Anisoptera). Hydrobiologia. 2004; 515: 225–234.
39. Simonsen T, Olsen K, Djernaes M. The African-Iberian connection in Odonata: mtDNA and ncDNA based phylogeography of Aeshna cyanea (Müller, 1764) (Odonata:

Aeshnidae) in Western Palaearctic. Arthropod Systematics and Phylogeny. 2020; 78(2): 309-320.

40. Rundle SD, Bilton DT, Foggo A. Body Size: The Structure and Function of Aquatic Ecosystems. In: Hildrew AG, Raffaelli DG, Edmonds-Brown R. editors. By wind, wings or water: body size, dispersal and range size in aquatic invertebrates. Cambridge (UK): Cambridge University Press; 2007. p. 186–209.
41. Shannon J, McCauley SJ, Davis CJ, Werner EE, Robeson MS. Dispersal, niche breadth and population extinction: colonization ratios predict range size in North American dragonflies. Journal of Animal Ecology. 2014; 83: 858–865.
42. Frati F, Simon C, Sullivan J, Swofford DL. Evolution of the mitochondrial cytochrome oxidase II gene in Collembola. Journal of Molecular Evolution. 1997; 44(2): 145–158.
43. Chippindale PT, Whitmore DH, Davé VK, Valencia TG, Robinson JV. Effective procedures for the extraction, amplification and sequencing of Odonate DNA. Odonatologica. 1998; 27: 415–424.
44. Xue J, Zhang H, Ning X, Bu W, Yu X. Evolutionary history of a beautiful damselfly, Matrona basilaris, revealed by phylogeographic analyses: the first study of an odonate species in mainland China. Heredity (Edinb). 2019; 122(5): 570-581.
45. Cao L, Wu K. Genetic diversity and demographic history of globe skimmers (Odonata: Libellulidae) in China based on microsatellite and mitochondrial DNA markers. Scientific Reports. 2019; 9: 8619
46. Lin SC, Chen YF, Shieh SH, Yang PS. Patterns of mitochondrial and wing morphological differentiation in Taiwanese populations of *Psolodesmus mandarinus* McLachlan (Zygoptera: Calopterygidae). Odonatologica. 2012; 41(2): 109–121.
47. Sánchez-Guillén R, Ceccarelli S, Villalobos F, Neupane S, Rivas-Torres A, Sanmartín-Villar I, Wellenreuther M, Bybee SM, Velásquez-Vélez MI, Realpe E, Chávez-Ríos JR,

Dumont HJ, Cordero-Rivera A. The evolutionary history of colour polymorphism in Ischnura damselflies (Odonata: Coenagrionidae). Odonatologica. 2020; 49: 333–370.

48. Joshi S, van der Poorten N, Sumanapala A, Nielsen E, Patel J, Nielsen B. New records of polymorphism in Asian libellulid dragonflies (Insecta: Odonata). International Journal of Odonatology. 2020; 23(4): 337–356.

49. Kohli MK, Sahlén G, Kuhn WR, Ware JL. Extremely low genetic diversity in a circumpolar dragonfly species, *Somatochlora sahlbergi* (Insecta: Odonata: Anisoptera). Scientific Reports. 2018; 8(1): 15114.

50. Wang LJ, Cherng JJ, Chang YJ, Jiang JL. Description of *Rhinocypha taiwana* sp. nov. from Taiwan, with a preliminary molecular phylogenetic analysis of the *Rhinocypha drusilla*-group (Odonata: Chlorocyphidae). International Journal of Odonatology. 2013; 16: 93–107.

51. Low VL, Sofian-Azirun M, Norma-Rashid Y. Playing hide-and-seek with the tiny dragonfly: DNA barcoding discriminates multiple lineages of *Nannophya pygmaea* in Asia. Journal of Insect Conservation. 2016; 20: 339–343.

52. Purse BV, Hopkins GW, Day KJ, Thompson DJ. Dispersal characteristics and management of a rare damselfly. Journal of Applied Ecology. 2003; 40: 716–728.

53. Watts PC, Rouquette JR, Saccheri IJ, Kemp SJ, Thompson DJ. Molecular and ecological evidence for small-scale isolation by distance in an endangered damselfly, *Coenagrion mercuriale*. Molecular Ecology. 2004; 13: 2931–2945.

54. Low VL, Norma-Rashid Y, Yusoff A, Vinnie-Siow WY, Prakash BK, Tan TK, Noorhidayah M, Chen CD, Sofian-Azirun M. Pleistocene demographic expansion and high gene flow in the Globe Skimmer dragonfly *Pantala flavescens* Fabricius (Odonata: Libellulidae) in Peninsular Malaysia. Zoologischer Anzeiger. 2017; 266: 23–27.

55. Rowe RJ. Conservation of Odonata in the South Pacific and Australasia. International Journal of Odonatology. 2004; 7(2): 139–147.
56. Ware J, Kohli MK, Mendoza CM, Troast D, Jinguji H, Hobson KA, Sahlén G, Anderson RC, Suhling F. Evidence for widespread gene flow and migration in the Globe Skimmer dragonfly *Pantala flavescens*. International Journal of Odonatology. 2022; 25: 43–55.
57. Saccheri IJ, Kuussaari M, Kankare M, Vikman P, Fortelius W, Hanski I. Inbreeding and extinction in a butterfly metapopulation. Nature. 1998; 392: 491–494.
58. Madsen T, Shine R, Olsson M, Wittzell H. Conservation biology – restoration of an inbred adder population. Nature. 1999; 402: 34–35.
59. Spielman D, Brook BW, Briscoe BW, Frankham R. Does inbreeding and a loss of genetic diversity decrease disease resistance?. Conservation Genetics. 2004; 5: 439–448.
60. Frankham R, Ballou JD, Briscoe DA. *Introduction to Conservation Genetics*. Cambridge University Press, Cambridge. 2002.
61. Reed DH, Frankham R. Fitness is correlated with genetic diversity. Conservation Biology. 2003; 17: 1–9.

Supporting Information

S1 Table. Percentage (%) of uncorrected "*p*" distance matrix among the 26 representative *cox1* haplotypes of *Rhinocypha fenestrella* in Malaysia.

S2 Table. Percentage (%) of uncorrected "*p*" distance matrix among the 10 representative *16S rRNA* haplotypes of *Rhinocypha fenestrella* in Malaysia.

S3 Table. Percentage (%) of uncorrected "*p*" distance matrix among the 32 representatives for the concatenated *cox1+16S* haplotypes of *Rhinocypha fenestrella* in Malaysia.

Author Contributions

Conceptualization: Norma-Rashid Yusoff, Mamat-Noorhidayah

Data curation: Mamat-Noorhidayah

Formal analysis: Mamat-Noorhidayah, Noor Azrizal-Wahid, Van Lun Low,

Funding acquisition: Norma-Rashid Yusoff, Mamat-Noorhidayah

Investigation: Mamat-Noorhidayah**,** Norma-Rashid Yusoff

Methodology: Mamat-Noorhidayah, Norma-Rashid Yusoff

Project administration: Norma-Rashid Yusoff

Supervision: Norma-Rashid Yusoff

Visualization: Mamat-Noorhidayah, Noor Azrizal-Wahid, Van Lun Low

Writing – original draft: Mamat-Noorhidayah, Noor Azrizal-Wahid

Writing – review & editing: Noor Azrizal-Wahid, Van Lun Low

Fig 1

Click here to access/download;Figure;Fig 1.tif

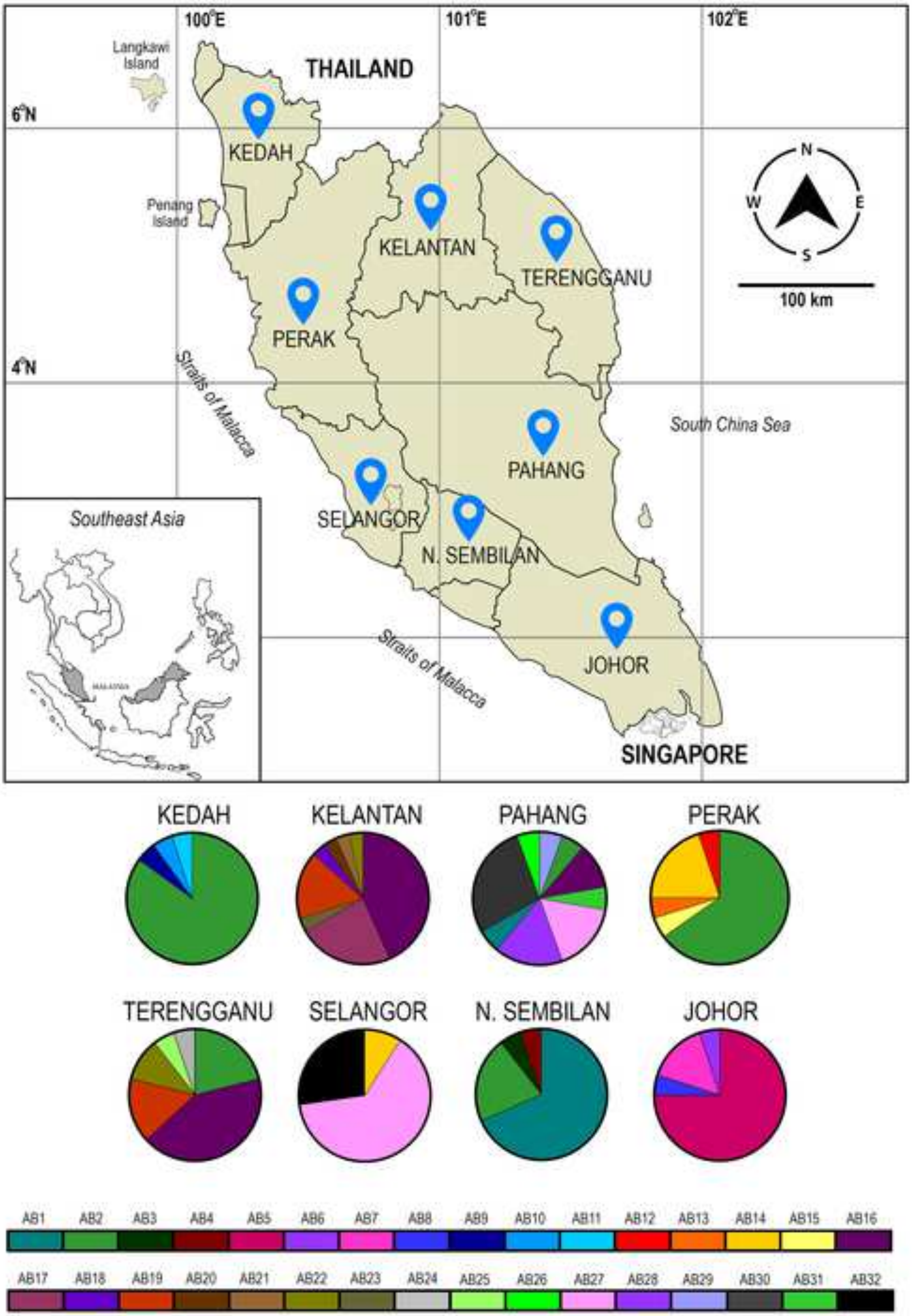

Fig 2

Click here to access/download;Figure;Fig 2.tif

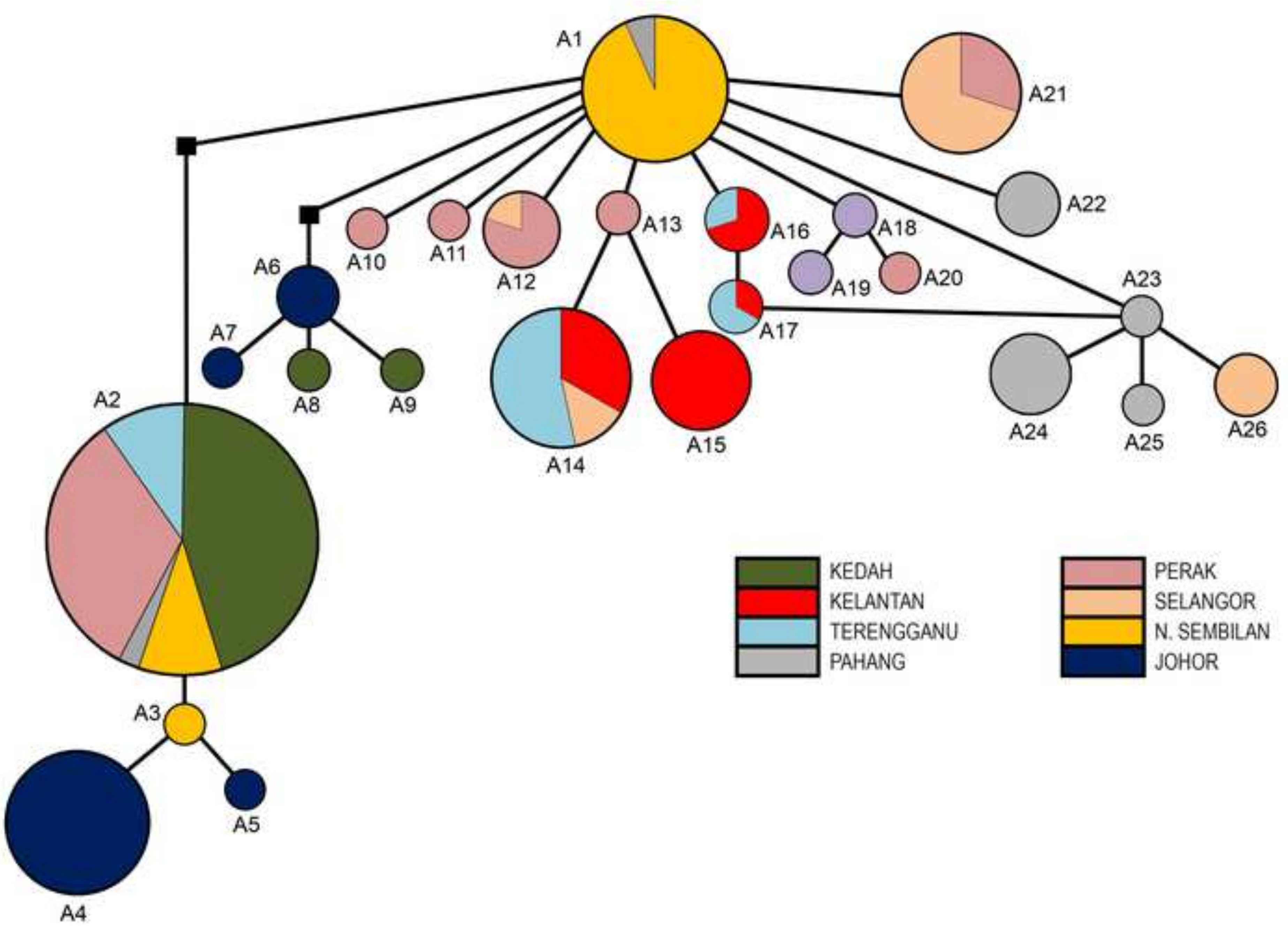

Click here to access/download;Figure;Fig 3.tif

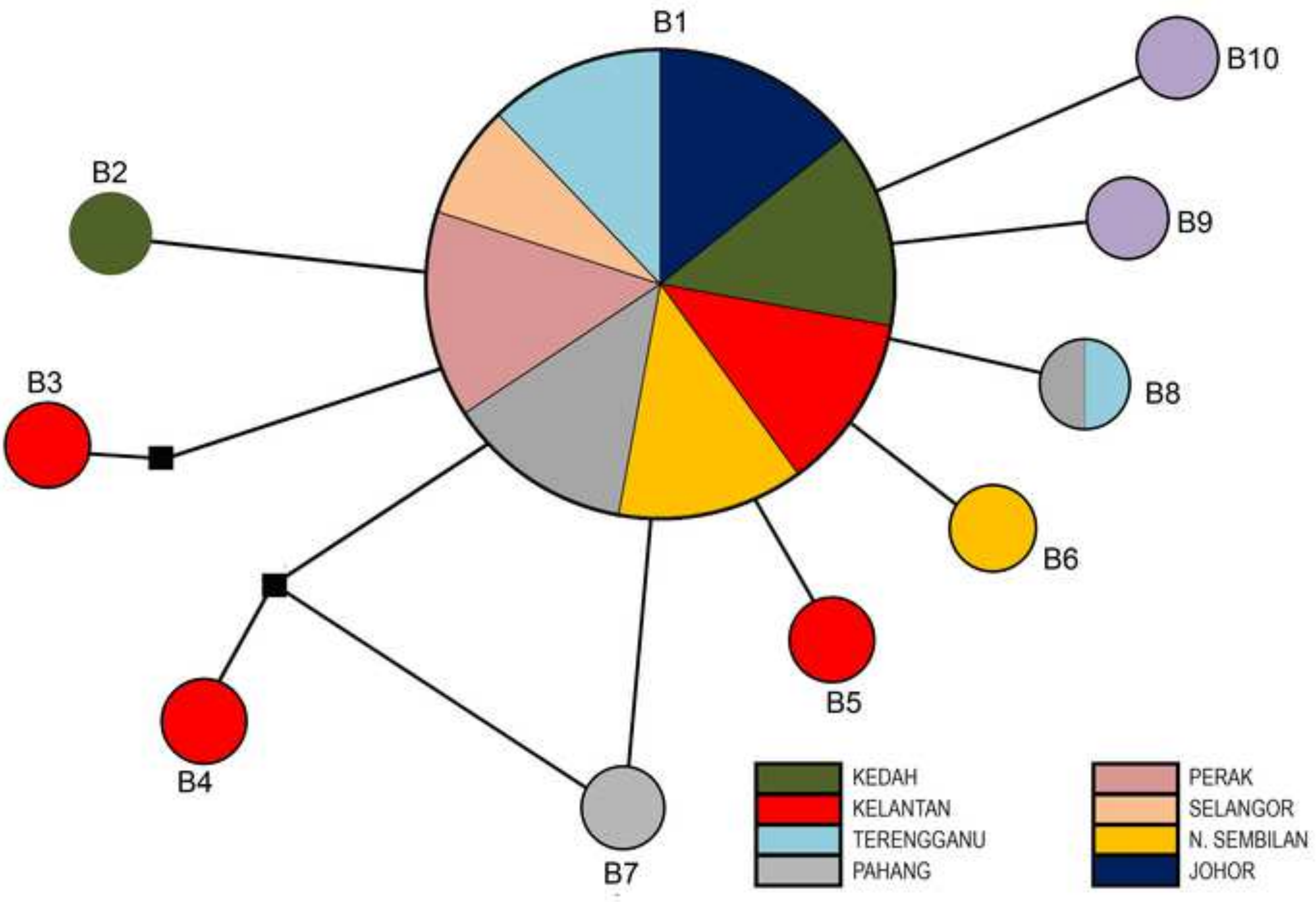

Fig 4

Click here to access/download;Figure;Fig 4.tif

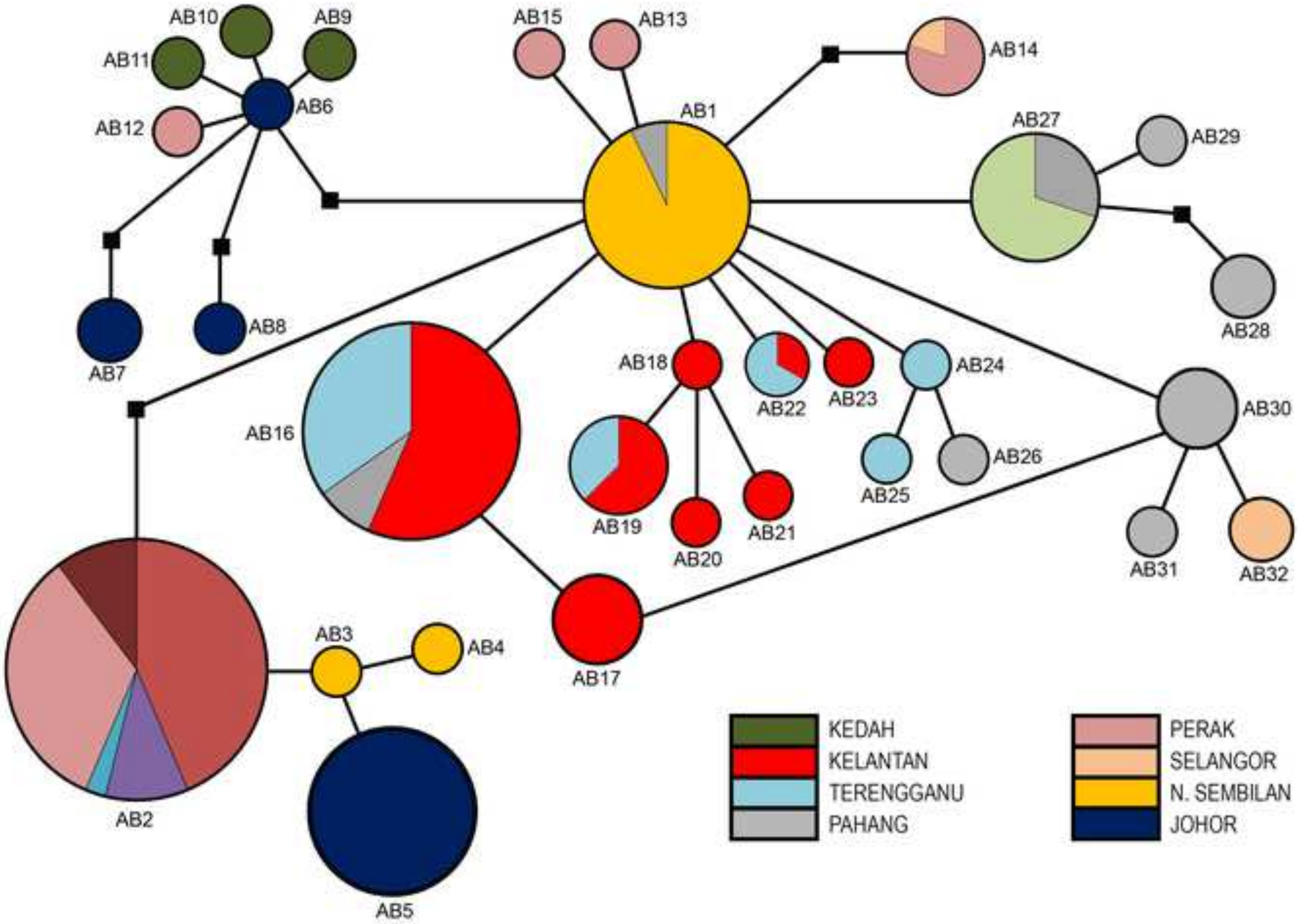

S1 Table

Click here to access/download
Supporting Information
S1 Table.pdf

S2 Table

Click here to access/download
Supporting Information
S2 Table.pdf

S3 Table

Click here to access/download
Supporting Information
S3 Table.pdf

Research Paper

Genetic diversity and phylogeographic patterns of the peacock jewel-damselfly, *Rhinocypha fenestrella* (Rambur, 1842) ~~(Odonata: Chlorocyphidae)~~

Mamat-Noorhidayah[1], Noor Azrizal-Wahid[2*], Van Lun Low[3], Norma-Rashid Yusoff[1]

[1] Institute of Biological Sciences, Faculty of Science, Universiti Malaya, Kuala Lumpur, Malaysia

[2] Department of Biology, Faculty of Science, Universiti Putra Malaysia, 43400 Serdang, Selangor, Malaysia

[3] Higher Institution Centre of Excellence (HICoE), Tropical Infectious Diseases Research & Education Centre (TIDREC), Universiti Malaya, 50603 Kuala Lumpur, Malaysia

*Corresponding author~~:~~

Email: noorazrizal@upm.edu.my

Abstract

The present study examined the genetic diversity and phylogeographic pattern of the peacock jewel-damselfly *Rhinocypha fenestrella* (Rambur, 1842) by sequencing the cytochrome C oxidase I (*cox1*) and *16S* rRNA gene regions from 147 individuals representing eight populations in Malaysia. A total of 26 and 10 unique haplotypes were revealed by the *cox1* and *16S* rRNA genes, respectively, and 32 haplotypes were recovered by the concatenated sequences of *cox1+16S*. Analyses indicated that haplotype AB2 was the most frequent and the most widespread haplotype in Malaysia, and the most recent common ancestor of the *R.*

Formatted: Font: Not Bold

Formatted: Font: 18 pt

Formatted: Font: Not Bold, Not Highlight

Formatted: Font: Not Bold

fenestrella may exist from the Negeri Sembilan as discovered from *cox1+16S* haplotype network analysis. Overall haplotype and nucleotide diversities of the concatenated sequences were H_d = 0.893767 and P_i = 0.002822, respectively, with great genetic differentiation (F_{ST} = 0.638769) and low gene flow (N_m = 0.14). Population from Pahang presented the highest genetic diversity (H_d = 0.888989, P_i = 0.0022215, N_h = 9), whereas Kedah population demonstrated the lowest diversity (H_d = 0.284211, P_i = 0.0003326, N_h = 4). The concatenated sequences of *cox1+16S* showed genetic divergence ranging from 0.09% to 0.97%, whereas the genetic divergence for *cox1* and *16S* rRNA genes were 0.16% to 1.63% and 0.01% to 0.75% respectively. This study provides for the first-time insights on the intraspecific genetic diversity, phylogeographic pattern and ancestral haplotype of *Rhinocypha fenestrella*.

Keywords: *cox1*, haplotype, Malaysia, population structure,~~*Rhinocypha fenestrella*~~ *16S*~~,~~

~~1.~~ Introduction

Formatted: Font: 18 pt

Rhinocypha (Cowley, 1937), from the family of Chlorocyphidae, is the most characteristic genus of dragonflies in tropical Asia. Rhinocyphae is of a very particular nature in their habitat [1] and certain species can adapt to and tolerate disturbed habitats ~~are more sensitive to habitat disturbance and are primarily found in undisturbed areas~~ [2]. This characteristic makes them good “thermometers” of environment quality, thus good study subjects for phylogeography [3]. *Rhinocypha fenestrella* (Rambur, 1842), syn, *Aristocypha fenestrella*, also known as a peacock jewel, is the most widespread species in the genus, and one of the most active species of the family, which occurs in Peninsular Malaysia, Thailand, Burma, Laos, Vietnam, and southern China [4]. It is one of several common mountain stream damselflies and is usually found in the primary forests [1].

~~Though~~ Malaysia is known to be one of the three mega-biodiversity countries in Southeast Asia, however, the phylogeographic pattern of dragonflies has been relatively scarce, especially for this particular species~~, but little has been published~~. Several phylogeographic studies have been published concerning odonates [5~~,6,~~7], however, until now, no comparable research related to odonate species in Malaysia has been conducted. Instead, the species with a widespread distribution that includes dragonflies normally will have complexes of multiple lineages or variations in the genetic diversity with the geographic region [8~~,9,10,11,~~12]. From a ~~view~~ point of zoogeography, the Rhinocyphae are of substantial importance, for instance, in the Malay Archipelago, each large island, mostly has its own group of endemic species [1].

A phylogeographic analysis is a powerful method of obtaining insights into the historical processes that have shaped the species' temporal distribution and genetic variation [13]. Recent phylogeographic research on widely distributed odonatan species has indicated different responses to historical climatic changes in space and time [14,15]. With the advances in molecular techniques, mitochondrial DNA has been identified as an excellent genetic marker of gene flow in matrilineal inheritance [16] and it is the most widely used marker to study the molecular ecology in animal taxa [17,18].

Particularly, cytochrome C oxidase subunit I (*cox1*) and 16S ribosomal RNA (*16S*) are known to be reliable genetic markers and the commonly applied markers in Odonata [7,19-~~,20,~~21]. In addition to nuclear markers, ~~these~~ mitochondrial markers were also provided well-resolved and supported trees from species to family level [22,23]. Given the high resolution of mitochondria-encoded *cox1* and *16S* genes reported in odonates [24], this study for the first time attempts to characterize the ~~intraspecific~~ genetic diversity and population ~~genetic~~ structure of *R. fenestrella*~~, for the first time~~ across its range in Malaysia.

~~2.~~ Materials and methods

Formatted: Font: 18 pt

~~2.1 Animal material~~Sample Collection~~s~~

A total of 147 *Rhinocypha fenestrella* individuals were collected from eight populations representing eight states in Peninsular Malaysia from the period of 2014 to 2015 with the permission of the Forestry Department Peninsular Malaysia (Permit Number: JH/100 Jld.7 (12)) (~~Table 1 and~~ Fig 1 and Table 1). The identification of *R. fenestrella* was performed according to morphological descriptions and taxonomic keys as described by Orr and Hamalainen (2003) [23], and through personal experience. Methods of sampling and preservation of Odonata were based on previously described standard methods [24]. Generally, samples were caught by using a sweep net and were dried preserved for morphological identification, while the legs were removed from each individual and stored in a vial containing ethanol for molecular works.

Fig 1. Map of sampling sites and haplotype distribution (AB1–AB15) of concatenated *cox1+16S* sequences for *Rhinocypha fenestrella* from eight sampling locations representing eight states of Peninsular Malaysia.

Table 1. Sampling localities and geographic position of sampling sites of *Rhinocypha fenestrella*.

**State**	**District**	**Locality**	**Geographic position**
N. Sembilan	Jelebu	Jeram Toi Waterfall	N 02 51' 52.7" E 102 00' 52.0"
Johor	Bekok	Sungai Bantang Waterfall	N 02 20' 46.7" E 103 09' 23.9"
Kedah	Banting	Lata Bayu Waterfall	N 05 43' 02.9" E 100 48' 50.9"
Perak	Tapah	Lata Iskandar Waterfall	N 04 19' 27.18" E 101 19' 31.36"
Kelantan	Machang	Jeram Linang Waterfall	N 05 44' 33.4" E 102 22' 26.2"
Terengganu	Kuala Berang	Sekayu Waterfall	N 04 57' 45.8" E 102 57' 11.9"
Pahang	Bentong	Chamang Waterfall	N 03 30' 34.2" E 101 51' 36.3"

Formatted: Font: 16 pt

Formatted: Font: Bold

Formatted: Line spacing: Multiple 1.15 li

Formatted: Font: Bold

Formatted: Font: 12 pt, Bold

Formatted: Font: Bold, Not Italic

Formatted: Line spacing: Multiple 1.15 li

Formatted: Line spacing: Double

Formatted Table

Formatted: Line spacing: Double

Formatted: Line spacing: Double

Formatted: Right: 0.06", Line spacing: Double

Formatted: Line spacing: Double

Formatted: Line spacing: Double

Formatted: Line spacing: Double

Formatted: Line spacing: Double

Formatted: Line spacing: Double

Formatted: Line spacing: Double

Selangor	Hulu Selangor	Sungai Sendat Waterfall	N 03 24' 14.4" E 101 41' 00.5"

Formatted: Line spacing: Double

~~2.2~~ DNA extraction and amplification

Formatted: Font: 16 pt

Genomic DNA was extracted from four to six legs of each ethanol-preserved ~~fresh~~ specimen using the i-genomic CTB DNA Extraction Mini Kit (iNtRON Biotechnology Inc., Seongnam, South Korea). The DNA amplifications of both *cox1* and *16S* genes were conducted using an Applied Biosystems Veriti 96-Well Thermal Cycler (Applied Biosystems Inc., Foster City, CA, USA) with the amplification protocol consisting of 300 sec at 94°C followed by 35 cycles of 50 sec at 94°C, 50 sec at 50°C and 50 sec at 72°C, and a final 7 min at 72°C. Primers for amplification of the *cox1* gene were 5'- GGT CAA CAA ATC ATA AAG ATA TTG G – 3' for forward primer [27] and 5'- GGA TGG CCA AAA AAT CAA AAT AAA TG –3' for reverse primer [28]. For the *16S* gene, ODO 12852 and ODO 13393 primer sets (forward primer, 5'- AGA AAC CGA CCT GGC TTA AA -3'; reverse primer, 5'- CGC CTG TTT ATC AAA AAC AT -3') were utilized [7]. Each PCR amplification was performed in a reaction mixture containing 50–100 ng of genomic DNA, 25 µl of NEXpro e-PCR 2X Master Mix (Genes Labs Inc., Gyeonggi-do, South Korea), and 10 pmol of each forward and reverse primer.

~~2.3~~ DNA purification, sequencing, and alignment

Formatted: Font: 16 pt

The amplified samples were then electrophoresed on 2% agarose gel pre-stained with SYBR Safe^{TM} (Invitrogen Corp., Carlsbad, CA, U.S.A.), and the PCR products were sent outsource to a commercial company (Apical Scientific Sdn. Bhd. Selangor, Malaysia) for DNA sequencing in both forward and reverse directions. The samples were sequenced using the BigDyeH Terminator 3.1 Sequencing Kit.

All sequences were assembled and edited using Molecular Evolutionary Genetics Analysis (MEGA) software Version 11.0 [29] and BioEdit 7.2 [30] and preliminarily aligned using CLUSTALX [31]. The aligned sequences were then subjected to a BLAST search (www.ncbi.nlm.nih.gov/blast/) for species validation. ~~The generated sequences that produce unique haplotypes in this study were deposited in the National Center for Biotechnology Information (NCBI) GenBank database under accession numbers KY678719–KY678744 for *cox1*, and KY678745–KY678754 for *16S* genes.~~

~~2.4~~ Genetic diversity and haplotype analyses

Formatted: Font: 16 pt

The aligned cox1 and 16S sequences at first were analysed respectively, then were concatenated to yield a total length for further analysis. ~~The aligned *cox1* and *16S* sequences comprised 614 bp and 534 bp, respectively, while the sequences of both *cox1* and *16S* were concatenated to yield a total length of 1148 bp.~~ Molecular characteristics such as the number of haplotypes (N_h), haplotype diversity (H_d), nucleotide diversity (P_i), the number of segregating sites (*S*), and average number of sequence differences (*K*) were determined with the program DNASP® (DNA Sequence Polymorphism) [32]. DNASP® was also used to perform neutrality tests including Tajima's *D* [33] and Fu's F_s [34].

The F_{ST} and N_m pairwise values were ~~determined~~ calculated to access genetic differentiation and gene flow among the *R. fenestrella* population and were calculated using DNASP®. The levels of genetic differentiation are defined as $F_{ST} > 0.25$ (great differentiation), $0.15 < F_{ST} < 0.25$ (moderate differentiation), and $F_{ST} < 0.15$ (negligible differentiation) according to the classification criteria by Wright (1978) [35]. The levels of gene flow are categorized as $N_m > 1$ (high gene flow), $0.25 < N_m < 0.99$ (intermediate gene flow), and $N_m < 0.25$ (low gene flow) [36]. Uncorrected pairwise distances (*p*-distance) ~~Uncorrected (*p*)~~

pairwise genetic distances were assessed using PAUP 4.0B10 to measure the genetic divergence of *R. fenestrella*.

Furthermore, to visualize the phylogeographic pattern between the populations, and to calculate the minimum number of mutational steps between the sequences, haplotype networks were constructed using TCS 1.13® [37] with a 95% parsimony criterion for both *cox1* and *16S* respectively, and concatenated *cox1* + *16S* genes sequences.

3. Results

3.1 Genetic diversity of *Rhinocypha fenestrella*

The final aligned sequence fragment lengths were 614 bp, 534 bp, and 1148 bp for *cox1*, *16S*, and concatenated *cox1+16S*, respectively. The generated sequences that produce unique haplotypes in this study were deposited in the National Center for Biotechnology Information (NCBI) GenBank database under accession numbers KY678719–KY678744 for *cox1*, and KY678745–KY678754 for *16S* genes.

The haplotype diversity (H_d) in a population for the *cox1* gene ranged from 0.19474 in the Kedah population to 0.888989 in the Pahang population. Nonetheless, for the *16S* gene, it ranged from 0.0000 found in three populations (Johor, Perak, and Selangor) to 0.28421 in the Kelantan population. In concatenated *cox1+16S* sequence analyses, the overall value of haplotype diversity was 0.893767, and nucleotide diversity (P_i) was 0.002822.

Kedah population showed the lowest diversity for both nucleotide and haplotype diversities (H_d = 0.284241, P_i = 0.000326326), while the highest haplotype diversity was shown by Pahang (H_d = 0.888989), and the highest nucleotide diversity was presented by the Kelantan population (P_i = 0.0023328) (Table 2). In total average data estimates, the *cox1* gene revealed

Formatted: Font: 18 pt

Formatted: Font: 16 pt

Formatted: Indent: First line: 0"

Formatted: Indent: First line: 0.5"

higher for both haplotype and nucleotide diversities (H_d = 0.8846~~3~~, P_i = 0.00510~~05~~) than did the *16S* gene (H_d = 0.1292, P_i = 0.00134).

Table 2: Genetic diversity indices and neutrality test based on *cox1*, *16S*, and concatenated *cox1+16S* sequences of *Rhinocypha fenestrella* from eight different populations in Peninsular Malaysia.

Markers	*Nh*	*Hd*	*Pi*	*S*	*K*	*D*	*Fs*
16S rRNA							
Johor	1	0.0000	0.0000	0	0	np	np
Kedah	2	0.1000	0.0002	1	0.1000	-1.1644	-0.879
Kelantan	4	0.2842	0.0010	4	0.4947	-1.9857*	-1.589
Negeri Sembilan	2	0.1053	0.0002	1	0.1053	-1.1648	-0.838
Pahang	3	0.1947	0.0054	15	2.7526	-1.2996	5.371
Perak	1	0.0000	0.0000	0	0.0000	np	np
Selangor	1	0.0000	0.0000	0	0.0000	np	np
Terengganu	4	0.2842	0.0031	15	1.5947	-2.4439*	1.627
**OVERALL**	**10**	**0.1292**	**0.0013**	**22**	**0.6850**	**-2.3808****	**-5.488**
cox1							
Johor	4	0.4316	0.001	3	0.4684	-1.1914	-1.713
Kedah	3	0.1947	0.000	2	0.2000	-1.5128	-1.863
Kelantan	4	0.7263	0.003	5	2.0158	1.3167	1.812
Negeri Sembilan	3	0.4328	0.0007	2	0.4561	-0.4849	-0.421
Pahang	9	0.8889	0.0040	9	2.4641	-0.2066	-2.838
Perak	5	0.5579	0.0013	5	0.7947	-1.3344	-1.711
Selangor	3	0.5636	0.0013	3	0.8000	-0.7494	0.158
Terengganu	6	0.7778	0.0028	6	1.7193	0.0049	-0.780
**OVERALL**	**26**	**0.8846**	**0.0051**	**28**	**3.0981**	**-1.1123**	**-9.824**
cox1+16S							
Johor	4	0.4316	0.0004	3	0.4684	-1.1914	-1.713
Kedah	4	0.2842	0.0003	3	0.3000	-1.7233	-2.749
Kelantan	8	0.8211	0.0023	10	2.6105	-0.5605	-1.297
Negeri Sembilan	4	0.5088	0.0005	3	0.5614	-0.9407	-1.355
Pahang	9	0.8889	0.0021	9	2.4641	-0.2066	-2.838
Perak	5	0.5579	0.0007	5	0.7947	-1.3344	-1.711
Selangor	3	0.5636	0.0007	3	0.8000	-0.7494	0.158
Terengganu	6	0.7778	0.0017	8	1.9298	-0.5366	-0.460
**OVERALL**	**32**	**0.8937**	**0.0028**	**37**	**3.2341**	**-1.5732**	**-16.523**

~~3.2~~ Haplotype variation and distribution

A set of aligned sequences of 147 taxa of the *cox1* revealed 26 haplotypes (A1 – A26). For *cox1*, the most prevalent haplotype was A2 (*n* = 40) and was found ~~to exist~~ in five populations. The second most-frequent haplotypes were A1, A4, and A14 (*n* = 15) while the least prevalent haplotypes presented as singleton were A3, A5, A7 – A11, A18 – A20, A23, and A25. Notably, based on the star-like pattern of the haplotype network (Fig 2), the haplotypes A2 – A26 from the *cox1* gene were considered to be originated from haplotype A1~~, as their common ancestor~~.

Fig 2. Median joining haplotype network of *Rhinocypha fenestrella* of *cox1* sequences isolated from eight different states in Peninsular Malaysia. Each circle represents a haplotype variation, and the size of a circle is proportional to the number of sequences assigned to that haplotype. Circles of the same colour represent haplotypes from the same population. A small black square represents median vectors.

For the *16S* gene, the sequences revealed 10 haplotypes (B1 – B10). The most prevalent haplotype was B1 (*n* = 140) while all other haplotypes except B8 (*n* = 2), appeared as singleton haplotypes. Haplotype B1 was ~~also the most widespread and can be~~ found in all populations. The haplotype network of *16S* gene showed a star-like pattern suggesting haplotype B1 as a common ancestor of all the others ~~while other haplotypes (B2 – B10) originated from haplotype B1~~ (Fig 3). Nevertheless, a median-joining network showed no obvious geographical pattern in haplotype distribution.

Fig 3. Median joining haplotype network of *Rhinocypha fenestrella* of *16S* sequences isolated from eight different states in Peninsular Malaysia. Each circle represents a haplotype variation, and the size of a circle is proportional to the number of sequences assigned to that haplotype. Circles of the same colour represent haplotypes from the same population. A small black square represents median vectors.

Formatted: Font: 16 pt

Formatted: Font: Bold

Formatted: Font: (Default) +Body (Calibri), 11 pt, Font color: Auto

Formatted: Space After: 10 pt, Line spacing: 1.5 lines

Formatted: Font: Bold

The concatenated *cox1+16S* sequences revealed 32 haplotypes (AB1 – AB32). Haplotype AB2 was the most frequent and the most widespread haplotype based on its prevalence in Malaysia, while there were 18 singleton haplotypes (AB3, AB4, AB6, AB8 – AB12, AB15, AB18, AB20, AB21, AB 23 – AB26, AB29, and AB31 (Table 3~~4~~). Furthermore, the Kelantan population presented as the most diverse population that had the highest number of haplotypes (N_h = 30) while the least number of haplotypes was presented in the Selangor population (N_h = 11). Haplotype AB1 appeared to be as a common ancestor ~~to~~ of the other haplotypes as indicated by the star-like pattern of concatenated *cox1+16S* haplotype network (Fig 4). Consequently, ~~in terms of location-based~~based of sampling sites, this study revealed that a recent common ancestor of the *R. fenestrella* species in Malaysia existed from the Negeri Sembilan population that constituted the most numbers of haplotype AB1.

Fig 4. Median joining haplotype network of *Rhinocypha fenestrella* of concatenated *cox1+16S* sequences isolated from eight different states in Peninsular Malaysia. Each circle represents a haplotype variation, and the size of a circle is proportional to the number of sequences assigned to that haplotype. Circles of the same colour represent haplotypes from the same population. A small black square represents median vectors.

Formatted: Indent: First line: 0"

Formatted: Font: Bold

Formatted: Indent: First line: 0"

Table 3. Haplotype distribution of *Rhinocypha fenestrella* (*n* = 147) from Peninsular Malaysia based on the concatenated *cox1+16S* sequences corresponding to the eight sampling sites.

Sampling sites	*n*	Haplotype *cox1 + 16S rRNA*																															
		AB1	AB2	AB3	AB4	AB5	AB6	AB7	AB8	AB9	AB10	AB11	AB12	AB13	AB14	AB15	AB16	AB17	AB18	AB19	AB20	AB21	AB22	AB23	AB24	AB25	AB26	AB27	AB28	AB29	AB30	AB31	AB32
N. Sembilan	19	13	4	1	1	-	-	-	-	-	-	-	-	-	-	-	-	-	-	-	-	-	-	-	-	-	-	-	-	-	-	-	-
Johor	20	-	-	-	-	15	1	3	1	-	-	-	-	-	-	-	-	-	-	-	-	-	-	-	-	-	-	-	-	-	-	-	-
Kedah	20	-	17	-	-	-	-	-	-	1	1	1	-	-	-	-	-	-	-	-	-	-	-	-	-	-	-	-	-	-	-	-	-
Perak	20	-	13	-	-	-	-	-	-	-	-	-	1	1	4	1	-	-	-	-	-	-	-	-	-	-	-	-	-	-	-	-	-
Kelantan	20	-	-	-	-	-	-	-	-	-	-	-	-	-	-	-	13	7	1	5	1	1	1	1	-	-	-	-	-	-	-	-	-
Terengganu	19	-	4	-	-	-	-	-	-	-	-	-	-	-	-	-	8	-	-	3	-	-	2	-	1	1	-	-	-	-	-	-	-
Pahang	18	1	1	-	-	-	-	-	-	-	-	-	-	-	-	-	2	-	-	-	-	-	-	-	-	-	1	3	3	1	5	1	-
Selangor	11	-	-	-	-	-	-	-	-	-	-	-	-	1	-	-	-	-	-	-	-	-	-	-	-	-	-	7	-	-	-	-	3
**TOTAL**	**147**	**14**	**39**	**1**	**1**	**15**	**1**	**3**	**1**	**1**	**1**	**1**	**1**	**2**	**4**	**1**	**23**	**7**	**1**	**8**	**1**	**1**	**3**	**1**	**1**	**1**	**1**	**10**	**3**	**1**	**5**	**1**	**3**

n = number of odonates

~~3.3~~ Haplotype genetic divergence

~~Uncorrected genetic divergence~~The *p*-distance between *R. fenestrella* haplotypes for *cox1* ranged from 0.16% – 1.63% with the highest value showed pairwise between haplotypes A5 with A15, A17, A19, and between haplotypes A7 with A15, A17, A19 (~~Appendix A – see supplementary file~~S1 Table). On the other hand, the genetic divergence of *16S* haplotypes ranged from 0.01% – 0.75% with the highest value presented pairwise between haplotypes B5 and B7 (~~Appendix B – see supplementary file~~S2 Table). For the concatenated *cox1+16S*, the genetic divergence ranged from 0.09% to 0.97% (S3 Table~~Table 5~~).

In concatenated *cox1+16S* sequences, the neutrality test of Fu's F_s showed negative values in all populations with an exception for Selangor. Additionally, Tajima's *D* tests for all populations were indicated by negative values and were not statistically significant. The overall F_{ST} was 0.6387~~69~~ and N_m was 0.14, suggesting that the genetic differentiation was great while there was a low gene flow among the populations of *R. fenestrella* in Malaysia. Based on Table 4, the highest F_{ST} value (0.0100) appeared between Perak and Selangor populations while the lowest value (0.0010) was between the populations of Negeri Sembilan and Kedah, Negeri Sembilan and Perak, Kedah, and Perak, Kedah and Pahang, and Kedah and Selangor.

Table 4. Pairwise genetic differentiation (F_{ST}) among *Rhinocypha fenestrella* populations in eight distinct populations in Peninsular Malaysia.

Population	S1	S2	S3	S4	S5	S6	S7	S8
**N. Sembilan**	-	-	-	-	-	-	-	-
**Johor**	0.006	0.005	-	-	-	-	-	-
**Kedah**	0.001	0.006	0.001	-	-	-	-	-
**Perak**	0.001	0.007	0.002	0.002	-	-	-	-
**Kelantan**	0.003	0.007	0.002	0.002	0.002	-	-	-
**Terengganu**	0.002	0.007	0.001	0.002	0.003	0.003	-	-
**Pahang**	0.002	0.006	0.001	0.010	0.003	0.003	0.002	-

Formatted: Font: 16 pt
Formatted: Header distance from edge: 0.49", Footer distance from edge: 0.49"
Formatted: Font: 12 pt
Formatted: Font: 12 pt, Bold
Formatted Table
Formatted: Line spacing: 1.5 lines
Formatted: Line spacing: 1.5 lines
Formatted: Line spacing: 1.5 lines
Formatted: Line spacing: 1.5 lines
Formatted: Line spacing: 1.5 lines
Formatted: Line spacing: 1.5 lines
Formatted: Line spacing: 1.5 lines
Formatted: Font: (Default) +Headings CS (Times New Roman), Font color: Auto
Formatted: Indent: Left: -0.08", Right: -0.07", Line spacing: Multiple 1.15 li

~~4.~~ Discussion

Formatted: Font: 18 pt

~~4.1~~ Genetic diversity of Malaysian *Rhinocypha fenestrella*

Formatted: Font: 16 pt

Throughout the last five decades, the understanding of the ecology and evolution of odonates has increased dramatically. ~~w~~R~~hile r~~ecent advances in molecular techniques have inspired several phylogeographical studies of ~~odonata~~ Odonata ~~by~~ using genetic data [24,38,39]. As is known, most of the odonates ~~are extremely mobile and~~ have varying levels of dispersal abilities [40] that could influence the genetic diversity and phylogeographic structures of the populations. A less-mobile species may be expected to show some evidence of haplotype clustering according to geographic region [41]. ~~–~~Therefore, in this study, we defined the intraspecific genetic diversity, phylogeographical patterns, and mitochondrial variations of the *Rhinocypha fenestrella* using *cox1* and *16S* genes. ~~for the first time across eight populations within Peninsular Malaysia.~~

The obtained sequences for *cox1* and *16S* genes from eight populations ranged from 1.5 – 4.6% of variability among all studied individuals reflected in 28 variable nucleotide sites in 614 sequenced base pairs from *cox1* gene, and eight variable nucleotides in 534 base pairs from *16S* gene. The base composition of both *cox1* and *16S* gene sequences showed a significant A+T nucleotide bias which is consistent with insect mitochondrial genes [38,42,43].

~~Based on~~ In the present study, overall genetic diversity (H_d = 0.8937; P_i = 0.0028) of Malaysian *R. fenestrella* based on the concatenated *cox1+16S* sequences was slightly lower if compared to the study that did on the damselfly *Matrona basilaris* populations from mainland China [44]. The study reported higher values of haplotype diversity (H_d = 0.9750) and nucleotide diversity (P_i = 0.0049) by using *cox1* sequences. However, when compared with the study on *Pantala flavescens* (mean H_d = 0.8100; mean P_i = 0.0060) by using *Cytb* marker [45],

the genetic diversity of this study was found to be slightly higher. Nevertheless, the genetic diversity of *R. fenestrella* in Malaysia can be considered as high when compared to other damselflies species from the same sub-order *Psolodesmus mandarinus* (MacLahlan, 1870) from Taiwan that reported haplotype diversity as 0.43 and nucleotide diversity as 0.0017 based on *cox1+16S* sequences [46].

4.2 Haplotype variation and distribution of *Rhinocypha fenestrella*

Formatted: Font: 16 pt

In this study, the number of haplotypes detected was considered high with 26 haplotypes were revealed by *cox1* and 10 haplotypes by *16S*. The high numbers of haplotypes may indicate a high degree of *cox1* and *16S* gene polymorphisms in *R. fenestrella* samples in Peninsular Malaysia. In fact, the polymorphism that occurred in Odonata were widely reported in other studies [47,48]. Moreover, this study demonstrated extremely high numbers of haplotypes when comparing to the study by Lin et al. (2012) [46] that reported only 14 haplotypes variation in the damselfly population of *Psolodesmus mandarinus* from Taiwan, while the dragonfly population from Scandinavia and Finland only revealed of four haplotype variations [49].

Formatted: Not Highlight

Formatted: Not Highlight

Formatted: Not Highlight

Formatted: Not Highlight

In this study, the number of haplotypes detected was considered low because only ten haplotypes were revealed by *cox1* and eight haplotypes by *16S*, with the presence of several singleton haplotypes that were restricted to particular areas. The low numbers of haplotypes may indicate a low degree of *cox1* and *16S* gene polymorphisms in *R. fenestrella* samples in Peninsular Malaysia. However, this study demonstrated high numbers of haplotype based on concatenated data that produced 32 haplotype variations. As compared to other studies, Lin et al. (2012) [45] reported only 14 haplotypes variation in the damselfly population of *Psolodesmus mandarinus* from Taiwan while only four haplotypes were discovered in the dragonfly population from Scandinavia and Finland [46].

The haplotype AB2 appeared as the most dominant haplotype, while the limited geographical distribution of some of the haplotypes including those found as singleton suggests the existence of genetic differentiation within the populations. In this study, haplotype AB1 was suggested as the common ancestor due to star-like radiation in the haplotype network pattern. This haplotype probably had eventually evolved over time into the numerous haplotypes (AB2–AB15) in order to adapt to the habitat and demographic changes and consequently distributed across states in Peninsular Malaysia. Likewise, according to location-based, the most recent common ancestor for *R. fenestrella* in Malaysia may be derived from Negeri Sembilan as this state is constituted by high frequencies of the common ancestor AB1 haplotypes.

The median-joining network also revealed a close relationship among haplotypes, suggesting that *R. fenestrella* populations in Malaysia shared a recent history without long-term genetic isolation. Nevertheless, the ancestral haplotype AB1 of concatenated *cox1+16S* dataset was having a limited distribution with restricted geographic division. This indicates that geographic barriers and climatic factors could also have little influence on the dispersion of this damselflies into different habitats in Malaysia besides their less ability to disperse due to weak flyers.

~~4.3~~ Genetic distance and differentiation

Formatted: Font: 16 pt

In our dataset, the highest genetic distance value based on *cox1* gene was 0.16%, while *16S* gene was 0.75%. The data shows a higher genetic distance among Malaysian *R. fenestrella* as compared to the populations of *Rhinocypha taiwana* (Wang & Chang, 2013), *Rhinocypha uenoi* (Asahina, 1964), and *Rhinocypha drusilla* (Needham, 1930) from China where the highest genetic distances based on *cox1* gene were 0.00%, 0.00% and 0.50%, respectively [50]. However, the genetic distance of Malaysian *R. fenestralla* was relatively lower if compared to

other dragonfly populations of *Trithemis stictica* (Burmeister, 1839) in Namibia [9] and *Nannophya pygmaea* (Rambur, 1842) in Malaysia [51] that presented the genetic distance of *cox1* gene ranging from 0.00% - 9.00% and 0.00% - 12.00%, respectively.

Zygopterans are known as weaker fliers ~~by~~ since many species do not diffusing outside several kilometres [52,53]. *Rhinocypha fenestrella* is known as a weak flyer and is unlikely to migrate over large-scale regions, which likely contributes to its overall high genetic differentiation to happen in and between the populations. ~~Therefore~~However, in the present study, it is somewhat~~how not~~ surprising that genetic differentiation happened in the species *R. fenestrella* in which their populations were separated up to more than 500 km. ~~In contrast~~Similarly,~~,~~ the migratory behaviour of odonate could homogenize genetic differentiation among populations by the exchange of individuals and genes among populations~~. For example, low~~ with high genetic differentiation has been observed in the population of migratory dragonflies *Libellula quadrimaculata* (Linnaeus, 1758) [38] and *Pantala flavescens~~flavensens~~* (Fabricius, 1798) [54] respectively ~~where they~~which are known as strong fliers ~~that~~ than the damselflies.

~~Natural d~~Dispersal ability and long-distance migration are the most important factors contributing to a high level of gene flow and consequent slowing or ~~limitation~~ limitating of geographic differentiation [55,56]. A less-mobile species may be expected to show some evidence of haplotype clustering according to geographic region due to less ability to disperse and therefore resulted in genetic isolation by distance. In this study, calculated F_{st} value shows high genetic differentiation ~~occurred~~ in the populations of *R. fenestrella* in Malaysia. This high value is consequently supported by the low rate of gene flow found amongst the *R. fenestrella* populations in Malaysia as well as between the different ~~regions~~sites.

When all eight populations were regarded as a whole, Tajima's *D* statistic was statistically ~~negative and~~ not significant. The results showed that these populations were in a

Formatted: Font: Not Italic

stable state with no demographic expansion and no recent bottleneck. Whereas, in the Selangor population, Fu’s F_S statistic values were negative with *p*-values being significant ($p < 0.02$) which shows this population had experienced a recent population expansion. Our results suggested that *R. fenestrella* population in Selangor could successfully colonize and adapt to new habitats and were able to disperse randomly and exchange genes with local populations. Maintaining genetic diversity is noteworthy as it related to population viability [57~~-,55,~~59] and also to the transformative potential of a species to react to the environmental changes [60,61].

Overall, this study found strong evidence for intraspecific patterns of ~~molecular~~ haplotype variation among populations of *R. fenestrella* in Malaysia though the weak dispersal abilities of the species. The study also revealed high genetic differentiation within the populations ~~while~~ and a low rate of gene flow among the geographically difference populations of Malaysian *R. fenestrella*. Moreover, a high haplotype number was observed in *R. fenestrella* population, but no genetic isolation was found within the populations of the sampling sites. Although many phylogeographical mechanisms have been proposed using damselflies as model organisms, more detailed sampling and a larger variety of ecological investigations are required to promote better understanding. Therefore, this study contributed new insight for an advanced understanding of the evolution and phylogeography of damselfly. ~~Moreover, a high haplotype number was observed in *R. fenestrella* population but with no genetic isolation by populations. Although many phylogeographical mechanisms have been proposed using damselflies as model organisms, but more detailed sampling and a larger variety of ecological investigations are required to promote understanding of damselfly and advance their evolution and phylogeography~~Additional sequence data from ~~this particular species gene from another continent~~ another regions (Thailand, Burma, Laos, Vietnam, and southern China), which is considered ~~as~~ a gap of knowledge due to the lack of genetic data on this particular species, may prove useful in detecting phylogenetic relationships~~,~~ and

phylogeography patterns, and revealing common ancestors of these populations between the other regions and continents. within species between the continents.

Competing Interests

Formatted: Font: 18 pt, Not Highlight

Formatted: Font: 18 pt

The authors have declared that no competing interests exist.

Formatted: Not Highlight

Acknowledgements

Formatted: Font: 18 pt

This study was supported by a research grant from the University of Malaya, PG065 – 2015 and ST061-2022. The authors would like to thank Mr. Mohaiyidin Mohamed and Mr. Mohd Fauzi Abd Hamid for their assistance in the fieldwork.

References

Formatted: Font: 18 pt

1. Lieftinck MA. Two interesting new insular *Rhinocypha* from Malaysia (Odon.). Tijdschrift Voor Entomologie. 1947; 88: 215–224.
2. Mamat N, Abu A, Yusoff NR. Classification and morphology of *Rhinocypha* spp. (Odonata): A comprehensive taxonomic study within the females. Zoological Studies. 2021; 26(60): e47.
3. Córdoba–Aguilar A. Introduction. In: Córdoba–Aguilar A, editor. Dragonflies and damselflies: study models in ecological and evolutionary research. Oxford (UK): Oxford University Press; 2008. p. 1–3.
4. Sharma G. *Rhinocypha fenestrella*. The IUCN Red List of Threatened Species. 2010.
5. Kahilainen A, Puurtinen M, Kotiaho JS. Conservation implications of species–genetic diversity correlations. Global Ecology and Conservation. 2014; 2: 315–323.

6. Monroe E, Britten B. Conservation in Hine's sight: the conservation genetics of the federally endangered Hine's emerald dragonfly, Somatochlora hineana. Journal of Insect Conservation. 2014; 18: 353-363.
7. Dijkstra KDB, Kalkman VJ, Dow RA, Stokvis FR, Tol JV. Redefining the damselfly families: the first comprehensive molecular phylogeny of Zygoptera (Odonata). Systematic Entomology. 2014; 39(1): 68–96.
8. Angulo A, Icochea J. Cryptic species complexes, widespread species and conservation: lessons from Amazonian frogs of the *Leptodactylus marmoratus* group (Anura: Leptodactylidae). Systematics and Biodiversity. 2010; 8: 357–370.
9. Damm S, Schierwater B, Hadrys H. An integrative approach to species discovery in odonates: from character-based DNA barcoding to ecology. Molecular Ecology. 2010; 19: 3881–3893.
10. Low VL, Takaoka H, Adler PH, Ya'cob Z, Norma-Rashid Y, Chen CD, Sofian-Azirun M. A multi-locus approach resolves the phylogenetic relationships of the *Simulium asakoae* and *Simulium ceylonicum* species groups (Diptera: Simuliidae) in Malaysia: evidence for distinct evolutionary lineages. Medical and Veterinary Entomology. 2015a; 29: 330–337.
11. Low VL, Tay ST, Kho KL, Koh FX, Tan TK, Lim YAL, Panchadcharam C, Ong BL, Norma-Rashid Y, Sofian-Azirun M. Molecular characterisation of the tick *Rhipicephalus microplus* in Malaysia: new insights into the cryptic diversity and distinct genetic assemblages throughout the world. Parasites and Vectors. 2015b; 8: 341.
12. Low VL, Takaoka H, Pramual P, Adler PH, Ya'cob Z, Huang YT, Pham XD, Ramli R, Chen CD, Wannaket A, Sofian-Azirun M. Delineating taxonomic boundaries in the largest species complex of black flies (Simuliidae) in the Oriental Region. Scientific Reports. 2016a; 6: 20346.

13. Avise JC. *Phylogeography*: The History and Formation of Species. Harvard University Press, Cambridge. 2000.
14. Kohli M, Letsch H, Greve C, Béthoux O, Deregnaucourt I, Liu S, Zhou X, Donath A, Mayer C, Podsiadlowski L, Gunkel S, Machida R, Niehuis O, Rust J, Wappler T, Yu X, Misof B, Ware J. Evolutionary history and divergence times of Odonata (dragonflies and damselflies) revealed through transcriptomics. iScience. 2021; 24(11): 103324.
15. Cancellario T, Miranda R, Baquero E, Fontaneto D, Martínez A, Mammola S. Climate change will redefine taxonomic, functional, and phylogenetic diversity of Odonata in space and time. npj Biodiversity. 2022; 1(1): 1-14.
16. Jisha KEK, Sebastian CD. Genetic variation and phylogeny assessment of *Aciagrion Occidentale* (Odonata: Coenagrionidae) using mitochondrial cytochrome oxidase subunit I gene. International Journal of Science and Research. 2013; 4(4): 1121–1123.
17. Norris DE. Genetic markers for study of the anopheline vectors of human malaria. Int Journal of Parasitology. 2002; 32: 1607–1615.
18. Pramual P, Kuvangkadilok C, Baimai V, Walton C. Phylogeography of the black fly *Simulium tani* (Diptera: Simuliidae) from Thailand as inferred from mtDNA sequences. Molecular Ecology. 2005; 14: 3989–4001.
19. Artiss T, Schultz TR, Polhemus DA, Simon C. Molecular phylogenetic analysis of dragonfly genera *Libellula*, *Ladona* and *Plathemis* (Odonata: Libelluinidae) based on mitochondrial cytochrome oxidase I and 16S rRNA sequence data. Molecular Phylogenetics and Evolution. 2001; 18: 348–361.
20. Yong HS, Lim P-E, Tan J, Ng YF, Eamsobhana P, Suana IW. Molecular phylogeny of *Orthetrum* dragonflies reveals cryptic species of *Orthetrum pruinosum*. Scientific Reports. 2014; 4: 5553.

21. Kim MJ, Jung KS, Park, NS, Wan X, Kim KG, Jun J, Jun J, Yoon TJ, Bae YJ, Lee SM, Kim I. Molecular phylogeny of the higher taxa of Odonata (Insecta) inferred from COI, 16S rRNA, 28S rRNA, and EF1-α sequences. Entomological Research. 2014; 44(2): 65–79.
22. Gaines CA, Hare MP, Beck SE, Rosenbaum HC. Nuclear markers confirm taxonomic status and relationships among highly endangered and closely related right whale species. Proceedings in Biological Sciences. 2005; 272(1562): 533–542.
23. Ballare EF, Ware JL. Dragons fly, biologists classify: an overview of molecular odonate studies, and our evolutionary understanding of dragonfly and damselfly (Insecta: Odonata) behavior. International Journal of Odonatology. 2011; 14: 137–147.
24. Ožana S, Dolný A, Páne T. Nuclear copies of mitochondrial DNA as a potential problem for phylogenetic and population genetic studies of Odonata. Systematic Entomology. 2022; 47(4): 591–602.
25. Orr AG, Hamalainen M. *A Guide to the Dragonflies of Borneo: their Identification and Biology*. Natural History Publications, Borneo. 2003.
26. Orr D. The constitution of nature. Conservation Biology. 2003; 17: 1478–1484.
27. Folmer O, Black M, Hoeh W, Lutz R, Vrijenhoek R. DNA primers for amplification of mitochondrial cytochrome C oxidase subunit I from diverse metazoan invertebrates. Molecular Marine Biology and Biotechnology. 1994; 3(5): 294–299.
28. Barrett RDH, Hebert PDN. Identifying spiders through DNA barcodes. Canadian Journal of Zoology. 2005; 83(3): 481–491.
29. Kumar S, Stecher G, Tamura K. MEGA7: Molecular Evolutionary Genetics Analysis Version 7.0 for Bigger Datasets. Molecular Biology and Evolution. 2016; 33(7): 1870–1874.

30. Hall TA. BioEdit: a user-friendly biological sequence alignment editor and analysis program for Windows 95/98/NT. Nucleic Acids Symposium Series. 1999; 41(2): 95–98.
31. Thompson JD, Gibson TJ, Plewniak F, Jeanmougin F, Higgins DG. The ClustalX Windows interface: flexible strategies for multiple sequence alignment aided by quality analysis tools. Nucleic Acids Research. 1997; 24: 4876–4882.
32. Librado P, Rozas J. DNASP v5: A software for comprehensive analysis of DNA polymorphism data. Bioinformatics. 2009; 25: 1451–1452.
33. Tajima F. Statistical method for testing the neutral mutation hypothesis by DNA polymorphism. Genetics. 1989; 123: 585–595.
34. Fu YX. Statistical tests of neutrality of mutations against population growth, hitchhiking and background selection. Genetics. 1997; 147: 915–925.
35. Wright S. *Evolution and Genetics of Populations*. 1978. University of Chicago, Chicago.
36. Slatkin M. Estimating levels of gene flow in natural populations. Genetics. 1981; 99: 323–335.
37. Clement M, Posada D, Crandall KA. TCS: a computer program to estimate gene genealogies. Molecular Ecology. 2000; 9(10): 1657–1659.
38. Artiss T. Phylogeography of a facultatively migratory dragonfly, *Libellula quadrimaculata* (Odonata: Anisoptera). Hydrobiologia. 2004; 515: 225–234.
39. Simonsen T, Olsen K, Djernaes M. The African-Iberian connection in Odonata: mtDNA and ncDNA based phylogeography of Aeshna cyanea (Müller, 1764) (Odonata: Aeshnidae) in Western Palaearctic. Arthropod Systematics and Phylogeny. 2020; 78(2): 309-320.
40. Rundle SD, Bilton DT, Foggo A. Body Size: The Structure and Function of Aquatic Ecosystems. In: Hildrew AG, Raffaelli DG, Edmonds-Brown R. editors. By wind, wings

or water: body size, dispersal and range size in aquatic invertebrates. Cambridge (UK): Cambridge University Press; 2007. p. 186–209.

41. Shannon J, McCauley SJ, Davis CJ, Werner EE, Robeson MS. Dispersal, niche breadth and population extinction: colonization ratios predict range size in North American dragonflies. Journal of Animal Ecology. 2014; 83: 858–865.

42. Frati F, Simon C, Sullivan J, Swofford DL. Evolution of the mitochondrial cytochrome oxidase II gene in Collembola. Journal of Molecular Evolution. 1997; 44(2): 145–158.

43. Chippindale PT, Whitmore DH, Davé VK, Valencia TG, Robinson JV. Effective procedures for the extraction, amplification and sequencing of Odonate DNA. Odonatologica. 1998; 27: 415–424.

44. Xue J, Zhang H, Ning X, Bu W, Yu X. Evolutionary history of a beautiful damselfly, Matrona basilaris, revealed by phylogeographic analyses: the first study of an odonate species in mainland China. Heredity (Edinb). 2019; 122(5): 570-581.

45. Cao L, Wu K. Genetic diversity and demographic history of globe skimmers (Odonata: Libellulidae) in China based on microsatellite and mitochondrial DNA markers. Scientific Reports. 2019; 9: 8619

46. Lin SC, Chen YF, Shieh SH, Yang PS. Patterns of mitochondrial and wing morphological differentiation in Taiwanese populations of *Psolodesmus mandarinus* McLachlan (Zygoptera: Calopterygidae). Odonatologica. 2012; 41(2): 109–121.

47. Sánchez-Guillén R, Ceccarelli S, Villalobos F, Neupane S, Rivas-Torres A, Sanmartín-Villar I, Wellenreuther M, Bybee SM, Velásquez-Vélez MI, Realpe E, Chávez-Ríos JR, Dumont HJ, Cordero-Rivera A. The evolutionary history of colour polymorphism in Ischnura damselflies (Odonata: Coenagrionidae). Odonatologica. 2020; 49: 333–370.

48. Joshi S, van der Poorten N, Sumanapala A, Nielsen E, Patel J, Nielsen B. New records of polymorphism in Asian libellulid dragonflies (Insecta: Odonata). International Journal of Odonatology. 2020; 23(4): 337–356.
49. Kohli MK, Sahlén G, Kuhn WR, Ware JL. Extremely low genetic diversity in a circumpolar dragonfly species, *Somatochlora sahlbergi* (Insecta: Odonata: Anisoptera). Scientific Reports. 2018; 8(1): 15114.
50. Wang LJ, Cherng JJ, Chang YJ, Jiang JL. Description of *Rhinocypha taiwana* sp. nov. from Taiwan, with a preliminary molecular phylogenetic analysis of the *Rhinocypha drusilla*-group (Odonata: Chlorocyphidae). International Journal of Odonatology. 2013; 16: 93–107.
51. Low VL, Sofian-Azirun M, Norma-Rashid Y. Playing hide-and-seek with the tiny dragonfly: DNA barcoding discriminates multiple lineages of *Nannophya pygmaea* in Asia. Journal of Insect Conservation. 2016; 20: 339–343.
52. Purse BV, Hopkins GW, Day KJ, Thompson DJ. Dispersal characteristics and management of a rare damselfly. Journal of Applied Ecology. 2003; 40: 716–728.
53. Watts PC, Rouquette JR, Saccheri IJ, Kemp SJ, Thompson DJ. Molecular and ecological evidence for small-scale isolation by distance in an endangered damselfly, *Coenagrion mercuriale*. Molecular Ecology. 2004; 13: 2931–2945.
54. Low VL, Norma-Rashid Y, Yusoff A, Vinnie-Siow WY, Prakash BK, Tan TK, Noorhidayah M, Chen CD, Sofian-Azirun M. Pleistocene demographic expansion and high gene flow in the Globe Skimmer dragonfly *Pantala flavescens* Fabricius (Odonata: Libellulidae) in Peninsular Malaysia. Zoologischer Anzeiger. 2017; 266: 23–27.
55. Rowe RJ. Conservation of Odonata in the South Pacific and Australasia. International Journal of Odonatology. 2004; 7(2): 139–147.

56. Ware J, Kohli MK, Mendoza CM, Troast D, Jinguji H, Hobson KA, Sahlén G, Anderson RC, Suhling F. Evidence for widespread gene flow and migration in the Globe Skimmer dragonfly *Pantala flavescens*. International Journal of Odonatology. 2022; 25: 43–55.
57. Saccheri IJ, Kuussaari M, Kankare M, Vikman P, Fortelius W, Hanski I. Inbreeding and extinction in a butterfly metapopulation. Nature. 1998; 392: 491–494.
58. Madsen T, Shine R, Olsson M, Wittzell H. Conservation biology – restoration of an inbred adder population. Nature. 1999; 402: 34–35.
59. Spielman D, Brook BW, Briscoe BW, Frankham R. Does inbreeding and a loss of genetic diversity decrease disease resistance?. Conservation Genetics. 2004; 5: 439–448.
60. Frankham R, Ballou JD, Briscoe DA. *Introduction to Conservation Genetics*. Cambridge University Press, Cambridge. 2002.
61. Reed DH, Frankham R. Fitness is correlated with genetic diversity. Conservation Biology. 2003; 17: 1–9.

Supporting Information

S1 Table. Percentage (%) of uncorrected "*p*" distance matrix among the 26 representative *cox1* haplotypes of *Rhinocypha fenestrella* in Malaysia.

S2 Table. Percentage (%) of uncorrected "*p*" distance matrix among the 10 representative *16S rRNA* haplotypes of *Rhinocypha fenestrella* in Malaysia.

S3 Table. Percentage (%) of uncorrected "*p*" distance matrix among the 32 representatives for the concatenated *cox1+16S* haplotypes of *Rhinocypha fenestrella* in Malaysia.

Author Contributions

Conceptualization: Norma-Rashid Yusoff, Mamat-Noorhidayah

Data curation: Mamat-Noorhidayah

Formal analysis: Mamat-Noorhidayah, Noor Azrizal-Wahid, Van Lun Low,

Funding acquisition: Norma-Rashid Yusoff, Mamat-Noorhidayah

Investigation: Mamat-Noorhidayah**,** Norma-Rashid Yusoff

Methodology: Mamat-Noorhidayah, Norma-Rashid Yusoff

Project administration: Norma-Rashid Yusoff

Supervision: Norma-Rashid Yusoff

Visualization: Mamat-Noorhidayah, Noor Azrizal-Wahid, Van Lun Low

Writing – original draft: Mamat-Noorhidayah, Noor Azrizal-Wahid

Writing – review & editing: Noor Azrizal-Wahid, Van Lun Low

Formatted: Font: (Default) Times New Roman, 18 pt, Bold, Font color: Black

Formatted: Normal, Line spacing: Double, No bullets or numbering

Formatted: Font: (Default) Times New Roman, 18 pt, Bold, Font color: Black

Formatted: Font: (Default) Times New Roman, 12 pt, Bold

Formatted: Normal, No bullets or numbering

Formatted: Font: (Default) Times New Roman, 12 pt, Bold, Italic

Formatted: Font: (Default) Times New Roman, 12 pt, Bold

Formatted: Font: (Default) Times New Roman, 12 pt, Bold, Italic

Formatted: Font: (Default) Times New Roman, 12 pt, Bold

Formatted: Font: (Default) Times New Roman

Formatted: Font: (Default) Times New Roman, 12 pt

Ref. No. (Manuscript ID): PONE-D-23-28970

Manuscript Title:

Genetic diversity and phylogeographic patterns of the peacock jewel-damselfly, *Rhinocypha fenestrella* (Rambur, 1842)

Dear Editor,

Thank you for the careful consideration of our manuscript. We have made changes to the manuscript and responded below to all of the comments by the journal editors and reviewers. The revised manuscript is attached for your further consideration. Please find below a point-by-point response to the comments.

No.	Journal Requirement (Editor's comments)	Action	Authors' remarks
1.	Please ensure that your manuscript meets PLOS ONE's style requirements, including those for file naming. The PLOS ONE style templates can be found at	Responded	> The style requirement for required files is corrected accordingly.
2.	Some minor occurrences of overlapping text with the following previous publication(s), which needs to be addressed:<br><br>https://www.nature.com/articles/s41598-019-45123-0?code=09df2bbc-e733-4183-ad07-99f2eea3b745&error=cookies_not_supported<br><br>In your revision ensure you cite all your sources (including your own works), and quote or rephrase any duplicated text outside the methods section. Further consideration is dependent on these concerns being addressed.	Amended	> The appropriate citations were added accordingly into the referring text. Some texts were also being rephrased.
3.	The grant information you provided in the 'Funding Information' and 'Financial Disclosure' sections do not match. When you resubmit, please ensure that you provide the correct grant numbers for the awards you received for your study in the 'Funding Information' section.	Amended	> The corrections have been made accordingly.

4.	Thank you for stating the following financial disclosure: "Author who received the funding award= Norma-Rashid Yusoff; Grant numbers awarded to the author= 1; The full name of funder= The Universiti Malaya Research Grant (UMRG) PG065 – 2015 and ST061-2022;<br><br>Please state what role the funders took in the study. If the funders had no role, please state: The funders had no role in study design, data collection and analysis, decision to publish, or preparation of the manuscript.<br><br>If this statement is not correct you must amend it as needed. Please include this amended Role of Funder statement in your cover letter; we will change the online submission form on your behalf.	Amended	> The corrections have been made accordingly. The related information on role of the funders were provided.
5.	We note that you have provided funding information that is not currently declared in your Funding Statement. However, funding information should not appear in the Acknowledgments section or other areas of your manuscript. We will only publish funding information present in the Funding Statement section of the online submission form.<br>Please remove any funding-related text from the manuscript and let us know how you would like to update your Funding Statement.	Amended	> The corrections have been made accordingly. The information related to funding were removed from the text and were added in the Funding Statement accordingly.
6.	We note that you have stated that you will provide repository information for your data at acceptance. Should your manuscript be accepted for publication, we will hold it until you provide the relevant accession numbers or DOIs necessary to access your data. If you wish to make changes to your Data Availability statement, please describe these changes in your cover letter and we will update your Data Availability statement to reflect the information you provide.	Responded	> Supplementary data/ Supporting information were provided.

7.	Figure 1 in your submission contain [map/satellite] images which may be copyrighted. We require you to either (1) present written permission from the copyright holder to publish these figures specifically under the CC BY 4.0 license, or (2) remove the figures from your submission, (3) supply a replacement figure that complies with the CC BY 4.0 license.	Amended	> The replacement for Figure 1 was provided.
8.	Please include a copy of Tables 1, 2, 3, 4 and 5 which you refer to in your text on pages 3, 6, 7, and 8.	Responded	> All the text cited Tables were included in the manuscript.
9.	Please include captions for your Supporting Information files at the end of your manuscript, and update any in-text citations to match accordingly.	Responded	> The captions for Supporting Information files were included in the manuscript.
10.	Please upload a copy of Supporting Information Figure/Table/etc. S2 Table B, which you refer to in your text on page 21.	Responded	> The copy of Supporting Information Figure/Table/etc. were provided.
	**Reviewers' comment**	**Action**	**Authors' remarks**
**No.**	**Reviewer I**		
1.	MANOVA and Mantel tests should also be performed.	Rebutted	> We highly appreciate the suggestion. Nevertheless, those analyses were excluded as this paper major aims are on the haplotype diversity and population structure studies.
2.	The discussion is somewhat chaotic and not sound, a lot more should be said on one hand, and several repeated statements should be eliminated, also some contradictory opinions.	Amended	> The repeated statements have been rephrased, and some contradictory opinions were replaced.
3.	English should be corrected. At some parts it is simply impossible to understand the text. Also, some strange terminology can be found, like "uncorrected genetic diversity".	Amended	> Some English mistakes, and terminology errors have been corrected as suggested.
	**Reviewer II**		

1.	Authors should also use BOLD Systems for sequences identification	Rebutted	> The use of GenBank database for sequence identification was following to the common practice in previous publications of the similar works. Nevertheless, we highly appreciate for the comments and will take into consideration in our future works.
2.	MUSCLE is better than CLUSTAL X	Rebutted	> The use of CLUSTAL X was a common practice in our research activities regarding DNA sequencing analysis, which also being practiced by other similar studies.
3.	The authors should perform a full phylogenetic analysis using maximum likelihood and Bayesian approaches with comparison to closely related species and detailed analysis.	Rebutted	> Since this study aim is to provide information of genetic diversity and phylogeographic of the species, we therefore focusing on the use of haplotypes and genetic divergence analyses to evaluate the population diversity intraspecies relationships.
4.	The sequence length is given unnecessarily in two different places.	Amended	> The corrections have been made accordingly as suggested.
5.	Number of sequences and the GeneBank numbers are results not methods.	Amended	> The corrections have been made accordingly as suggested.
6.	Comparing genetic distances and Fst with geographic distances between populations should enrich the results.	Rebutted	> We appreciate the suggestion. However, we found that the current analyses were sufficient enough to address the objectives

			of the manuscript. In fact, the analyses were made by referring to our similar previous published articles (Azrizal-Wahid et al., 2020. New insights into the haplotype diversity of the cosmopolitan cat flea Ctenocephalides felis (Siphonaptera: Pulicidae). Veterinary Parasitology).
7.	Sites should be marked on map, this same for haplotypes, or maybe mOTUs?	Amended	> The corrections have been made accordingly with specific marks of sites were added into the map as suggested.
8.	Some linguistic corrections should be made.	Amended	> The corrections for some linguistic errors have been made accordingly as suggested, throughout the manuscript.

**No.**	**Comments on the text**	**Changes made in the text**
1.	Add author name and year to the mentioned scientific names.	> All the scientific names at their first mention have been added with the author's name and year as suggested.
2.	Page 2, line 13-14: State what the meaning of "more than"...more than what?	> The statement has been rephrased.
3.	Page 2, line 22: Rephrase the sentences.	> The sentences have been rephrased.
4.	Page 3, line 16-18: Rephrase the sentences.	> The sentences have been rephrased.
5.	Page 3, line 21: Change the sub-title "Animal material"	> The sub-title has been replaced into "Sample Collection".
6.	Page 4, line 2-3: in my opinion, at least fixation technique should be presented.	> The general methods of sample collection, preservation was added into the text.
7.	Page 5, line 6-9: Move to the result section.	> The sentences were moved into the result section as suggested.

8.	Page 5, line 24-25: AMOVA should be also performed, perhaps with ARLEQUIN.	> The analyses were excluded as this paper major aims are on the haplotype diversity and population structure studies.
9.	Page 7, line 22-24: Please clarify the meaning of this statement??	> The consideration of Negeri Sembilan as origin of common ancestor haplotype for this study is based on the present of highest numbers of common haplotype found in this site.
10.	Page 8, line 23-24: either extremely mobile, or varying levels of dispersal abilities, you should choose.	> The sentence 'extremely mobile' was removed from the text.
11.	Page 9, line 11-17: These are results, not discussion.	> Here, we are trying to discuss and compare our genetic structures with the other insect mitochondrial genes.
12.	Page 9, line 11-14: COI is more diverse than 16S, thus these parameters should be higher for the COI alone - you should rather compare this locus in the two studies.	> More study examples were added into the text for better comparison of the genetic diversity.
13.	Page 9, line 20-21: You have listed 26 and ten haplotypes above in the text. So why ten and eight here?	> The corrections have been made for the typing mistakes.
14.	Page 9, line 22-25: Rephrase the entire paragraph and add references to the statement on polymorphism.	> The paragraph has been rephrased according and the references regarding polymorphism were added into the text accordingly.
15.	Page 11, line 10-12: ……..genetic differentiation happened……. >what does it mean?	> The statement has been rephrased for better clarification.
16.	Page 12, line 10-11: ……intraspecific patters of haplotypes…… > which pattern refers to?	> The statement has been rephrased for better clarification.
17.	Page 12, line 19-20: These countries are still in the same continent.	> The statement has been corrected accordingly.
18.	Page 12, line 21: How additional sequences may be considered as gap of knowledge?	> The statement has been rephrased for better clarification.
