# Supplementary material for: Genetic diversity and phylogeographic patterns of the peacock jewel-damselfly, Rhinocypha fenestrella (Rambur, 1842)
Source: PLoS One. 2024 Apr 5;19(4):e0301392. doi: 10.1371/journal.pone.0301392 (PMC10997100; doi:10.1371/journal.pone.0301392)
Supplement: Attachment — Submitted filename: Response to Reviewers.docx [file pone.0301392.s005.docx]

Ref. No. (Manuscript ID): PONE-D-23-28970

**Manuscript Title:**

**Genetic diversity and phylogeographic patterns of the peacock jewel-damselfly, *Rhinocypha fenestrella* (Rambur, 1842)**

Dear Editor,

Thank you for the careful consideration of our manuscript. We have made changes to the manuscript and responded below to all of the comments by the journal editors and reviewers. The revised manuscript is attached for your further consideration. Please find below a point-by-point response to the comments.

| **No.** | **Journal Requirement (Editor’s comments)** | **Action** | **Authors’ remarks** |
| --- | --- | --- | --- |
| 1. | Please ensure that your manuscript meets PLOS ONE's style requirements, including those for file naming. The PLOS ONE style templates can be found at | Responded | > The style requirement for required files is corrected accordingly. |
| 2. | Some minor occurrences of overlapping text with the following previous publication(s), which needs to be addressed:  https://www.nature.com/articles/s41598-019-45123-0?code=09df2bbc-e733-4183-ad07-99f2eea3b745&error=cookies_not_supported  In your revision ensure you cite all your sources (including your own works), and quote or rephrase any duplicated text outside the methods section. Further consideration is dependent on these concerns being addressed. | Amended | > The appropriate citations were added accordingly into the referring text. Some texts were also being rephrased. |
| 3. | The grant information you provided in the ‘Funding Information’ and ‘Financial Disclosure’ sections do not match. When you resubmit, please ensure that you provide the correct grant numbers for the awards you received for your study in the ‘Funding Information’ section. | Amended | > The corrections have been made accordingly. |
| 4. | Thank you for stating the following financial disclosure: "Author who received the funding award= Norma-Rashid Yusoff;   Grant numbers awarded to the author= 1; The full name of funder= The Universiti Malaya Research Grant (UMRG) PG065 – 2015 and ST061-2022;  Please state what role the funders took in the study.  If the funders had no role, please state: The funders had no role in study design, data collection and analysis, decision to publish, or preparation of the manuscript.  If this statement is not correct you must amend it as needed. Please include this amended Role of Funder statement in your cover letter; we will change the online submission form on your behalf. | Amended | > The corrections have been made accordingly. The related information on role of the funders were provided. |
| 5. | We note that you have provided funding information that is not currently declared in your Funding Statement. However, funding information should not appear in the Acknowledgments section or other areas of your manuscript. We will only publish funding information present in the Funding Statement section of the online submission form.  Please remove any funding-related text from the manuscript and let us know how you would like to update your Funding Statement. | Amended | > The corrections have been made accordingly. The information related to funding were removed from the text and were added in the Funding Statement accordingly. |
| 6. | We note that you have stated that you will provide repository information for your data at acceptance. Should your manuscript be accepted for publication, we will hold it until you provide the relevant accession numbers or DOIs necessary to access your data. If you wish to make changes to your Data Availability statement, please describe these changes in your cover letter and we will update your Data Availability statement to reflect the information you provide. | Responded | > Supplementary data/ Supporting information were provided. |
| 7. | Figure 1 in your submission contain [map/satellite] images which may be copyrighted. We require you to either (1) present written permission from the copyright holder to publish these figures specifically under the CC BY 4.0 license, or (2) remove the figures from your submission, (3) supply a replacement figure that complies with the CC BY 4.0 license. | Amended | > The replacement for Figure 1 was provided. |
| 8. | Please include a copy of Tables 1, 2, 3, 4 and 5 which you refer to in your text on pages 3, 6, 7, and 8. | Responded | > All the text cited Tables were included in the manuscript. |
| 9. | Please include captions for your Supporting Information files at the end of your manuscript, and update any in-text citations to match accordingly. | Responded | > The captions for Supporting Information files were included in the manuscript. |
| 10. | Please upload a copy of Supporting Information Figure/Table/etc. S2 Table B, which you refer to in your text on page 21. | Responded | > The copy of Supporting Information Figure/Table/etc. were provided. |
|  | | | |
|  | **Reviewers’ comment** | **Action** | **Authors’ remarks** |
| **No.** | **Reviewer I** |  |  |
| 1. | MANOVA and Mantel tests should also be performed. | Rebutted | > We highly appreciate the suggestion. Nevertheless, those analyses were excluded as this paper major aims are on the haplotype diversity and population structure studies. |
| 2. | The discussion is somewhat chaotic and not sound, a lot more should be said on one hand, and several repeated statements should be eliminated, also some contradictory opinions. | Amended | > The repeated statements have been rephrased, and some contradictory opinions were replaced. |
| 3. | English should be corrected. At some parts it is simply impossible to understand the text. Also, some strange terminology can be found, like "uncorrected genetic diversity". | Amended | > Some English mistakes, and terminology errors have been corrected as suggested. |
|  | **Reviewer II** |  |  |
| 1. | Authors should also use BOLD Systems for sequences identification | Rebutted | > The use of GenBank database for sequence identification was following to the common practice in previous publications of the similar works. Nevertheless, we highly appreciate for the comments and will take into consideration in our future works. |
| 2. | MUSCLE is better than CLUSTAL X | Rebutted | > The use of CLUSTAL X was a common practice in our research activities regarding DNA sequencing analysis, which also being practiced by other similar studies. |
| 3. | The authors should perform a full phylogenetic analysis using maximum likelihood and Bayesian approaches with comparison to closely related species and detailed analysis. | Rebutted | > Since this study aim is to provide information of genetic diversity and phylogeographic of the species, we therefore focusing on the use of haplotypes and genetic divergence analyses to evaluate the population diversity intraspecies relationships. |
| 4. | The sequence length is given unnecessarily in two different places. | Amended | > The corrections have been made accordingly as suggested. |
| 5. | Number of sequences and the GeneBank numbers are results not methods. | Amended | > The corrections have been made accordingly as suggested. |
| 6. | Comparing genetic distances and Fst with geographic distances between populations should enrich the results. | Rebutted | > We appreciate the suggestion. However, we found that the current analyses were sufficient enough to address the objectives of the manuscript. In fact, the analyses were made by referring to our similar previous published articles (Azrizal-Wahid et al., 2020. New insights into the haplotype diversity of the cosmopolitan cat flea Ctenocephalides felis (Siphonaptera: Pulicidae). Veterinary Parasitology). |
| 7. | Sites should be marked on map, this same for haplotypes, or maybe mOTUs? | Amended | > The corrections have been made accordingly with specific marks of sites were added into the map as suggested. |
| 8. | Some linguistic corrections should be made. | Amended | > The corrections for some linguistic errors have been made accordingly as suggested, throughout the manuscript. |
|  | | | |
| **No.** | **Comments on the text** | **Changes made in the text** | |
| 1. | Add author name and year to the mentioned scientific names. | > All the scientific names at their first mention have been added with the author’s name and year as suggested. | |
| 2. | Page 2, line 13-14: State what the meaning of "more than"...more than what? | > The statement has been rephrased. | |
| 3. | Page 2, line 22: Rephrase the sentences. | > The sentences have been rephrased. | |
| 4. | Page 3, line 16-18: Rephrase the sentences. | > The sentences have been rephrased. | |
| 5. | Page 3, line 21: Change the sub-title “Animal material” | > The sub-title has been replaced into “Sample Collection”. | |
| 6. | Page 4, line 2-3: in my opinion, at least fixation technique should be presented. | > The general methods of sample collection, preservation was added into the text. | |
| 7. | Page 5, line 6-9: Move to the result section. | > The sentences were moved into the result section as suggested. | |
| 8. | Page 5, line 24-25: AMOVA should be also performed, perhaps with ARLEQUIN. | > The analyses were excluded as this paper major aims are on the haplotype diversity and population structure studies. | |
| 9. | Page 7, line 22-24: Please clarify the meaning of this statement?? | > The consideration of Negeri Sembilan as origin of common ancestor haplotype for this study is based on the present of highest numbers of common haplotype found in this site. | |
| 10. | Page 8, line 23-24: either extremely mobile, or varying levels of dispersal abilities, you should choose. | > The sentence 'extremely mobile' was removed from the text. | |
| 11. | Page 9, line 11-17: These are results, not discussion. | > Here, we are trying to discuss and compare our genetic structures with the other insect mitochondrial genes. | |
| 12. | Page 9, line 11-14: COI is more diverse than 16S, thus these parameters should be higher for the COI alone - you should rather compare this locus in the two studies. | > More study examples were added into the text for better comparison of the genetic diversity. | |
| 13. | Page 9, line 20-21: You have listed 26 and ten haplotypes above in the text. So why ten and eight here? | > The corrections have been made for the typing mistakes. | |
| 14. | Page 9, line 22-25: Rephrase the entire paragraph and add references to the statement on polymorphism. | > The paragraph has been rephrased according and the references regarding polymorphism were added into the text accordingly. | |
| 15. | Page 11, line 10-12: ……..genetic differentiation happened……. > what does it mean? | > The statement has been rephrased for better clarification. | |
| 16. | Page 12, line 10-11: ……intraspecific patters of haplotypes…… > which pattern refers to? | > The statement has been rephrased for better clarification. | |
| 17. | Page 12, line 19-20: These countries are still in the same continent. | > The statement has been corrected accordingly. | |
| 18. | Page 12, line 21: How additional sequences may be considered as gap of knowledge? | > The statement has been rephrased for better clarification. | |
