# Supplementary material for: Genetic diversity and phylogeographic patterns of the peacock jewel-damselfly, Rhinocypha fenestrella (Rambur, 1842)
Source: PLoS One. 2024 Apr 5;19(4):e0301392. doi: 10.1371/journal.pone.0301392 (PMC10997100; doi:10.1371/journal.pone.0301392)
Supplement: Attachment — Submitted filename: comments.docx [file pone.0301392.s006.docx]

The manuscript contains very useful information regarding the genetic population structure of the damselfly *Rhinocypha fenestrella* in Malaysia. However, there are several issues that should be addressed by the authors before the manuscript is accepted for publication

1. Include the country where the study was conducted in the title
2. The abstract does not contain a statement of the problem
3. Include in the abstract the implications of these results
4. Page 2, line 16, you are talking about dragonflies - the title is about damselflies - note that damselflies are completely different from dragonflies
5. The introduction is very short – the biology, particularly life history of the studied species is not explained in the introduction
6. Page 4, line 9, what was the concentration of ethanol used to preserve the samples
7. Page 6, line 9 – 12, focus on the p-value. Check whether there Fst values are significant – forget about the classification
8. Correct grammatical errors in page 6, lines 23 – 24
9. Table 2, the measured Hd for Johor, Perak and Selangor based on 16S is zero – this could be attributed to low sample size. Example, looking at Table 3, it appears, you only sampled 11 individuals from Selangor. Do you this this number is enough to give you meaningful comparisons of the indices of genetic diversity between populations?
10. Subtitles in page 6 line 22 and page 8 line 2 have the same meaning
11. State whether the Fst values in Table 4 are significant. If they are not significantly different from zero – then your explanations in page 12, lines 2 – 7 do not make any sense
12. Page 12 line 8, was the Fus’ values significant?
13. Page 12, line 11 to 12, correct grammatical errors
14. Page 12, line 12 to 15, look at the values you are calling high and low – (0.0100 and 0.0010). Are they significantly different from zero? If they are not significantly different from zero, then it suggests that these populations are genetically homogenous. Please – revisit the interpretation of your results
15. Correct grammatical errors in page 12 line 18
16. Page 13, line 21, comparing diversity indices from different markers does not make sense
17. Page 14, line 5, high compared to what?
18. Page 14, line 11, you are comparing different and unrelated species
19. Page 14, line 14 – 15, check the interpretation – it is not correct. Having singletons in a population does not necessarily mean the population is isolated from the others
20. Page 15, lines 7 – 15 requires serious revisions – interpretations given here are not correct
21. Page 16, lines 7 - 8, revisit your interpretations
22. Page 16, lines 12 and 13, interpretations of Tajima values should be rechecked
23. Interpretations of results in page 16, lines 23 – 25 are conflicting each other
24. Phylogenetic analysis of *Rhinocypha fenestrella* is missing in the entire document. Revise the title or do phylogenetic analysis of the studied species. Note - This was advised by the previous reviewer. The author just ignored this comment but his defense of this omission lacks coherence and fails to address the critical need for this analysis. Further attention is warranted to rectify this deficiency and enhance the study's comprehensiveness.
25. What are the implications of this study on the management of aquatic ecosystems?
