# Supplementary material for: Genetic diversity and phylogeographic patterns of the peacock jewel-damselfly, Rhinocypha fenestrella (Rambur, 1842)
Source: PLoS One. 2024 Apr 5;19(4):e0301392. doi: 10.1371/journal.pone.0301392 (PMC10997100; doi:10.1371/journal.pone.0301392)
Supplement: Attachment — Submitted filename: Response to Reviewers-R2.docx [file pone.0301392.s008.docx]

Ref. No. (Manuscript ID): PONE-D-23-28970

**Manuscript Title:**

**Genetic diversity and phylogeographic patterns of the peacock jewel-damselfly, *Rhinocypha fenestrella* (Rambur, 1842)**

Dear Editor,

Thank you for the careful consideration of our manuscript. We have made changes to the manuscript and responded below to all of the comments by the journal editors and reviewers. The revised manuscript is attached for your further consideration. Please find below a point-by-point response to the comments.

|  | **Reviewers’ comment** | **Action** | **Authors’ remarks** |
| --- | --- | --- | --- |
| **No.** | **Reviewer #3** |  |  |
| 1. | Include the country where the study was conducted in the title. | Rebutted | The country name was not added in the title so that it could catch a broad view of readers from various countries or regions. |
| 2. | The abstract does not contain a statement of the problem. | Responded | The problem statement has been added in the abstract as suggested. |
| 3. | Include in the abstract the implications of these results. | Responded | The brief implications of this study have been added in the abstract as suggested. |
| 4. | Page 2, line 16 > you are talking about dragonflies - the title is about damselflies - note that damselflies are completely different from dragonflies. | Amended | The typing errors have been corrected. |
| 5. | The introduction is very short – the biology, particularly life history of the studied species is not explained in the introduction. | Rebutted | The introduction that consists of four paragraphs is seen to be ideal in length. The introduction focuses more into the gap on the importance of the studied species, approaches and scope of the study. |
| 6. | Page 4, line 9 > what was the concentration of ethanol used to preserve the samples. | Amended | The concentration of ethanol is added in the text. We used 80% ethanol for sample preservation. |
| 7. | Page 6, line 9 – 12 > focus on the p-value. Check whether there Fst values are significant – forget about the classification. | Responded | The Fst values were re-analysed, and the significant levels were determined as suggested. |
| 8. | Correct grammatical errors in page 6, lines 23 – 24. | Amended | The grammatical errors have been corrected. |
| 9. | Table 2, the measured Hd for Johor, Perak and Selangor based on 16S is zero – this could be attributed to low sample size. Example, looking at Table 3, it appears, you only sampled 11 individuals from Selangor.  Do you think this number is enough to give you meaningful comparisons of the indices of genetic diversity between populations? | Responded | The sample size used is based on the availability of individuals in particular sampling locations. Some locations have very little number of individuals, therefore limit the sample size. Nevertheless, the use of sample size ranging between 10-15 individuals per population were reported in other Odonata studies (Minot & Husté, 2022; Kohli et al., 2018), and known to be enough to demonstrate the genetic diversity. |
| 10. | Subtitles in page 6 line 22 and page 8 line 2 have the same meaning. | Responded | The subtitle “Genetic diversity” describes more on the genetic parameters such as number of haplotypes (*N*_h_), haplotype diversity (*H*_d_), nucleotide diversity (*P*_i_), the number of segregating sites (*S*), and average number of sequence differences (*K*), while subtitle “Haplotype variation” is more focusing on the presentation of haplotype characteristics, variation, and distribution. |
| 11. | State whether the Fst values in Table 4 are significant. If they are not significantly different from zero – then your explanations in page 12, lines 2 – 7 do not make any sense. | Responded | The explanation given in Page 12, line 2-7 is mentioning the range values of the p-distance only. Nevertheless, The Fst values were re-analysed, and the significant levels were determined in which most are showing significant different. |
| 12. | Page 12 line 8, was the Fu’ s values significant? | Responded | All the generated Fu’s values in our study are not statistically significant (p>0.05) |
| 13. | Page 12, line 11 to 12, correct grammatical errors. | Amended | The grammatical errors have been corrected. |
| 14. | Page 12, line 12 to 15 > look at the values you are calling high and low – (0.0100 and 0.0010). Are they significantly different from zero?  If they are not significantly different from zero, then it suggests that these populations are genetically homogenous. Please – revisit the interpretation of your results. | Responded | The Fst values were re-analysed, and the significant levels were determined in which most are showing significant different which concordant with the interpretation. |
| 15. | Correct grammatical errors in page 12, line 18. | Amended | The grammatical errors have been corrected. |
| 16. | Page 13, line 21 > comparing diversity indices from different markers does not make sense. | Amended | The comparison statement using different molecular marker was removed from the text. |
| 17. | Page 14, line 5 > high compared to what? | Amended | The sentence has been rephrased for better clarity. |
| 18. | Page 14, line 11 > you are comparing different and unrelated species. | Responded | This comparison was made due to no or limited previous studies done on the same species. Therefore, we did comparison with the closest species as possible that available in literatures. |
| 19. | Page 14, line 14 – 15 > check the interpretation – it is not correct. Having singletons in a population does not necessarily mean the population is isolated from the others. | Responded | The statement does not indicate the population is isolated but might be separated by several geographical barriers that limit the gene flow, therefore had caused increase genetic differentiation including the existence of the singleton. |
| 20. | Page 15, lines 7 – 15 > requires serious revisions – interpretations given here are not correct. | Responded | Here, we did comparison the genetic distance of our studied species with other species that are nearly related since lack of previous studies conducted on the same species. The comparison was made based on species either from the same group (damselflies) or same Order (Odonata). |
| 21. | Page 16, lines 7 – 8 > revisit your interpretations. | Responded | The interpretations have been corrected. |
| 22. | Page 16, lines 12 and 13 > interpretations of Tajima values should be rechecked. | Responded | The interpretations have been rechecked and corrected. |
| 23. | Interpretations of results in page 16, lines 23 – 25 are conflicting each other. | Responded | The sentences have been rephrased/ corrected for better clarity. |
| 24. | Phylogenetic analysis of *Rhinocypha fenestrella* is missing in the entire document. Revise the title or do phylogenetic analysis of the studied species.  Note - This was advised by the previous reviewer. The author just ignored this comment, but his defense of this omission lacks coherence and fails to address the critical need for this analysis. Further attention is warranted to rectify this deficiency and enhance the study's comprehensiveness. | Responded | Thank you for the suggestion. However, from our understanding, phylogeographic and phylogenetic analyses both have their own aims. Since in our title, we are focusing more on the phylogeography, thus, the analyses provided on genetic diversity, haplotype variations and networks are strong enough to support the study aims. |
| 25. | What are the implications of this study on the management of aquatic ecosystems? | Responded | The implications of this study have been added into the text. |
| **No.** | **Reviewer #4** |  |  |
| 1. | Why did the author use a concatenated sequence for their analysis? All the discussion and interpretation of the results were based on concatenated sequences. | Responded | The use of concatenated sequences is to provide more information on polymorphisms sequence variation using longer nucleotide length (if combine). This practice was also being reported in many other similar studies on genetic diversity. |
| 2. | In Table 2, The number of sequences (N) should also be used in each sampling site for each marker. | Amended | The information on the number of sequences was added into the Table 2 as suggested. |
| 3. | In Table 3, edit (n) the number of sequences. Also, they should mention the number of haplotypes for each site on the right side of the table. | Amended | Table 3, ‘n’ has been edited as number of sequences whereas total number of haplotypes for each site were not included as their total is proportionate to the total number of sequences. |
| 4. | Please discuss the possible reason for the highest number of haplotypes in Pahang. | Responded | The justification for the reason on highest number of haplotypes in Pahang has been added into the text. |
| 5. | Why didn't the authors consider sequences from other countries? They should include other sequences from neighbouring countries to show the gene flow and genetic diversity between Malaysia and other countries. | Responded | This study is trying to highlight the pattern of population structure among Malaysian populations for *R. fenestrella* since lack or no record of genetic database specifically for the studied species out from Malaysia, available in database (e.g. GenBank). For the truth, this study is the first to provide comprehensive understanding on the *R. fenestrella* species. |
| 6. | They should also use a phylogenetic tree to illustrate their result better. | Responded | > See above justification for the response to the comment/suggestion. |
| **No.** | **Reviewer #5** |  |  |
| 1. | I think instead of showing only haplotype network (Figure 2-4), It must be nice if authors can add the map showing haplotype distribution in each figure. It will be easy to see clearly about the haplotype diversity and promotion in each population. | Responded | > The haplotype diversity and variation for each population have been given in Figure 1. For other Figures of haplotype network, the distribution of haplotypes according to population was indicated by the different of colours. |
| 2. | As I found author responded to one of reviewer that this paper aim to emphasize on genetic diversity and population structure analysis. I think AMOVA analysis is really needed to be done. Also mismatch distribution or skyline plot should be calculated to see demographic history of this species in Malaysia too. | Responded | The AMOVA and mismatch distribution analyses have been added into the manuscript as suggested. |
| 3. | I don't think DNAsp is suitable to use for Fst calculations as it did not provide significant level. Please rerun Fst in Alrequin or GenALEx. | Responded | The Fst values were recalculated (using Arlequin) and were provided with the significant level as suggested. |
| 4. | Author mentioned low gene flow, but table 4 show very low genetic differentiation between the population. I think it is incongruous. Please reanalyze. | Responded | We have amended the errors. The Fst values were re-analysed, and the data showed among the populations, there are great genetic differentiation which congruent with the low gene flow as obtained from this study. |
| **No.** | **Reviewer #6** |  |  |
| 1. | The manuscript entitled “Genetic diversity and phylogeographic patterns of the peacock jewel-damselfly, *Rhinocypha fenestrella* (Rambur, 1842)” by Mamat-Noorhidayah et al. demonstrated the study on population structure and haplotype diversity of the damselfly, *R. fenestrella*. The revised manuscript has been made accordingly to the editor and reviewer’s comments. The current form of the paper is well written with acceptable English present. The rationale, scientific logic, methods, and analysis of data are appropriate. | Responded | We would like to thank to reviewer for acknowledge our great efforts on preparing this manuscript. We highly appreciated the comments. |
| **No.** | **Reviewer #7** |  |  |
| 1. | Deviation from a standard neutral model is restrictive per gene and more informative when each gene is analyzed separately. Concatenated data may give a false impression of Tajima’s D, Fu’s and Li, Fu’s or Fu and Li’s D*, Fu and Li’s F*, and Fu’s FS statistic. | Responded | The use of concatenated sequences for determination of Tajima’s D and Fu’s statistics was commonly practiced in our previous similar studies (Azrizal-Wahid et al., 2020) that showed reliable outputs regarding the genetic diversity. |
| 2. | (Abstract) Page 1, Line 24-25 > needs to be rephrased for clarity. | Amended | > The sentence has been amended for better clarification. |
|  | Methodology |  |  |
| 3. | Confirm the 300 sec on PCR protocol. | Amended | > The typing mistake has been corrected. |
| 4. | …..The aligned cox1 and 16S sequences at first were analysed respectively… > …were analysed separately…. | Amended | > The word has been corrected accordingly as suggested. |
| 5. | The FST and Nm pairwise values were calculated to access genetic differentiation and gene flow among the *R. fenestrella* population and were calculated using DNASP®… Rephrase the sentence for clarity. | Amended | > The sentence has been rephrased. |
|  | Discussion |  |  |
| 6. | …..The obtained sequences for cox1 and 16S genes from eight populations ranged from 1.5……….. > Rephrase the sentence for clarity. | Amended | > The sentence has been rephrased. |
| 7. | In this study, the number of haplotypes detected was considered high with 26 haplotypes were revealed by cox1 and 10 haplotypes by 16S. Rephrase the sentence for clarity. | Amended | > The sentence has been rephrased for better clarification. |
| 8. | Page 14 Line 6-7 > The high number is due to and not “may indicate” ..... It is an indicative of high diversity in species. | Amended | The phrase has been rewrite as suggested. |
| 9. | Page 14 Line 14 > …..with singleton sites…….. also include Reference attributing presence of singleton sites to current genetic differentiation. | Amended | The phrase has been rewrite as suggested. |
| 10. | …..to star-like radiation in……This is not an indicator of common ancestry…….Is a common ancestry due divergence of haplotype from its polymorphic sequence. | Amended | The sentence has been rephrased for better explanation. |
| 11. | ….Likewise, according to location20 based, the most recent common ancestor for R. fenestrella in Malaysia may be derived from Negeri Sembilan as this state is constituted by high frequencies of the common ancestor AB1 haplotypes. Rephrase the sentence to avoid repetition. | Amended | The sentence has been rephrased for better clarity. |
| 12. | ….The data shows a higher genetic distance among Malaysian *R. fenestrella* as compared to the populations of *Rhinocypha taiwana* (Wang & Chang, 2013), *Rhinocypha uenoi* (Asahina, 1964), and *Rhinocypha drusilla* (Needham, 1930) from China where the highest genetic distances based on cox1 gene were 0.00%, 0.00% and 0.50%, respectively……….0.00% is not high | Responded | What the authors trying to mention here is that the genetic distance for Malaysia populations is higher compared to other populations from other regions. Nevertheless, some of the phrases have been rewrite for better clarity. |
| 13. | …cox1 gene of up to 9.00% and 12.00%, respectively… | Amended | The sentence has been rephrased as suggested. |
| 14. | …..Zygopterans are known as weaker fliers since many species do not diffusing outside several kilometres……rewrite for clarity | Amended | The sentence has been rephrased for better clarity. |
